# Developmentally dependent reprogramming of the *Arabidopsis* floral transcriptome under sufficient and limited water availability

**DOI:** 10.1186/s12870-024-04916-w

**Published:** 2024-04-11

**Authors:** Xinwei Ma, Jun Wang, Zhao Su, Hong Ma

**Affiliations:** 1 Department of Biology, Pennsylvania State University, University Park, PA 16802 USA; 2 Laboratory of Plant Stress and Development, College of Life Sciences, University of Chinese Academy of Sciences, Beijing, 100049 China

**Keywords:** *Arabidopsis*, Reproductive development, Drought stress, RNA-seq

## Abstract

**Background:**

Environmental stresses negatively impact reproductive development and yield. Drought stress, in particular, has been examined during *Arabidopsis* reproductive development at morphological and transcriptomic levels. However, drought-responsive transcriptomic changes at different points in reproductive development remain unclear. Additionally, an investigation of the entire transcriptome at various stages during flower development is of great interest.

**Results:**

Here, we treat *Arabidopsis* plants with well-watered and moderately and severely limiting water amounts when the first flowers reach maturity and generate RNA-seq datasets for early, middle, and late phases during flower development at 5, 6, and 7 days following treatment. Under different drought conditions, flowers in different developmental phases display differential sets of drought-responsive genes (DTGs), including those that are enriched in different GO functional categories, such as transcriptional regulation and response to stresses (early phase), lipid storage (middle phase), and pollen and seed development and metabolic processes (late phase). Some gene families have different members induced at different floral phases, suggesting that similar biochemical functions are carried out by distinct members. Developmentally-regulated genes (DVGs) with differential expression among the three floral phases belong to GO terms that are similar between water conditions, such as development and reproduction, metabolism and transport, and signaling and stress response. However, for different water conditions, such similar GO terms correspond to either distinct gene families or different members of a gene family, suggesting that drought affects the expression of distinct families or family members during reproductive development. A further comparison among transcriptomes of tissues collected on different days after treatment identifies differential gene expression, suggesting age-related genes (ARGs) might reflect the changes in the overall plant physiology in addition to drought response and development.

**Conclusion:**

Together, our study provides new insights into global transcriptome reprogramming and candidate genes for drought response, flower development, aging and coordination among these complex biological processes.

**Supplementary Information:**

The online version contains supplementary material available at 10.1186/s12870-024-04916-w.

## Introduction

Abiotic stresses, including water, temperature, light radiation, and nutrients [1], have resulted in severe constraint in agriculture worldwide. Drought stress in particular, can cause physiological damage to crop plants and thus lead to dramatic yield loss [2]. For example, drought has caused 11.9% agricultural damage and $290.7 billion in disaster costs over the past four decades in the US [3]. The effect of drought stress on plant vegetative development has been investigated in numerous studies, including but not limited to inhibition of photosynthesis, reduced root growth, decreased leaf area and biomass, and early senescence [2, 4–6]. The cellular and molecular responses under drought stress feature ABA signaling module and central roles of kinase cascades. Subsequently, expression of stress-responsive genes, ion homeostasis, metabolism, and stomata opening are altered to facilitate adaptation to and survival through drought periods while minimizing the damage caused by water deficiency [1, 5–7].

Plants are especially vulnerable to abiotic stresses during reproductive development [8, 9]. Drought stress can cause changes in flowering time and developmental defects in flowers on the main stem, including arrested flower development, reduced total number of flowers and fewer seeds per silique, thus significantly impacting yield [8, 10, 11]. The response to drought during reproductive development also involves changes in the ABA signaling pathway, transcriptional and epigenetic regulation, ion and osmotic homeostasis, and other cellular processes, which together are referred to as the drought response module [8]. For example, *ANAC019* that encodes a putative transcription factor (TF) is important for both floral organ development and drought tolerance, as the *anac019* mutant displayed shortened stamen and pistil and extended acclimation period after drought treatment [12]. Also, *MYB37* was similarly shown to be involved in both seed development and drought response [13].

Transcriptomic analyses using microarrays of drought-treated *Arabidopsis* flowers revealed that the expression level of both developmentally-regulated genes and stress-responsive genes were changed under drought stress, providing information on candidate genes for further functional analyses [10, 11]. Over 4000 differentially expressed genes (DEGs) were identified, including flowering time genes, anther and ovule development genes, and genes responsive to severe drought during vegetative development; mutant analyses confirmed the potential roles of *DREB1A* and *MYB21* in regulating both drought response and flower development [11]. Furthermore, a study using moderate drought condition revealed that, although reproductive morphology appeared minimally affected, almost 2000 genes were differential expressed, including a subset that was unique to moderate drought [10]. However, the microarrays used in the previous studies did not include all annotated *Arabidopsis* genes, thus analyses using RNA-sequencing can yield additional information about transcriptomic changes under drought. Also previous drought-related studies used the whole inflorescence (all the unopened flower buds) with different developmental stages [10, 11]. In these samples, the older buds were much larger than the younger ones and were over-represented in the RNA sample. Thus, the question remains whether flowers at different developmental stages display different sensitivity to drought stress at the transcriptomic level.

In *Arabidopsis*, flower development before opening was divided into 12 stages with morphological characteristics; specifically, stages 1–5 involve floral meristem and organ primordia initiation, stages 5–8 with organ morphogenesis, 8–9 for meiosis and microsporogenesis, and 10–12 for organ growth and pollen development [14]. Previous studies have analyzed floral transcriptomes using microarrays, focusing on various portions of the developmental sequence; for instance, floral buds from stages 1 to ~9 and stage 12 were separately analyzed using microarrays [15]. Another study analyzed floral buds at multiple stages with microarray analyses to detect differentially expressed genes [16]. Additionally, transcriptome profiling of developing flowers has also been performed using RNA-seq in several other plant species, such as Moso bamboo (*Phyllostachys edulis*) [17], wheat (*Triticum aestivum*) [18], and chickpea (*Cicer arietinum* L.) [19, 20]. RNA-seq can potentially detect differential expression for genes not represented in previous microarray analyses, highlighting the need for RNA-seq analyses of developing *Arabidopsis* floral buds. New analyses can provide candidate genes involved in flower development, especially for different cellular processes during development, such as cell division and differentiation (early stages), specification of meiocytes, meiosis and early microspore development (middle stages), cell expansion, organ maturation and biogenesis of pollen wall (late stages). Also, transcriptomic studies in rice, chickpea, and wheat on drought-treated floral samples at different stages suggested that different floral stages could respond to drought stress differently [21–23]. However, the relationships between developmental stage and water availability on transcriptomic changes are not clear. Thus comparisons for floral expression profiles in different phases of *Arabidopsis* flower development in response to drought are needed and the interplay between development and environmental response should be examined.

Here, we estimated the overall *Arabidopsis* reproductive yield with observations on seed production under different water conditions and found that the seed yield of the side branches was dramatically reduced under severe drought stress, although the number of seeds on the main stem was much less affected. Further, we divided the *Arabidopsis* inflorescence into 3 developmental phases, early (largely organ initiation and morphogenesis), middle (near the time of meiosis), and late (organ growth and gametophyte development). Transcriptomic analyses of these floral phases under different water conditions demonstrate that floral buds in each phase respond to drought stress with largely distinct sets of genes that are enriched in different functional categories. In addition, under either sufficient or limiting water availability, flower buds exhibited differential gene expression among the phases, involving individual genes unique to specific water conditions. Furthermore, plant age also affected the flower transcriptome under each water condition. Together, our study presents rich and valuable resources of gene expression profiles of three flower developmental phases under growth conditions of sufficient or limiting water availability, with differences in plant age, providing numerous candidate genes for understanding relevant plant developmental and physiological processes.

## Results

### *Arabidopsis* yield decreased significantly as the drought severity increased

Previous phenotypic characterizations of *Arabidopsis* reproductive development under drought stress [10, 11] focused on the morphological changes and seed production on the main stem, but the side branches under drought conditions were not described. Therefore, we examined reproductive development for the whole plant, with 10 individual plants in two replications under each of five water conditions: well-watered (WW, ~90% SWC), slight drought (1/2MD, ~75% SWC), moderate drought (MD, ~55% SWC), slightly severe drought (1/2SD, ~45% SWC) and severe drought (SD, ~35% SWC).

We found that the total silique number on the main stem of the plants under different water conditions were similar at ~40 siliques (some were smaller under drought), but the total silique number of the side branches (excluding the main stem) showed a dramatic reduction from ~70 siliques of WW plants to ~40 siliques of SD plants (Figure S1A). Similarly, the total seed number on side branches was also different between plants under different water conditions (Figure S1B). Specifically, silique number and total seed number on side branches both reduced dramatically under MD and SD conditions compared with WW plants: 25% and 40% reduction on the silique number, and 25% and 65% reduction on the seed number under MD and SD, respectively (Figure S1A, B). In addition, there was a decrease of the 1000-seed weight (Figure S1C). Further, the average seed number per silique (Figure S1D) and silique length (Figure S1E) showed a decrease for both the main stem and side branches, suggesting that stress resulted in reduced yield (seed number and weight) on both the main stem and side branches, despite the similar number of siliques on the main stem. Taken together, the effect of drought stress is more obviously seen on the seed number per silique of the main stem and more generally on the side branches, suggesting that even under severe drought stress, plants still devote the available energy and resource to maintain the seed production on the main stem with possible sacrifice of flower development on the branches.

### Floral buds at different developmental phases respond to drought differently

To examine the transcriptomic changes at each of three flower developmental phases among WW and two (MD and SD) drought conditions, we maintained the WW condition for one set of plants and shifted plants to drought conditions when the first flower had opened (see Methods). Five days later, SWC (soil water content) of the SD group reached the desired 35%. Floral buds of early, middle, and late phases were sampled on each of three consecutive days: Day5, Day6 and Day7 for all three water conditions (Day0 WW flowers were sampled as well for comparison) (Figure S2A). In particular, the unopened floral buds were separated according to *Arabidopsis* flower development stages [11, 14] into early (E, ~ stages 1–8), middle (M, ~ stages 8–10), and late (L, ~ stages 10–12) phases (Figure S2B). Totally, 90 samples (30 treatments, 3 replicas for each) were used for RNA isolation and RNA-seq analysis (Figure S2A, C). The biological replicates were highly correlated (Figure S2D), supporting the consistency of the datasets.

**Fig. 1:**
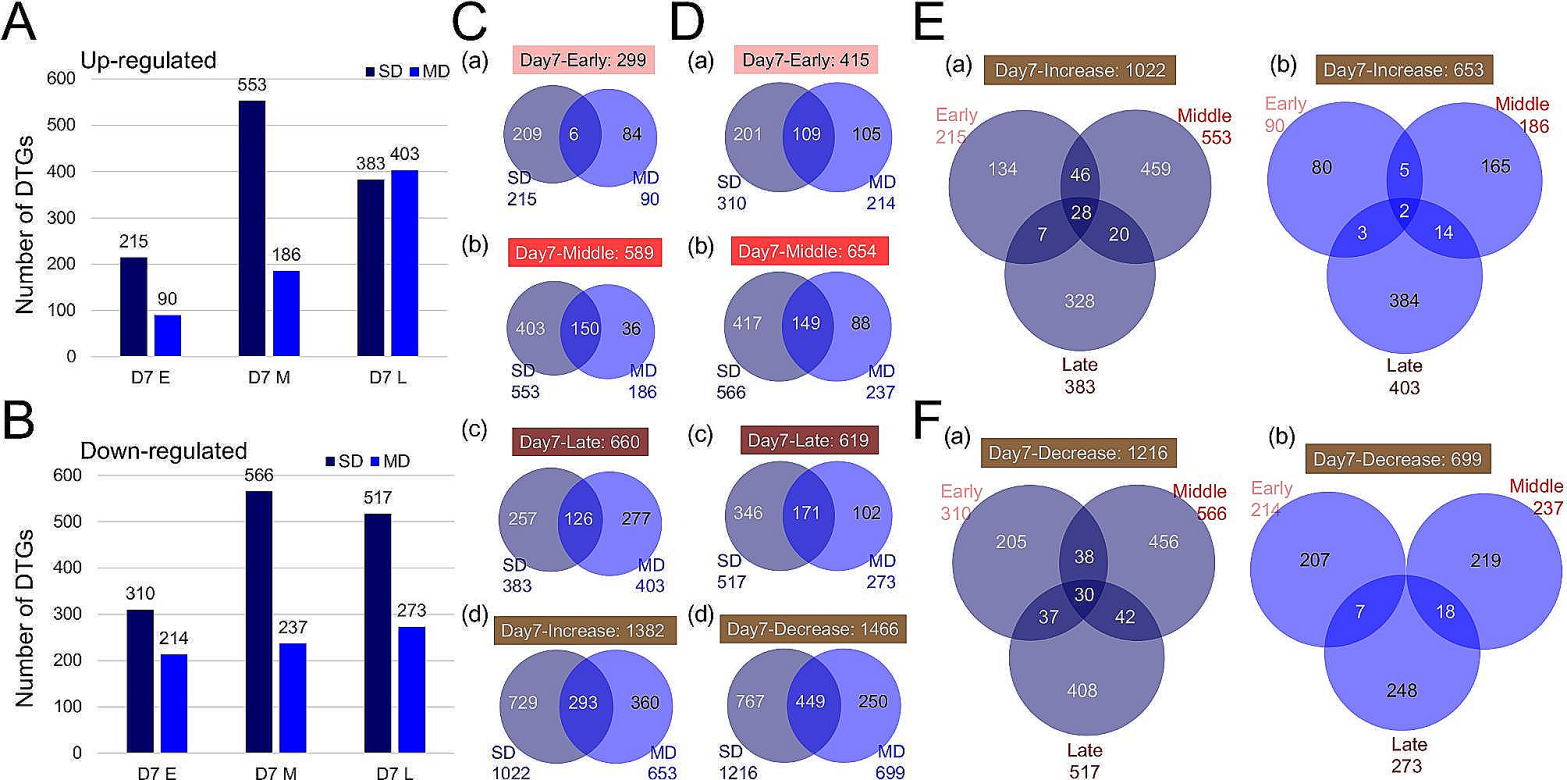
Different degrees of drought and the induced transcriptomic changes. **A-B**. Total number of drought-responsive genes (DTGs) under SD or MD at each developmental phase on Day 7. **A**: up-regulated genes; **B**: down-regulated genes. **C-D**. Comparison of DTGs under SD and MD on Day 7 at each developmental phase and the summarizing comparison including all 3 developmental phases. **C**: up-regulated genes; **D**: down-regulated genes. (a): early phase; (b): middle phase; (c): late phase; (d): DTGs from all 3 phases on Day 7. **E-F**. Comparison of DTGs between the 3 developmental phases on Day 7. **E**: up-regulated genes; **F**: down-regulated genes. (a): SD; (b): MD. Dark blue represents SD (expression level under SD compared to WW), blue represents MD (expression level under MD compared to WW)

We first identified drought-responsive genes (DTGs) that had *p*-value < 0.05 and log_2_ (fold-change of expression level between SD or MD and WW) ≥ 1 or ≤ -1, between WW and SD or MD for each floral developmental phase and on each day (Fig. 1, Figure S3). For example, Day7 early phase flowers showed 215 up-regulated genes under SD (compared with WW) and 90 under MD (vs. WW) (Fig. 1A), and 310 down-regulated genes under SD and 214 under MD (Fig. 1B). For convenience, expression in sample A in comparison with sample B is indicated by “B/A” in this study; up-regulated genes mean genes expressed at higher levels in B than A. Greater numbers of DTGs were found for the Day7 middle phase with 553 (Fig. 1A) and 566 (Fig. 1B) up- and down-regulated genes under SD condition, respectively. Additional numbers of differentially expressed genes are shown in Figure S3A, B. Comparisons of various sets of drought-responsive genes showed that different genes were responsive to different water conditions, with less than 30% overlapping between two conditions (Fig. 1C, D, Figure S3C, D). The combined sets of up- and down-regulated genes under SD and MD also showed the same pattern (Figure S3E), consistent with previous reports using microarray analyses [10]. Next, we compared DTGs among the three flower developmental phases on the same day and found different gene identities of the DTGs at different phases. For instance, among the 1022 up-regulated DTGs under SD on Day7, only 101 DTGs (10%) overlapped between at least two of the three developmental phases (Fig. 1Ea), suggesting that the sampling for three phases were largely separate and each phase exhibit distinct responses to drought. The relatively small overlap of DTGs between developmental phases was similarly observed for MD-responsive genes and down-regulated genes at each of the three days (Fig. 1Eb, Fb, Figure S4A-D), suggesting that this is a general property of drought-affected genes.

To obtain clues about possible/predicted functions of the DTGs, we performed enrichment analysis of GO categories for SD-up-regulated genes from each of three days and found that the 38 overlapping genes (Figure S4C) were mainly enriched in stress response and signaling (Figure S4E: Up-SD-shared), suggesting that the putative role in stress response is shared among DTGs at various developmental phases. In addition, the DTGs unique to a specific developmental phase at any of the three days showed enrichment in reproductive development, primary and secondary metabolism, and transport (Figure S4E: Up-SD-early/middle/late). For DTGs under MD, there were very few genes shared among developmental phases; even the enriched GO terms were different at different phases, although they are generally involved in development and stress response (Figure S4C, E), suggesting that DTGs at different developmental phases have distinct putative functions in similar broad categories. A detailed examination of specific biological functions revealed that specific DTGs with similar biological and molecular functions exhibit distinct patterns of drought-induced expression with regard to developmental phases and days. For example, genes encoding protein degradation related factors, proteinase and peptidase, showed differential expression with respect to developmental phases in response to severe drought, even for members of the same gene family (Figure S4F, G). These observations suggest that different members of the same gene family could function specifically at a certain flower developmental phase, and that protein turnover is likely regulated during flower development under drought. Totally, 2260 genes were up-regulated and 1896 were down-regulated (Figure S3E) in response to either of the two drought stresses at one of the phases or days, with a combined total of 3582 DTGs (Figure S3F, File S1, 2), as some up-regulated genes were also down-regulated for another treatment.

**Fig. 2:**
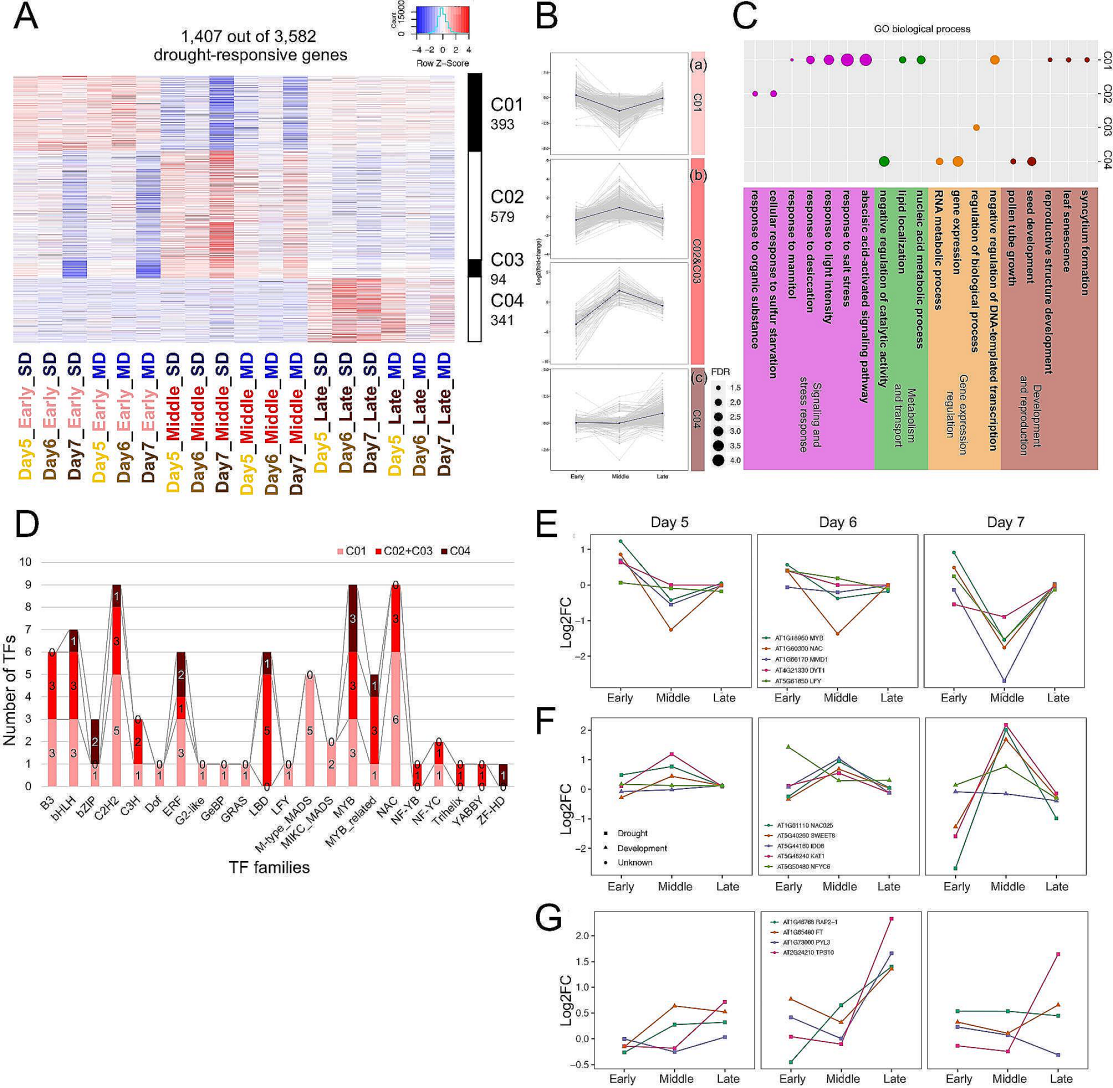
Developmental phase-specific transcriptome reprogramming under drought of representative DTG clusters. **A**. Expression dynamics across the 3 developmental phases and the 3 days of 4 representative clusters (C01-C04) including 1407 DTGs. Red indicates up-regulation, blue indicated down-regulation and white indicates no change. **B**. The fold change of all genes (grey) in the corresponding clusters at each developmental phase under SD on Day 7. The dark blue line represents the average fold change of all genes in the cluster. **C**. GO enrichment based on genes from the corresponding clusters. Colors of the dots refer to different general biological processes, and sizes of the dots refer to the level of enrichment (-log_10_FDR). **D**. The TFs and the TF families that they are from in the corresponding clusters. Light red refers to TFs from C01 (a), red refers to TFs from C02 and C03 (b), and dark red refers to TFs from C04 (c). **E-G**. The fold change of representative genes from the corresponding clusters under SD. **E**. genes from C01; **F**. genes from C02 and C03; **G**. genes from C04. Square indicates that the gene has a previously reported function in drought response, triangle indicates that the gene has a previously reported function in development, and circle indicates that the gene has no published functional study. Each color represents a different gene, though the same color in different graphs does not refer to the same gene

To examine common expression patterns of DTGs, a K-means clustering analysis was conducted with the 3582 DTGs based on the fold-change upon drought treatments, resulting in ten clusters (K = 10). The expression fold changes at the three developmental phases and the 3 days were shown as heatmaps for all the genes in each cluster (Fig. 2A, Figure S5A, File S2); in addition, the mean values of expression fold changes for each cluster are highlighted (Fig. 2B, Figure S5B). Specifically, Cluster 01 (393 genes) showed an obvious reduction at the middle phase across all three days, resulting in a “V”-shaped expression pattern (Fig. 2A, Ba), whereas Cluster 02 (579 genes) and 03 (94 genes) only showed high induction at the middle phase, with an opposite “Λ”-shaped expression pattern (Fig. 2A, Bb). Cluster 04 (341 genes) include DTGs that were specifically induced at the late phase but showed no obvious changes at the other phases (Fig. 2A, Bc). GO enrichment analysis was then conducted for each cluster individually (Fig. 2C): the “V”-shaped C01 was highly enriched in development and various aspects of stress response, such as ABA-signaling pathway, response to salt stress, etc., as well as regulation of transcription, and nucleic acid metabolic process. On the other hand, the “Λ”-shaped C02 & 03 were enriched in response to sulfur starvation and organic substance. For the late-phase-specific C04, enriched GO include reproductive development (e.g., seed development, pollen tube growth and gene expression), RNA metabolism, and regulation catalytic activity. DTGs also belong to other GO categories for biological processes, such as reproductive development, RNA processing, secondary metabolism and stress response (Figure S5C).

In addition, we identified 282 genes encoding putative TFs among the DTGs. We further found that many of these drought-responsive TF-genes belonging to different clusters are different members from the same TF families; for example, bHLH, C2H2, ERF, MYB and MYB-related family members were found in all 4 representative clusters (C01-04) (Fig. 2D). Gene regulatory networks (GRNs) were then constructed based on all the TFs from the representative clusters. All 39 TFs from the “V”-shaped C01 together formed a greatly interrelated and complex network (Fig. 2E, Figure S5D) with *LFY*, *DYT1*, and two genes encoding a NAC and a MYB protein as the central nodes. *LFY* is a central transcriptional regulator of floral meristem identity and early floral organ development [24], while *DYT1* is a key regulator of anther cell differentiation and anther transcriptome *DYT1* [25], suggesting that drought can influence reproductive development through these genes. The *NAC* and *MYB* genes identified here were not known to be involved in either reproductive development or drought response and could be candidates for functional study of these processes. Other TF genes in C01-GRN are also important for development, such as *MYB35* [26] and members of the AP2/B3 family, which includes the stress-responsive ERF subfamily members [27]. Eight of 30 TFs from the “Λ”-shaped C02 & 03 are part of a less complex GRN (Fig. 2F, Figure S5E) with *IDD8* (C2H2 family) and *NF-YC6* as the central nodes that connect multiple LBD and NAC family members with possible roles in reproductive development. Additionally, TFs important for regulating genes for ion transport like *bHLH029* and *bHLH100* [28] were also parts of the GRNs, suggesting possible diverse biological processes in the flower affected by drought.

Among 58 annotated TF families, members from 40 were responsive to drought stress (Fig. 2D, Figure S5F, G), providing clues regarding drought effects on known gene functions and information about potential functions of the genes that are yet to be analyzed genetically. In addition to the above mentioned TF families, B3, bZIP, C3H, and LBD family members were also found in more than half of the clusters (Figure S5G). Moreover, different clusters contain different members of the same TF families and form distinct GRNs. Some genes down-regulated in the middle phase (including meiotic cells) might reflect the sensitivity of meiosis to drought; for instance, *MMD1* from C01 (Fig. 2E) is an important regulator of multiple aspects of male meiosis, including chromosome condensation [29] and was significantly downregulated under stressed conditions specifically at middle stage, but not at early or late phases. On the other hand, *SWEET8* from C02 (Fig. 2F) is involved in pollen wall formation [30] and was significantly upregulated under drought stress specifically at middle stage, implying the importance of enhancing the reproductive program to ensure some fertility under drought stress. Surprisingly, *FT* from C04 (Fig. 2G) is a key factor promoting flowering [31] was significantly upregulated at the late phase, suggesting a potential unexpected role during late flower development.

We also compared DTGs at the same developmental phase among three different days (Figure S6A, B) and found the overlap between any two of the three sets of DTGs was smaller than 25%, suggesting that when the duration of the drought stress extended, more distinct genes were needed for survival and development under adverse environmental conditions (Fig. 1E, F, Figure S4A-D). Similarly, GO enrichment analyses suggest that specific subsets of DTGs on different days induced under SD or MD (Figure S6Aa, Ab) were enriched for categories of gene expression and response to stresses (Figure S6C). In summary, distinct DTGs were identified at three developmental phases and often contained different members of the same gene families, especially those for TFs, which formed different putative GRNs probably important for acclimation to drought stresses at these floral phases.

### Differential gene expression profiles among three developmental phases

**Fig. 3:**
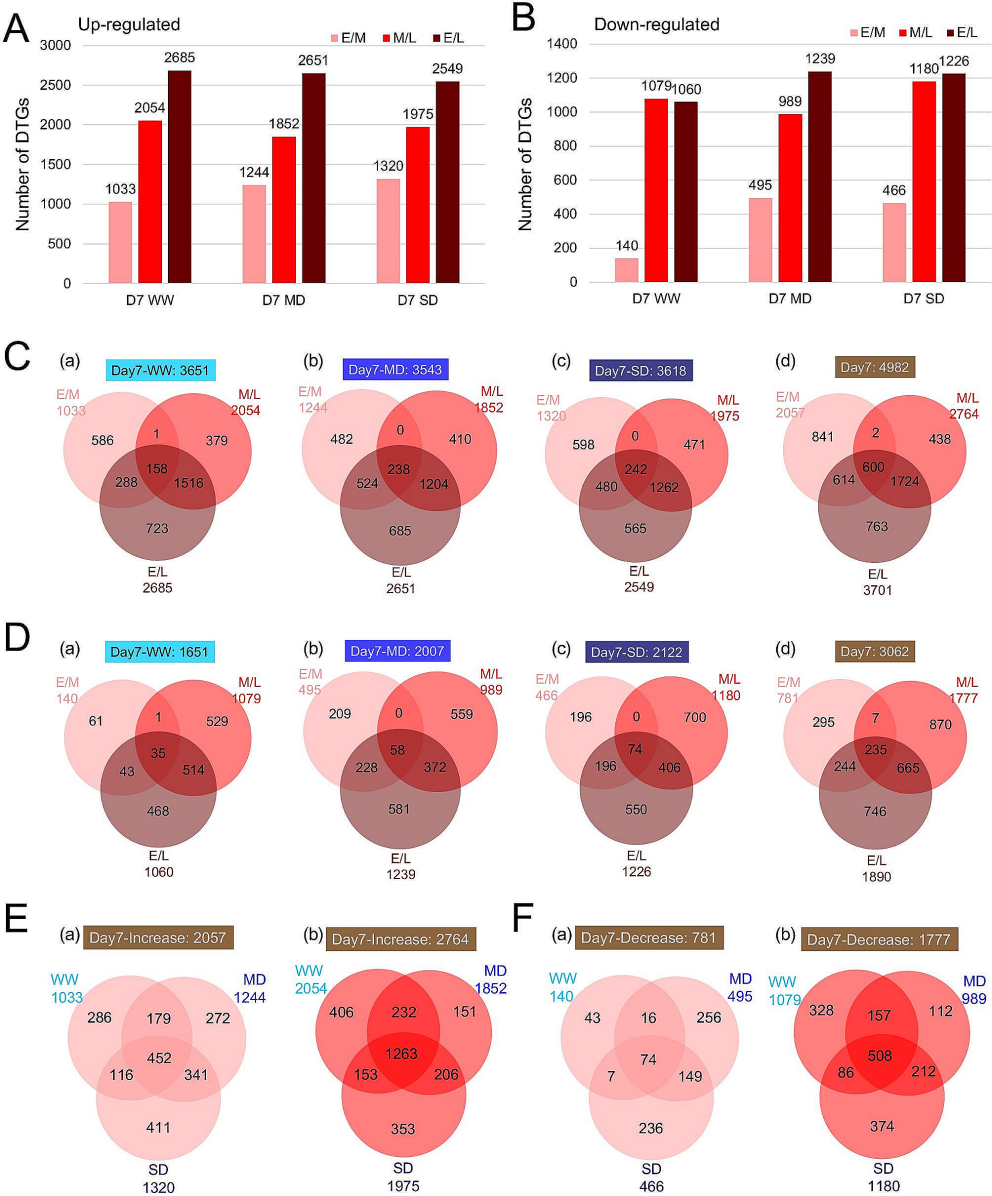
The transcriptomic changes as reproductive development progresses under different water conditions. **A-B**. Total number of developmentally regulated genes (DVGs) in early vs. middle, middle vs. late, and early vs. late phase comparisons under each water condition on Day 7. **A**: up-regulated genes; **B**: down-regulated genes. **C-D**. Comparison between the DVGs from the 3 developmental phase comparisons under the same water condition on Day 7. **C**: up-regulated genes; **D**: down-regulated genes. (a): WW; (b): MD; (c): SD; (d): DVGs from all 3 water conditions. **E-F**. Comparison between the DVGs from the same developmental phase comparison under the 3 different water conditions on Day 7. **E**: up-regulated genes; **F**: down-regulated genes. (a): E/M; (b): M/L. Light red represents E/M (expression level at middle phase compared to early phase), red represents M/L (expression level at late phase compared to middle phase), dark red represents E/L (expression level at late phase compared to early phase)

To obtain clues about functional changes during flower development, we identified differentially expressed genes among three floral phases for the same water conditions and referred to these genes as developmentally-regulated genes (DVGs) using log_2_ (fold-change of expression level between early/middle, middle/late or early/late) ≥ 1 or ≤ -1 and *p*-value < 0.05 as cutoff. Comparing the middle with early phases, there were 1000 to 1400 up-regulated DVGs, and ~400 down-regulated; similarly, 1500 to 2000 DVGs were up-regulated in the late phase compared with the middle phase and ~1000 down-regulated; finally, ~2500 DVGs were up-regulated when comparing the late with early phases, and 1000 to 1200 DVGs were down-regulated (Fig. 3A, B, Figure S7A, B). Thus, the numbers of the DVGs among the phases were comparable among different water conditions and different days. Also, a comparison of the DVGs for the same water conditions (Fig. 3C, D and Figure S7C-E) found very few common genes (less than 7% of all DVGs) overlapped among the three comparisons between phases. For example, only 159 DVGs among 3651 up-regulated under WW on Day7 (Fig. 3Ca) overlapped between the early/middle and middle/late comparisons. Thus DVGs that increased in expression from the early to middle phases were generally different from those increased from the middle to late phases. In contrast, there were more common DVGs between early/late and the other two phase-comparisons (Fig. 3C, D, Figure S7C-E), indicating greater similarity in transcriptome between two closer phases (early vs. middle; middle vs. late) than the more developmentally separate early and late phases. Overall, the extent to which the DVGs overlap between different stage comparisons were comparable under different water conditions (Fig. 3C, D and Figure S7C-E), suggesting that even under stressful conditions, the general patterns of reproductive transcriptomes were similar.

We also investigated the expression level of known flower regulatory genes over the developmental phases under well-watered conditions, to utilize our datasets for further understanding of their functions during flower development. The expression level of ABCDE model genes under well-watered conditions was averaged over the different days, and plotted with respect to the three developmental phases (Figure S7G). Our transcriptomic data demonstrated that the floral homeotic genes [32, 33] were not solely expressed during the early phase with floral organ differentiation, but also expressed at the middle or late stage, suggesting their possible functions during middle and/or late flower development. For example, the C function gene *AGAMOUS* is expressed during the late phase of flower development (Figure S7G) and was reported to be important for stamen and carpel development, including stamen maturation [34]. Our results support the hypothesis that the ABC genes also function during late flower developmental phases, to promote the maturation of the organs that require the genes for the early specification of organ identity.

Next we identified the DVGs that showed differential expression under drought conditions, especially those that did not show differential expression under WW. For example, from the early to middle phases, 105 DVGs were up-regulated (Figure S8A) and 62 were down-regulated (Figure S8B) in at least three of the six drought related conditions, and 1214 DVGs were altered in one or two of the six drought related conditions (Figure S8C). One such DVGs is *WRKY12*, previously reported to be important for flowering time control in *Arabidopsis* [35] and secondary cell wall formation in other plants [36, 37], which showed a “V”-shaped pattern across the three developmental phases under WW, and an enhanced “V”-shaped pattern under MD and SD conditions (Figure S8D). This increased extent of changes of its expression level under drought conditions suggests that some DVGs might need greater changes in expression level under drought conditions to ensure apparently normal reproductive development. GO enrichment analysis revealed that these subsets of DVGs were enriched in several categories related to biological processes and molecular functions, such as development, mRNA processing, protein metabolic process and transport, and response to water deprivation and ABA (Figure S8E).

Although the numbers of DVGs under WW and drought conditions and their patterns of differential expression at different phases are similar under WW and drought conditions, the specific DVGs might be different under different water conditions. To examine the identity of the DVGs for different water conditions, we compared the DVGs on the same day between the three different water conditions. We found that less than half of the DVGs were common for all water conditions (e.g., 452/2057, 22% of up-regulated DVGs from E/M, Fig. 3Ea), whereas one third to two thirds of the DVGs were specific to one water condition (e.g., 969/2057, 47% up-regulated DVGs from E/M, Fig. 3Ea) (Fig. 3E, F, Figure S9A-D). The specific sets of DVGs for distinct water conditions are strong evidence that the apparently similar flower development is not the same at the molecular level. For example, the plants might need additional gene functions to promote normal floral morphologies; at the same time, non-essential genes might have reduced expression under stressful conditions.

GO term analyses found that, among the up-regulated DVGs from the comparisons of three phases (Figure S9C-D), the overlapping genes between all three water conditions and the specific genes to each water condition were enriched for different GO terms (Figure S9E). For instance, for the DVGs specific to SD, DVGs higher in the middle than early phase were enriched in RNA processing, and response to ABA, light, and wounding (Figure S9E: E/M-SD); genes higher at the late than middle phase were enriched in cell differentiation and development, secondary metabolism, response to fatty acid (Figure S9E: M/L-SD); and genes up-regulated at the late phase in comparison with early phase were enriched in developmental growth, gene expression and RNA metabolism, response to hormone stimulus(Figure S9E: E/L-SD). On the other hand, the overlapping genes between the three water conditions were generally enriched in developmental growth, gene expression, primary and secondary metabolism, protein modification and turnover, and response to environmental factors (Figure S9E: E/M, M/L, E/L-shared).

Further inspection of specific families with members among the DVGs revealed that members of some gene (sub)families with broadly similar functions showed differential expression at different phases during reproductive development, or under various water conditions. For instance, members of protein kinase families are some of the DVGs (Figure S10A, B). Members of both MAPK and MAPKKK families were induced either under all water conditions, or specific to a single water condition (Figure S10A, B). As members of the MAPK signaling cascade are known for their roles in in stress response and development [38, 39], our results suggested that distinct members of these families could form similar signaling cascades depending on the expression at specific developmental phases and under different water conditions. In addition, examples of gene families that were induced specifically at a developmental phase include three DUF (domain of unknown function) families with elevated expression specific to a phase and/or water condition (Figure S10C), suggesting their potential roles during flower development. In total, 6056 genes were up-regulated and 4161 were down-regulated (Figure S7E) when comparing the three flower development phases, with a combined total of 8694 DVGs (Figure S7F, File S3, 4).

**Fig. 4:**
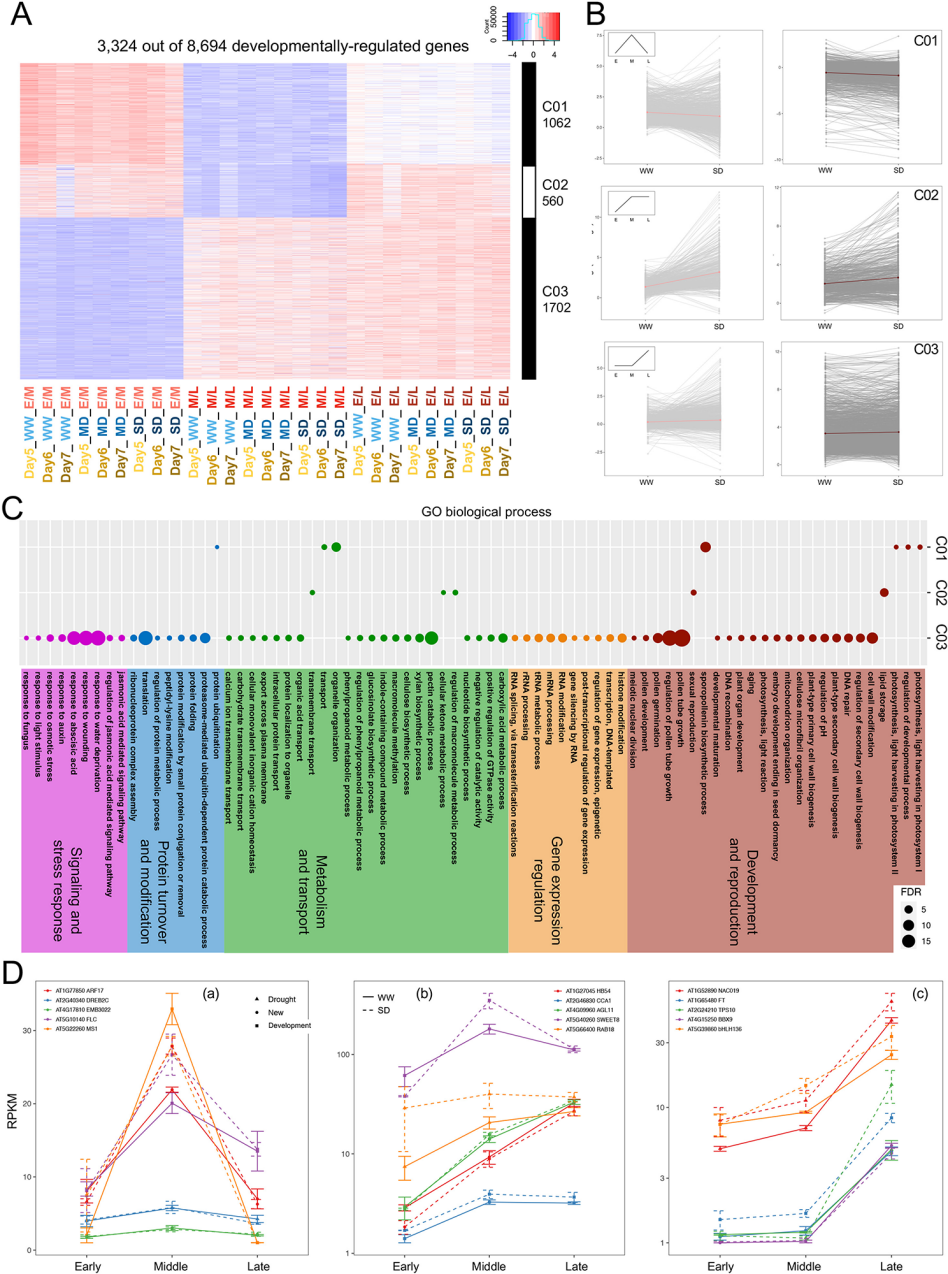
Developmental phase-dependent transcriptome reprogramming under drought of representative DVG clusters. **A**. Expression dynamics under the 3 different water conditions and across the 3 days of 3 representative clusters (C01-C03) including 3324 DVGs. Red indicates up-regulation, blue indicated down-regulation and white indicates no change. **B**. The fold change of all genes (grey) in the corresponding clusters under WW and SD on Day 7 from 2 developmental phase comparisons. The light red line represents the average fold change from E/M of all genes in each cluster; the dark red line represents the average fold change from E/L of all genes in each cluster. The mini graph within each box represents the general trend of gene expression throughout the 3 developmental phases. **C**. GO enrichment based on genes from the corresponding clusters. Colors of the dots refer to different general biological processes, and sizes of the dots refer to the level of enrichment (-log_10_FDR). **D**. The expression level (average RPKM values between the 3 days under WW or SD across the 3 developmental phases) of representative genes from the corresponding clusters. (a): genes from C01; (b): genes from C02; (c): genes from C03. Square indicates that the gene has a previously reported function in development, triangle indicates that the gene has a previously reported function in drought response, and circle indicates that the gene has no published functional study. Each color represents a different gene, though the same color in different graphs does not refer to the same gene. Solid line represents WW, dashed line represents SD

To identify shared patterns of differential gene expression, K-means clustering analysis was performed with the 8694 DVGs using their fold-change over different developmental phases; nine clusters were generated (K = 9) and the expression patterns of DVGs in each cluster with differential expression under three water conditions and across the three days are shown (Fig. 4A for C01-03; Figure S11A for others, File S4). The average expression pattern of all genes from each cluster was also plotted in the form of log_2_ fold change (Fig. 4B for the first three clusters; see Figure S11B for others), and the general trend of RPKM values across the three developmental phases is shown in the upper left corner of each plot. For the RPKM values, C01 (1062 genes) showed a peak at the middle phase (Fig. 4B: C01), C02 (560 genes) was high at the middle phase and maintained for the late phase (Fig. 4B: C02), and C03 (1702 genes) was induced at the late phase in comparison to both the early and middle phases (Fig. 4B: C03). The average expression fold change under SD showed a slight difference from that under WW in C01-03 (Fig. 4B). It is possible that drought resulted in a reduction in the fold change of floral gene expression among developmental phases; alternatively, the expression changes might be delayed till the water availability was improved.

GO enrichment analysis of individual clusters (Fig. 4C) showed that C01 DVGs with peak expression at middle stage were enriched in sporopollenin biosynthesis and organelle organization. C02 with DVGs that were induced at the middle and late phases was enriched in lipid storage, metabolism, and substance transport. For C03 with DVGs that were induced at the late phase, the enriched GO terms include pollen tube growth, pectin catabolic process, translation, and response to ABA and water deprivation. Highly enriched GO terms from other clusters include DNA replication, male meiosis, RNA modification, microtubule-based movement, and response to JA (Figure S11C), suggesting that proper expression of genes for these processes might be important for reproductive development under drought stress.

Multiple previously reported genes were also found in each cluster (Fig. 4D, Figure S11D). C01 (Fig. 4Da) contains *MS1*, which is required for anther gene expression for early pollen development [40], consistent with a high expression in the middle phase under all water conditions. On the other hand, another gene in C01, *FLC*, is a repressor of flowering [41] and is expressed in male meiotic cells [42], yet its role during flower development has not been reported. Our results showed that *FLC* is expressed at all three developmental phases and relatively high during the middle phase, with some increase under drought conditions. C02 (Fig. 4Db) included *SWEET8*, which is known for pollen wall formation [30] and was dramatically highly induced at the middle phase under drought conditions. Among the genes in Cluster 03 (Fig. 4Dc) was *NAC019*, which was shown to be important for normal flower development under drought stress [12]. Another C03 gene was *FT*, which is a positive regulator of flowering [31], which is consistent with previous finding [11], suggesting a role in late phase flower development per se.

We also examined expression of genes for annotated TFs among all DVGs and in specific clusters. Out of the 8694 DVGs, 692 (8.0%) were annotated to be TFs, representing most TF families with exceptions such as CAMTA and Whirly (Figure S12A). TF families that were significantly enriched for flower development include B3, bZIP, GRF and M-type MADS-box families. Unlike the MIKC MADS-box genes with well-known functions in flower development, the M-type MADS-box genes were identified by sequence comparison and generally lack genetically determined functions, but their expression patterns suggest possible roles in reproductive processes [32, 33]. Our results support their possible functions in floral development (Figure S11D) and further studies of these TF genes are needed to test their roles in flower development and stress response. GRNs (gene regulation networks) were constructed based on the TFs from each cluster; 84 of 90 TFs from C01 formed a closely interrelated complex GRN, with *EMB3022*, a C2H2 family member at the center (Fig. 4Da, Figure S12B). *EMB3022* was reported to be involved in root hair development [43], but its function during flower development has not been examined. Similar analyses also generated GRNs for Cluster 02 (Figure S12C) and 03 (Figure S12D) with, respectively, 20 (of 28) and 52 (of 94) TF genes. The central nodes of the C02 GRN are *HB54* and *CCA1* (Fig. 4Db, Figure S12C); although *HB54* has no function reported, *CCA1* is a crucial regulator the circadian clock [44]. For C03, the central nodes of the GRN are *BBX9* and *bHLH136* (Fig. 4Dc, Figure S12D); *bHLH136* is important for cell elongation in the hypocotyl downstream of multiple hormonal pathways [45]. These GRNs suggest that the genes occupying central nodes might have important roles in reproductive development. Comparison of DVGs under different water conditions indicated that the gene identities and TF-GRNs were quite different, strongly suggesting that drought substantially affected transcriptomic remodeling during reproductive development. Moreover, the comparison of DVGs between the three days also revealed different subsets of DVGs that are either specific or shared (Figure S13).

### Largely distinct sets of age-dependent genes under different water conditions and in different developmental phases

*Arabidopsis* plants experience major transitions during its lifecycle [46–49], including the transition from the juvenile to adult phase during vegetative development [50–52], the flowering transition from vegetative to reproductive development [50, 51, 53, 54], and senescence in late reproductive phase [55–58]. The phases marked by these transitions span weeks and are accompanied by major morphological changes and require crucial regulators. For example, microRNAs miR156C and miR172A and their target genes play key roles in the juvenile to adult transition and several pathways are known to regulate the flowering transition by integrating both internal and external signals [47, 59, 60]. However, possible effects of the age of plant on reproductive development are not clear, especially not the differences of floral transcriptomic program over the span of a few days during the reproductive phase. Our sampling of floral tissues at Day0, Day5, Day6 and Day7, initially for comparison with drought treated samples of the same number of days after soil water reduction, offered an opportunity to begin an investigation of age effects on reproductive gene expression profiles, as the plants were growing older each day. Thus, we defined age-related genes (ARGs) as those that show differential expression among the days of the plants (for the same water condition and the same floral developmental phase) using log2 (fold-change of expression level between Day0/Day5, Day0/Day6, Day0/Day7, Day5/Day6, Day6/Day7, or Day5/Day7) ≥ 1 or ≤ -1 and p-value < 0.05 as cutoff. As the sampling was from plants that had recently entered into the reproductive phase (since the first mature flower was formed), one possibility is that the increase in gene expression reflects some aspect of enhanced robustness of the reproductive program. Alternatively, some genes might have increased expression in support of physiological and biochemical changes not obvious from morphological characteristics.

**Fig. 5:**
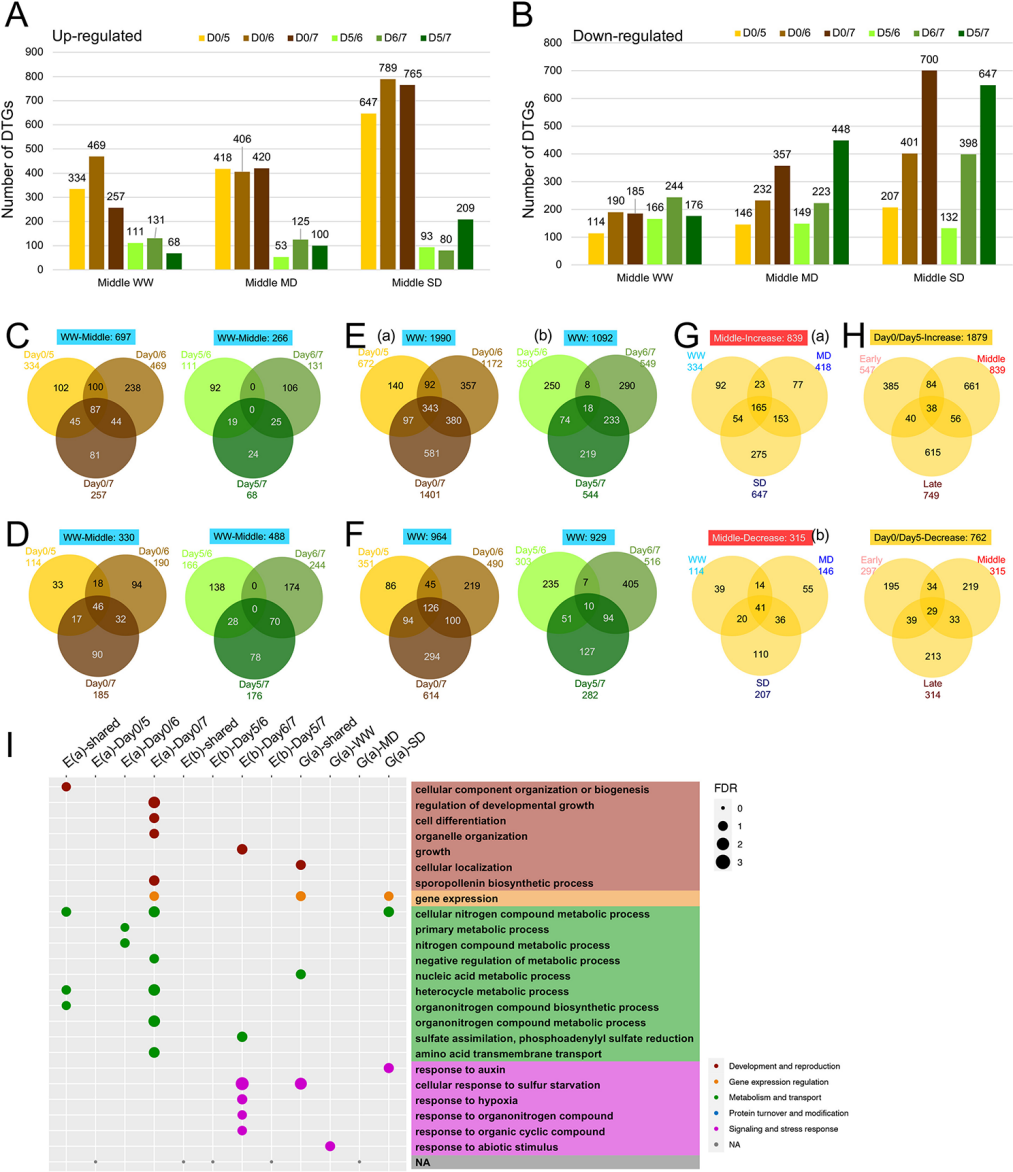
Aging induces transcriptomic changes under different drought stress at different developmental phases. **A-B**. Total number of aging-related genes (ARGs) in Day 0/5, Day 0/6, Day 0/7, Day 5/6, Day 6/7 and Day 5/7 under each water condition during middle phase. **A**: up-regulated genes; **B**: down-regulated genes. **C-D**. Comparison between the ARGs from the 6 age comparisons under WW at the middle phase. **C**: up-regulated genes; **D**: down-regulated genes. Left: comparison between D0/5, D0/6, D0/7; right: comparison between D5/6, D6/7 and D5/7. **E-F**. Comparison between the ARGs from the 6 age comparisons under WW with all 3 developmental phases. **E**: up-regulated genes; **F**: down-regulated genes. Left: comparison between D0/5, D0/6, D0/7; right: comparison between D5/6, D6/7 and D5/7. **G**. Comparison between the ARGs from Day 0/5 comparison under the 3 different water conditions at middle phase. Top (a): up-regulated genes; bottom (b): down-regulated genes. **H**. Comparison between the ARGs from Day 0/5 comparison under the 3 different water conditions with all 3 developmental phase. Top: up-regulated genes; bottom: down-regulated genes. **I**. GO enrichment of specific subsets of ARGs from E(a), (b) and G(a). Colors of the dots refer to different general biological processes, and sizes of the dots refer to the level of enrichment (-log_10_FDR). Yellow represents Day 0/5 (expression level in Day 5 compared to Day 0), brown represents Day 0/6 (expression level in Day 6 compared to Day 0), asparagus represents Day 0/7 (expression level in Day 7 compared to Day 0), dark yellow represents Day 5/6 (expression level in Day 6 compared to Day 5), dark brown represents Day 6/7 (expression level in Day 7 compared to Day 6), dark asparagus represents Day 5/7 (expression level in Day 7 compared to Day 5)

The results showed that the older the plants were, generally the more ARGs were detected, ranging from 700 to over 1000 ARGs in a single comparison (Fig. 5A, B, Figure S14A, B). Surprisingly, approximately 100–200 genes were differentially expressed over a 24-hour period from Day5 to Day6 or from Day6 to Day7, suggesting some molecular differences between phenotypically similar flowers (Fig. 5A-F, Figure S15). It is possible that for a plant like *Arabidopsis* with a very short generation time of several weeks, even one day of age difference can have corresponding molecular changes. Also, although flowers on Day5, Day6 and Day7 appear very similar morphologically and have highly similar biological processes for the flower developmental program, the various sets of ARGs among different days shared only ~10–30% of induced genes (Fig. 5C-F, Figure S14C, D). These results suggest that there are transcriptomic differences as the plants became slightly older.

Up-regulated ARGs of the comparisons of one of Day5, 6, or 7 with Day0 under WW conditions from all three developmental phases (Fig. 5Ea) were enriched in cellular component organization and nitrogen metabolic pathways (Fig. 5I: column “E(a)-shared”), consistent with the idea that plant age might have affected physiological and biochemical aspects of flowers. In addition, ARGs for Day6 relative to Day0 were enriched for metabolism (Fig. 5I: column “E(a)-Day0/6”) and ARGs for Day7 vs. Day0 were enriched for cell differentiation, developmental growth, gene expression, nitrogen and amino acid metabolism (Fig. 5I: column “E(a)-Day0/7”). The ARGs for Day7 in comparison with Day6 were enriched for growth, sulfate assimilation, and response to different compounds and stresses (Fig. 5I: column “E(b)-Day6/7”), suggesting enhancement of cellular processes during reproductive development. Further, the ARGs at the same developmental stage from the same age comparison shared approximately 1/5–1/3 ARGs among different water conditions (for Day5 compared with Day0 see Fig. 5G, the middle phase. See Figure S16 for additional comparisons). GO analyses revealed (Fig. 5I) that up-regulated ARGs on Day5 compared with Day0 under all water conditions were enriched in cellular localization, gene expression, nucleic acid metabolism and response to sulfur starvation (Fig. 5I: column “G(a)-shared”), whereas the ARGs specific for WW enriched in response to abiotic stimulus (Fig. 5I: column “G(a)-WW”). In addition, SD-specific ARGs were enriched in gene expression regulation, nitrogen metabolism, and response to auxin (Fig. 5I: column “G(a)-SD”), implying possible role of nitrogenous compounds during drought response. Moreover, up-regulated ARGs identified on Day7 were almost doubled compared to Day5 (Fig. 5H, Figure S17A, C); most enriched GO terms on Day5 also were enriched among ARGs on Day6 and Day7. Several processes, like DNA repair, pollen tube growth, intracellular transport, macromolecule methylation, translation, and response to water deprivation, showed higher enrichment level in older plants; additional processes were enriched specifically for older plants, such as leaf senescence, pollen sperm cell differentiation, gene silencing by RNA, mRNA metabolism, nucleotide metabolism, peptidyl-lysine modification, and response to ABA and stresses (Figure S17E). The enriched terms for cellular components include nuclear complexes, chloroplast stroma, extracellular region, and plasma membrane dynamics, and others (Figure S17F), suggesting a greater need for the related gene activities as plants progress through reproductive development. Overall, 4440 up-regulated and 3855 down-regulated genes (Figure S17B, C) were related to plant age differences, totaling 6491 ARGs (Figure S17D, File S5, 6, 7).

**Fig. 6:**
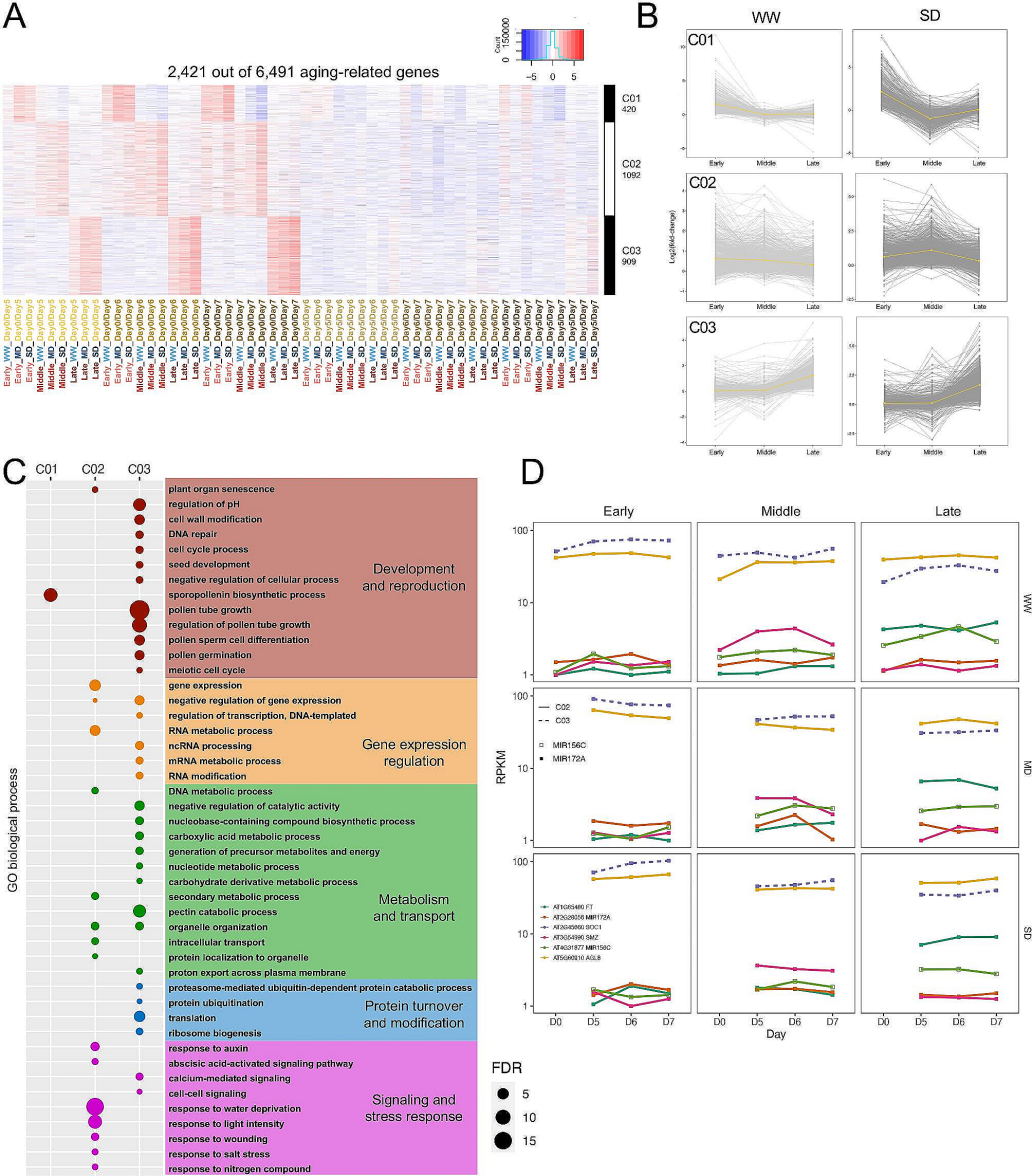
Transcriptome reprogramming during reproductive development under drought of representative ARG clusters. **A**. Expression dynamics under the 3 different water conditions and across the 3 developmental phases of 3 representative clusters (C01-C03) including 2421 ARGs. Red indicates up-regulation, blue indicated down-regulation and white indicates no change. **B**. The fold change of all genes (grey) in the corresponding clusters under WW and SD on Day 7 from age comparisons. The yellow line represents the average fold change from Day0/7 of all genes in each cluster. **C**. GO enrichment based on genes from the corresponding clusters. Colors of the dots refer to different general biological processes, and sizes of the dots refer to the level of enrichment (-log_10_FDR). **D**. The expression level of previously reported aging-related genes from the corresponding clusters. Each color represents a different gene, though the same color in different graphs does not refer to the same gene. Solid line indicates the gene belongs to cluster 02, and dashed line indicates the gene belongs to cluster 03. Thin line represents Day 0/5, regular line represents Day 0/6, and thick line represents Day 0/7. Solid square indicates the gene has a previously reported function related to miR172 and open square indicates the gene has a previously reported function related to miR156

K-means clustering analysis of the 6491 ARGs with their fold-change over different ages of the plant resulted in 10 clusters (K = 10) (Fig. 6A, Figure S18A, File S7). The average expression pattern of log_2_ fold change of all genes from each cluster (Fig. 6B, Figure S18B) showed that each cluster displayed different expression profile: C01- C03 (420, 1092, 909 genes, respectively; Fig. 6A). The expression fold changes on Day7 vs. Day0 in Fig. 6B show ARGs that were induced to a higher degree in early, middle, or late phases, respectively [(Fig. 6B: C01), middle (Fig. 6B: C02) and late (Fig. 6B: C03)], suggesting some role of these genes for (physiological) changes among the days at different floral developmental phases. GO enrichment analysis showed that ARGs from C01 were enriched solely in sporopollenin biosynthesis. C02 showed enrichment in gene expression, RNA metabolism, and response to stresses. C03 was enriched in developmental processes especially pollen tube growth, multiple types of RNA modification and metabolism, primary and secondary metabolism, and surprisingly translation (Fig. 6C). Enriched GO terms from other clusters include lipid storage, photosynthesis, secondary metabolism, response to stimulus, and defense response (Figure S18C). Known age-related genes and genes with potentially new functions that showed the largest degree of induction over the days have been examined in each cluster. C01 (Figure S18D: C01) included ARGs that were specific to the early phase; for example, *MS2* encodes a fatty acid reductase known for pollen wall formation [61] and another for a protein containing an F-box. C02 (Fig. 6D, Figure S18D: C02) contained genes that were induced by age specifically in middle-stage flowers, such as genes for miR156C and miR172A [59], *FT* [31] and *FRUITFULL* (*AGL8*) [62], and also *HIS1-3*,which encodes a variant of link histone responsive to salt stress [63], implying a possible enhancement of chromosome organization in the middle phase flowers when plants get older. C03 (Fig. 6D, Figure S18D: C03) consisted of genes that showed induction by age only in late-stage flowers and included a known gene for promote flowering, *SOC1* (*AGL20*) [64] and *SWEET9* encoding a sucrose transporter [65]. Together these results indicated the increasing activity of different functions at different floral developmental phases as plants progress further into the reproductive phase.

Age-related TFs and GRNs during flower development (after the flowering transition) were not reported previously. Here, we identified that 495 of 6491 ARGs (7.63%) are annotated TFs, representing 46 of 50 TF families (Figure S19A). Among these, large TF families such as bHLH, MYB, NAC, and especially members from the ERF family of the AP2/B3 superfamily and the M-type MADS-box family were significantly enriched among ARGs. Specifically, 21 of 34 TFs from C01 (early-flower ARGs) formed a GRN centering at two NAC factors without a previously reported function (Figure S19B), implying their increased roles in early flower development as plants advanced in the reproductive phase. On the other hand, 74 of 78 TFs from C02 (middle-flower ARGs) formed a tightly interrelated GRN that features three TFs as the central nodes. One of these three is *C3H47* and is known for response to salt stress [66], but no age related study has been reported (Figure S19C). The GRN of C03 includes 30 of 34 TFs, with *BBX9* as the central node (Figure S19D), suggesting a potential role in sustaining flower development as the plant ages. Together with the comparisons of ARGs under the same water condition between different floral phases (Figure S20), morphologically similar flowers at the same developmental phase from three consecutive days had considerable difference at the transcriptome level, regarding the individual gene identity, gene expression level and even detailed GO terms. These differences might reflect changes in the physiology and biochemistry of the flower, possibly in part to promote a robust and sustained reproductive program during the progress from a plant that just started reproductive development to a plant with a more mature age.

### Overall comparison of *Arabidopsis* floral transcriptomes under different water availability

**Fig. 7:**
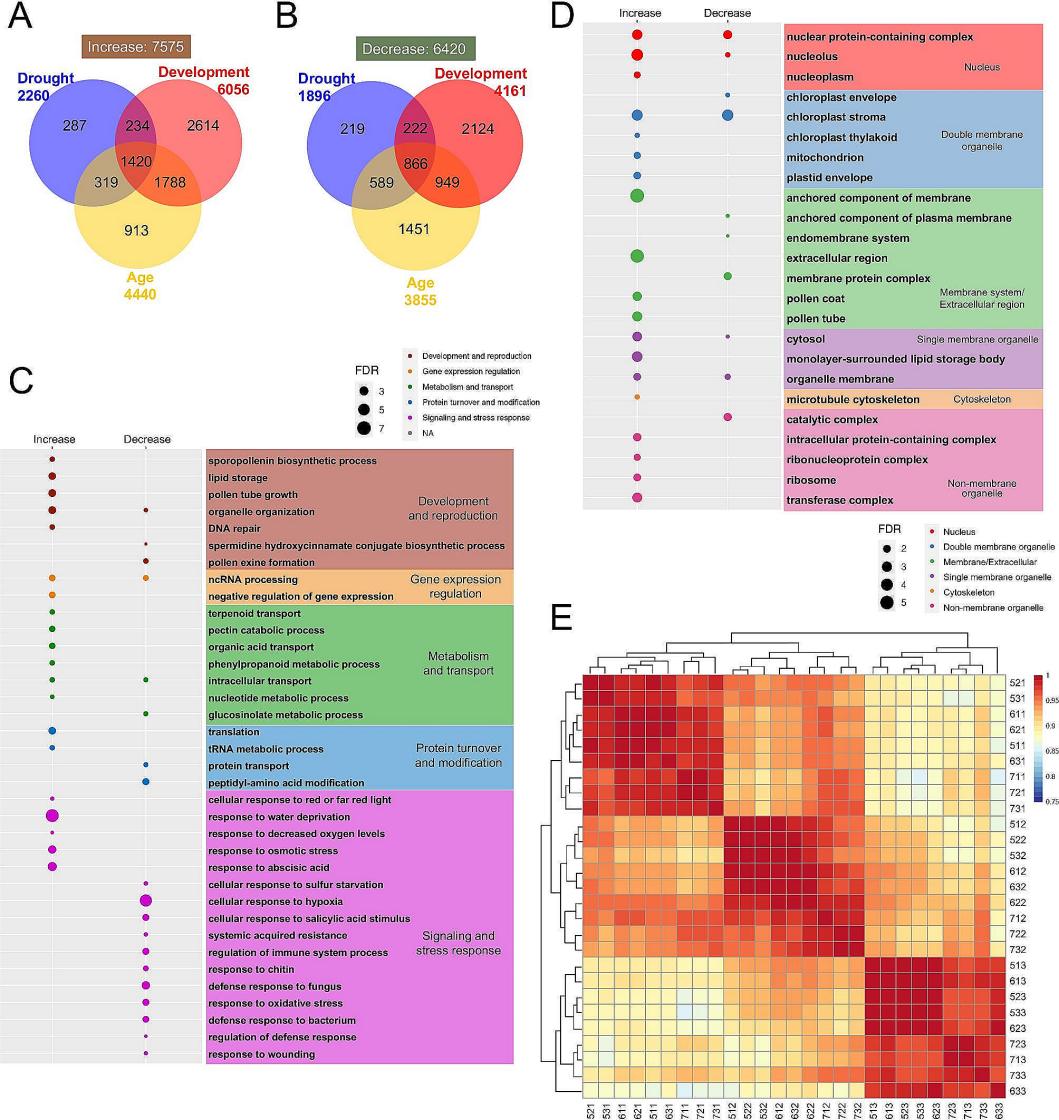
Transcriptome profiling of Arabidopsis reproductive development under drought stress over a 3-day period. **A-B**. Comparison between drought-responsive, developmentally-regulated and aging-related genes. **A**: up-regulated genes; **B**: down-regulated genes. Blue refers to DTGs; red refers to DVGs; yellow refers to ARGs. **C**. GO enrichment of biological processes of the commonly up- or down-regulated genes from all three sections. Colors of the dots refer to different general biological processes, and sizes of the dots refer to the level of enrichment (-log_10_FDR). **D**. GO enrichment of cellular components of the commonly up- or down-regulated genes from all three sections. Colors of the dots refer to different general cellular components, and sizes of the dots refer to the level of enrichment (-log_10_FDR). **E**. Hierarchical clustering analysis of the 30 samples (water availability, developmental phases and days)

We further examined the transcriptomic changes in developing *Arabidopsis* flowers combining the three treatments: water availability, developmental phases, and days during early portion of reproduction (Fig. 7). Among 7575 genes up-regulated (drought vs. WW; later vs. earlier; older vs. younger), 1420 (18.7%) were shared by all three treatments, while 866 (13.5%) were shared among 6420 down-regulated genes (Fig. 7A, B), suggesting that these genes might underlie functional interactions among the three conditions. In addition, DVGs and ARGs had greater overlap, suggesting that floral developmental phases might be more related to maturation or robustness during progression in reproduction, whereas both processes might be more distinct from drought responses. The up-regulated genes shared by all three treatments showed enrichment in functional categories for pollen-related terms, terms for secondary metabolism and metabolite transport, and various stress responses, whereas enriched GO categories for commonly down-regulated genes are predominantly those for defense against biotic stimulus (Fig. 7C). For cellular components and processes, different subsets of genes for categories involved in nuclear components, chloroplast stroma and cellular transport were regulated in opposite manners, whereas translation related processes and several others were strengthened (Fig. 7C, D). The enriched GO categories suggest enhanced activities to promote multiple aspects during flower development, while saving energy by reducing some less critical processes. We also constructed GRNs based on the 97 and 86 commonly up- or down-regulated TFs from all three treatments. Forty-four up-regulated TFs formed a well-connected GRN, featuring *NAC006* and a bHLH TF as central nodes (Figure S21A), whereas thirty down-regulated TFs formed a network with *MYB82* at the center (Figure S21B). *MYB82* was previously shown to be involved in trichome development [67]; our results suggesting it might also function in flower development and drought response. These putative regulatory factors are excellent candidates for genetic studies of in vivo functions.

We also compared the transcriptomes from three treatments using 2D hierarchical clustering (Fig. 7E) and principal component analysis (PCA) (Figure S21C-E, File S8). The 2D hierarchical clustering analysis suggests that the differences among flower developmental phases were greater than the differences due to the other two treatments, whereas samples for different water conditions were more distinctive than those from different days (ages) (Fig. 7E). In addition, PCA identified the first three components (PC1, PC2, PC3) that explain 45.78%, 28.31%, and 14.37% of the variance, respectively, with PC1 corresponded very well with the flower developmental phase (Figure S21C, D). However, there was no clear correspondence between PC2/PC3 with water condition or days (Figure S21E), suggesting that differential gene expression of these two treatments might have contributed to both PC2 and PC3. Together, the floral developmental phases had the largest impact on transcriptomic differences, and both the water conditions and the age of the plants also had clear effects on the transcriptomes.

## Discussion

### Comparison with previous flower transcriptomes under drought

Previous studies have reported thousands of drought-responsive genes in the whole inflorescence under SD or MD using microarray [10, 11] (Figure S22A). Direct comparison between our DTGs to previous transcriptomic datasets (Figure S22B-C) revealed that a quarter to a third of the DVGs here were also identified in previous studies, although the detailed design for drought treatment was different with daily addition of small amounts of water to maintain a nearly constant soil moisture. These shared DTGs might represent a core set of drought responsive genes that are involved in acclimation to various drought conditions. Thousands of newly identified DTGs (70–80%) from this study might be due to a few factors: (1) the use of RNA-seq allowed a chance to detect expression of any annotated genes, in contrast to a subset of genes represented by the microarrays; (2) the separately isolated RNAs from early, middle and late floral developmental phases likely enhance the representation of genes specific to early and middle phases, as these floral buds were a tiny part of the whole inflorescence; (3) the drought treatment schemes and the general growth environment were different between this and the previous studies. The second possibility is further supported by the finding that the DTGs from specific developmental phases were mostly (60–70%) not overlapping with previous drought-affected floral genes (Figure S22D). Our results provided additional information regarding the floral developmental phase(s) for these gene activities.

We also compared the previous drought transcriptomes with our DVGs to investigate the potential interaction between drought response and reproductive development. Surprisingly, more overlapping DVGs (1604 and 874 up- and down-regulated genes, respectively) with previous studies were identified than the overlapping DTGs (661 and 425 up- and down-regulated genes) (Figure S22E-G). This could partly be due to the fact that there were more differentially expressed DVGs than DTGs; alternatively, the overlap between the DVGs here and previously identified drought-affected genes might imply that the previous longer drought treatment (up to 10 days) might have affected more genes related to regulation by floral developmental phases. The DVGs that overlapped with the previously identified drought-affected genes made up a fifth to a quarter of all DVGs we found here, suggesting their possible roles in stress response during flower development, though experimental evidence is needed to further support this idea. Moreover, we compared our DVGs with previously known floral genes from RNA-seq and ChIP-seq experiments [68] (Figure S22H), and found that about three quarters of our DVGs were previously identified, and the one quarter (> 2,000) of the newly identified DVGs were specific for one of the comparisons among the floral phases, suggesting their specific roles during a short period during flower development, or for maintaining flower development under adverse environments.

### Overlaps between DTGs, DVGs and ARGs suggest complex interactions between drought response and flower development during plant maturation

The transcriptomic analyses provide differential gene expression for growth under three water conditions, three floral phases, and different days during the progression in reproductive development. These results also offer an opportunity to examine possible interactions among the affected genes due to the three types of treatments. Thus, we examined DVGs and DTGs affected in one sample, in comparison with either a different phase, or with a different water condition; for example, DVGs up-regulated in the middle phase under SD when compared with the early phase and DTGs in the middle phase under SD when compared with the middle phase under WW condition (Figure S23). These comparisons revealed that the overlapping genes accounted for one third to two thirds of DTGs and 10–20% of DVGs, greater than the overlaps expected from chance alone. For example, we compared the 2154 up-regulated DVGs induced in the middle phase than the early phase under SD from all 3 days with the 705 up-regulated DTGs induced under SD at the middle phase from all 3 days, and found 409 overlapping genes between the mentioned DVGs and DTGs. The overlapping set of 409 is ~58% of the 705 DTGs and 19% of the 2154 DVGs (Figure S23Ca), suggesting that a considerable number of genes are involved in both stress response and reproductive development. GO enrichment analysis of these 409 up-regulated genes showed that they were enriched in spermidine biosynthesis and lipid storage (Figure S23E). One possibility is that lipid storage might be both (1) important for reproductive development either as energy reservoir or providing precursors for pollen wall materials and (2) supporting protection of certain developing flowers under drought stress. The overlapping genes of DVGs genes at late stage and DTGs under drought stress were enriched in gene expression regulation, metabolic processes, and response to drought stress or phytochromes (Figure S23E), indicating their importance in late-stage flowers under drought stress. These genes are great candidates for genetic studies regarding flower development under drought stress.

Similarly, we examined ARGs induced under drought conditions and the DTGs from the same day to estimate their overlap (Figure S24). We found that 50–70% ARGs with increased expression over the days in our treatment under drought conditions were also induced (DTGs) under drought (SD vs. WW) on a specific day, whereas the remaining 30–50% ARGs did not overlap with DTGs. For instance, 672 ARGs were up-regulated at Day5 days under WW, and 400 DTGs were up-regulated by SD on Day5 (Figure S24Aa). In addition, the 1378 ARGs up-regulated at Day5 (vs. Day0) under SD contained 48% genes from the other two comparisons, while the other half of ARGs under SD were induced under drought but not the other two comparisons (Figure S24Aa), suggesting that the age-effect on gene expression in plants under SD is not a simple addition to the age-effect of plants under WW and the drought effects. Therefore, the effect of drought stress during plant aging is complex and possibly reflects a balance between stress response and growth and development. GO enrichment of DTGs and ARGs showed that some common terms shared between genes from different overlapping subsets are lipid storage, pollen tube growth, secondary metabolism, translation, response to ABA and water deprivation (Figure S24E), but GO terms enriched for ARGs under drought stress, again, is not a replica of either the terms enriched for ARGs under WW or DTGs, implying complex effects of aging and drought stress on floral transcriptomes.

### Transcriptomic analyses reveal novel age-related changes and suggest greater differences among the floral phases than other treatments

The transcriptomic datasets of developmental flowers for three floral phases, from plants under different water conditions, and at different days during reproduction provide a window into the molecular characteristics of these floral buds, separate from the morphological descriptions. Specifically, previous molecular genetic studies of plant aging and phase transition have largely focused on phase transitions [47], but changes at the transcriptome level related to small difference in age after the flowering transition have not been examined. Morphologically, the floral buds sampled on Day0, Day5, Day6, and Day7 are very similar, especially when they are from plants under the same water condition. However, our results showed that even a 24-hour progression in the reproductive phase corresponded to changes in expression level for hundreds of genes (Fig. 5, Figure S15); moreover, the genes with increases in expression from Day5 to Day6 do not overlap much with genes with increased expression from Day6 to Day7, suggesting that as the plant proceeded into the reproductive developmental phase more and more, physiological and biochemical changes continue with different genes showing expression increases.

The complex effects of water condition, flower development, and plant aging observed at the transcriptomic level led us to ask which treatment caused the most changes in gene expression. The results in differentially expressed genes already suggested that floral buds in different phases (DVGs) have more distinct transcriptomes than those of the same phase but from plants under different water conditions (DTGs) or different days (ARGs) (Fig. 7A, B, Figure S8, Figure S23). In addition, the hierarchical clustering showed that transcriptomes of the floral phases were separated first, whereas the PC1 with ~45% of the variance corresponded well with the three phases (Fig. 7E, Figure S21C-E). It is likely that, even under non-lethal drought conditions, the overall transcriptomic program is directed towards the progression of flower development; thus the same phase is more similar over different water conditions. Morphologically at the organ and cell levels, the three floral phases are clearly different and with distinct cellular processes, more so than the same phase from plants under different water conditions or days; therefore, the transcriptomic differences being more obvious for the developmental phases are not surprising. Nevertheless, the molecular differences likely uncover features of three phases that are related to physiological and biochemical aspects but not easily seen anatomically. Further analyses are needed to understand these differential gene functions and could potentially provide new insights into flower development, especially under drought.

Our study provides a thorough transcriptomic characterization of *Arabidopsis* flower at different floral developmental phases under three water conditions and on different days over a 3-day period. Our results suggest that flowers in different phases respond to drought stress differently, that drought stress modulates floral transcriptomes at different phases differently, and that flowers at different ages as the plants progress through reproduction exhibit distinct molecular features. Our transcriptome datasets and analyses provide a useful resource for future genetic studies of development and drought responses.

## Methods

### Plant material treatment and collection

*Arabidopsis thaliana* ecotype Columbia (Col-0) seeds were directly sown into pots with soil mixture of Pro-Mix soil (Premier Tech) and Turface Profile Greens Grade (Profile Products LLC) in 3:2 ratio by volume. Six random pots with soil were baked in 60 ˚C oven for 2 days, so that the dry soil weight was measured as 120 g and used to determine the water weight for different water conditions. The initial pot weight was adjusted to 228 g by adding 108 g water into the pot and this was defined as 90% soil water content (SWC). Seeds were stratified after sowing in a 4 ˚C dark room for three days, then transferred into a Conviron growth chamber (Conviron Inc.) at 22 ˚C, 16 h/ 8 h day/night photoperiod, ~300 µmol m^− 2^ s^− 1^ photon flux, 50% humidity. Until just after bolting (the main stem was about 1 cm high, day 28 after sowing), half of the pots were subjected to different degrees of drought stress.

For the drought treatment, five water conditions (SWC as: WW = 80–90%, ½ MD = 60–70%, MD = 50–60%, ½ SD = 40–50%, SD = 30–35%) were included. Pots were arranged according to a randomized design and the positions were changed daily. Drought treatment was conducted by withholding water (defined as Day0 for drought treatment, principal growth stage 5.80–5.90 [69]), so that the SWC of the treated pots decreased gradually. When the SWC of each group reached the designated range of SWC, watering was resumed by adding a small amount of water to maintain the SWC within the range. After about five days, the SWC of the lowest water condition group reached ~35% (defined as Day5 for drought treatment), and all drought treated groups had their designated water conditions. Each group was maintained at their designated water conditions for additional three days by adding a small amount of water daily.

For tissue sampling, unopened floral buds were collected at Day0, Day5, Day6, and Day7 from plants of each of three water conditions (SWC as: WW = 80–90%, MD = 50–60%, and SD = 30–35%). Based on flower and anther development stages [14, 70], we divided each inflorescence into 3 parts: early, middle, and late phases. The early phase flowers contain the smallest floral buds at flower development stages 1–8, before male meiosis. The middle phase consist of the next larger 2–3 flower buds near flower development stages 8–10, include pollen mother cells just before and during male meiosis, and likely tetrads with newly formed microspores. The late phase with 9–11 oldest floral buds, approximately at flower development stages 10–12, which include developing and mature pollen grains. The phase identification and separation were confirmed under a dissection microscope (Nikon). A total of 30 treatments/tissues were obtained. Three biological replicates were collected for each treatment/tissue; each replicate consisted of floral bud tissues from six plants. Samples were frozen in liquid nitrogen immediately after harvesting and dissecting, and stored in -80 ˚C.

### Phenotypic characterization of reproductive tissues under drought conditions

Siliques and seeds were counted as an estimate of reproductive yield for two individual plants for each of five water conditions (see above), after the plants had stopped growing. For each plant, the following information was collected: number of branches, the position of each silique (from bottom to top, separating the main stem from branches), length of each silique, number of seeds of each silique, the weight of all seeds produced. Then the total silique number per plant and total seed number per plant were calculated, as well as the average seed number per silique and average silique length of the two plants under the same water condition. Bar graphs and line graphs were generated using ggplot2 (version 3.3.6) in R software (version 4.2.0).

### RNA isolation, RNA-sequencing and data analysis

RNAs were isolated using NucleoSpin Plant RNA kit (MACHEREY-NAGEL). The quality of RNA samples was assessed using Agilent 2100 Bioanalyzer, and an average RNA integrity number (RIN) of 89 samples (#36 was lost) was ~9.34 (median was 9.3, 25% percentile was 9.1, and 75% percentile was 9.7). The library was then generated with the 3’ mRNA-Seq Library Prep Kit (Lexogen) which produces library inserts from the 3’ end of transcripts. RNA-sequencing was conducted using Illumina NextSeq 2000 High Output 75 nt single-end read sequencing and 2 individual runs were performed. RNA-seq reads were assembled and differential expression analyses were conducted using Ubuntu (version 22.04) on the VMware Fusion platform (version 12.0.0). The reads assembly used the *Arabidopsis* genome from the TAIR10 release as a reference using hisat2 (version 2.2.1). Across all 89 samples, the average number of reads per sample was 15.107 million reads (the lowest read number was ~11.736 million), and on average, 85.75% of reads were mapped for exactly one time (File S9). The differential gene expression was calculated using Cufflinks (version 2.2.1) with |log_2_ (fold-change of expression level between 2 treatments/ tissues) | ≥ 1 and *p*-value < 0.05 as cutoff, and read counts (RPKM) were obtained through HTSeq (version 2.0.2), and the reads per kilobase million (RPKM) value for each gene was normalized against total read count for each sample as well as the gene length from TAIR10.

Venn diagrams were generated according to Venny (version 2.1.0) (https://bioinfogp.cnb.csic.es/tools/venny/). GO enrichment analyses were conducted using Gene Ontology (http://geneontology.org) and the GO terms with FDR < 0.05 were considered as enriched.

Subsequent analyses and plotting were conducted with the R software (version 4.2.0) unless otherwise specified. Bar graphs were generated using Microsoft Office 365 Excel or ggplot2 (version 3.3.6) in the R software. Line graphs were generated using ggplot2 (version 3.3.6). Clustering analyses were conducted using factoextra (version 1.0.7), with the fold change for each comparison as the input. Heatmaps were generated using gplots (version 3.1.3) with the fold change for the corresponding analyses and the values scaled within each dataset, and the plots were generated using ggplot2 (version 3.3.6).

The GRNs were constructed using GRENITS (version 1.48.0) (probability threshold > 0.6, p-value < 0.001) and visualized using Cytoscape (version 3.9.1) as previously described (Morrissey et al., 2011; Wu et al., 2021). The package is based on dynamic Bayesian networks (Morrissey et al., 2011); the number of edges of the node is reflected by the color of the node; the centrality of the node is reflected by the size of the node; the probability of the interaction (posterior link probability) is reflected by the width of the edge. The GRNs of the clusters from each comparison (Figure S5, 12, 19) used the fold change of the corresponding comparisons as the input; the GRNs for the overall comparison (Figure S21) used the RPKM across all the 90 treatments as the input.

The correlation between replications was calculated with R and plotted by pheatmap (version 1.0.12). The hierarchical clustering analysis was conducted using pheatmap (version 1.0.12), and the principal component analysis (PCA) was conducted using R and plotted by plotly (version 4.10.0). The RPKM values were used for the above-mentioned analyses.

### Electronic supplementary material

Below is the link to the electronic supplementary material.

**Supplementary Material 1:** Figure S1 – S24

**Supplementary Material 2: File S1.** Drought-responsive genes (DTGs) from 18 individual comparisons

**Supplementary Material 3: File S2.** 10 Clusters of 3582 DTGs

**Supplementary Material 4: File S3.** Developmentally-responsive genes (DVGs) from 30 individual comparisons

**Supplementary Material 5: File S4.** 9 clusters of 8694 DVGs

**Supplementary Material 6: File S5.** Aging-related genes (ARGs) from 27 individual comparisons with Day 0

**Supplementary Material 7: File S6.** Aging-related genes (ARGs) from 27 individual comparisons between Day 5, 6 and 7

**Supplementary Material 8: File S7.** 10 clusters of 6491 ARGs

**Supplementary Material 9: File S8.** RPKM for all annotated genes in the 30 treatments

**Supplementary Material 10: File S9.** Quality control of RNA samples and mapping

## Data Availability

All data are available in the main text and supplemental materials. The assembled RNA-seq data is available at the NCBI SRA database through accession number PRJNA947738.
