# Supplementary material for: Developmentally dependent reprogramming of the Arabidopsis floral transcriptome under sufficient and limited water availability
Source: BMC Plant Biol. 2024 Apr 11;24:273. doi: 10.1186/s12870-024-04916-w (PMC11007919; doi:10.1186/s12870-024-04916-w)
Supplement: Supplementary file 1 — Supplementary Material 1: Figure S1 – S24 [file 12870_2024_4916_MOESM1_ESM.pdf]

Figure S1

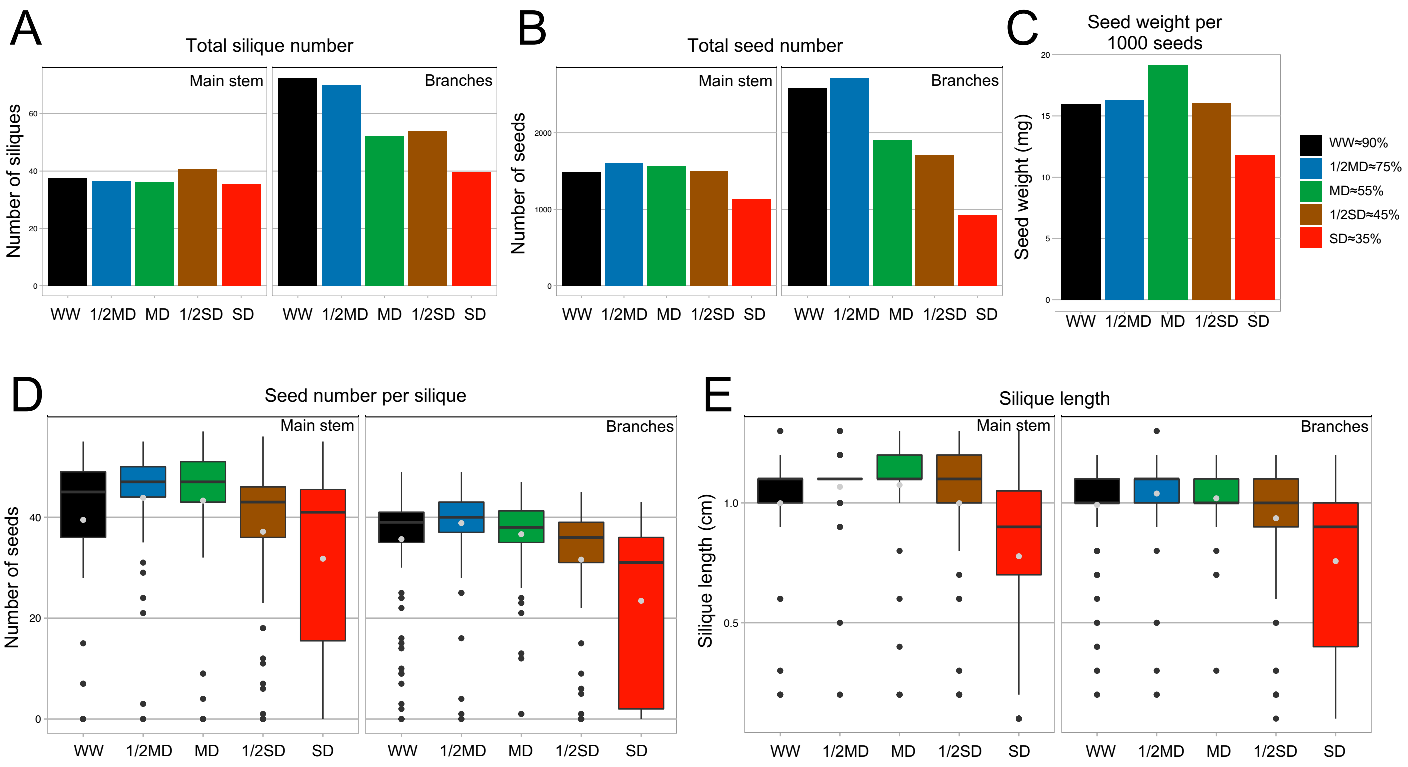

Fig S1. Phenotypic characterization of *Arabidopsis* reproductive development under multiple water conditions. **A-B**. The total number of siliques and the total number of seeds on the main stem or on the branches under different water conditions. n=2. **C**. Seed weight every 1000 seeds under different water conditions. Seed weight every 1000 seeds = (the weight of all seeds from an individual plant (mg) / the total number of seeds from this plant) x 1000. n=2. **D-E**. Seed number per silique and silique length on the main stem or on the branches under different water conditions. n(main stem)= 71-81, n(branches)= 79-145. Solid lines represent one plant under the corresponding water conditions; dashed lines represent the other plant under the corresponding water conditions. Black represents well-watered (WW, ~90% SWC), blue represents slight drought (1/2MD, ~75% SWC), green represents moderate drought (MD, ~55% SWC), brown represents slight severe drought (1/2SD, ~45% SWC), and red represents severe drought (SD, ~35% SWC).

Figure S2

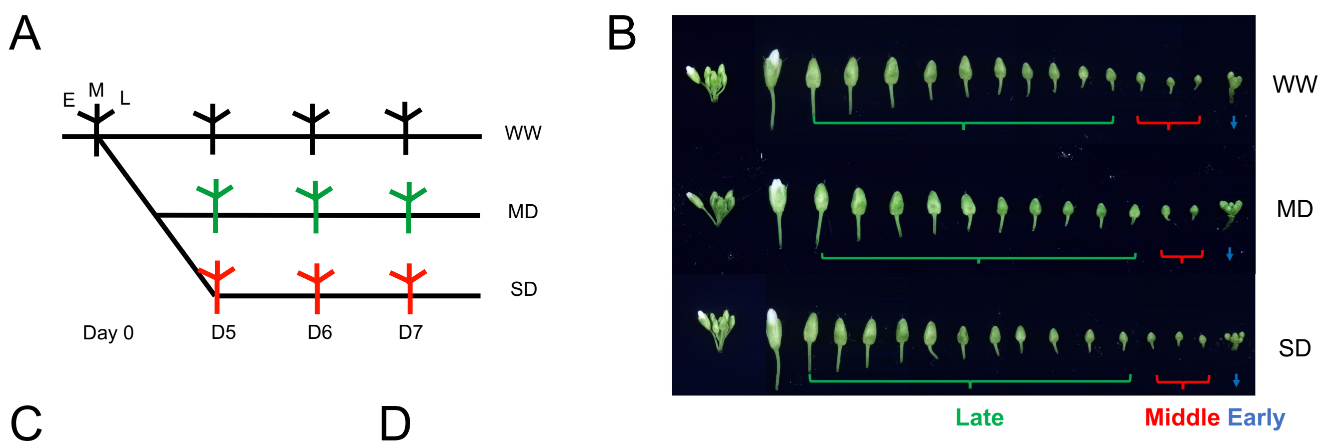

C

Refer. #	30 treatments
011	D0_WW_Early
012	D0_WW_Middle
013	D0_WW_Late
511	D5_WW_Early
512	D5_WW_Middle
513	D5_WW_Late
521	D5_MD_Early
522	D5_MD_Middle
523	D5_MD_Late
531	D5_SD_Early *
532	D5_SD_Middle
533	D5_SD_Late
611	D6_WW_Early
612	D6_WW_Middle
613	D6_WW_Late
621	D6_MD_Early
622	D6_MD_Middle
623	D6_MD_Late
631	D6_SD_Early
632	D6_SD_Middle
633	D6_SD_Late
711	D7_WW_Early
712	D7_WW_Middle
713	D7_WW_Late
721	D7_MD_Early
722	D7_MD_Middle
723	D7_MD_Late
731	D7_SD_Early
732	D7_SD_Middle
733	D7_SD_Late

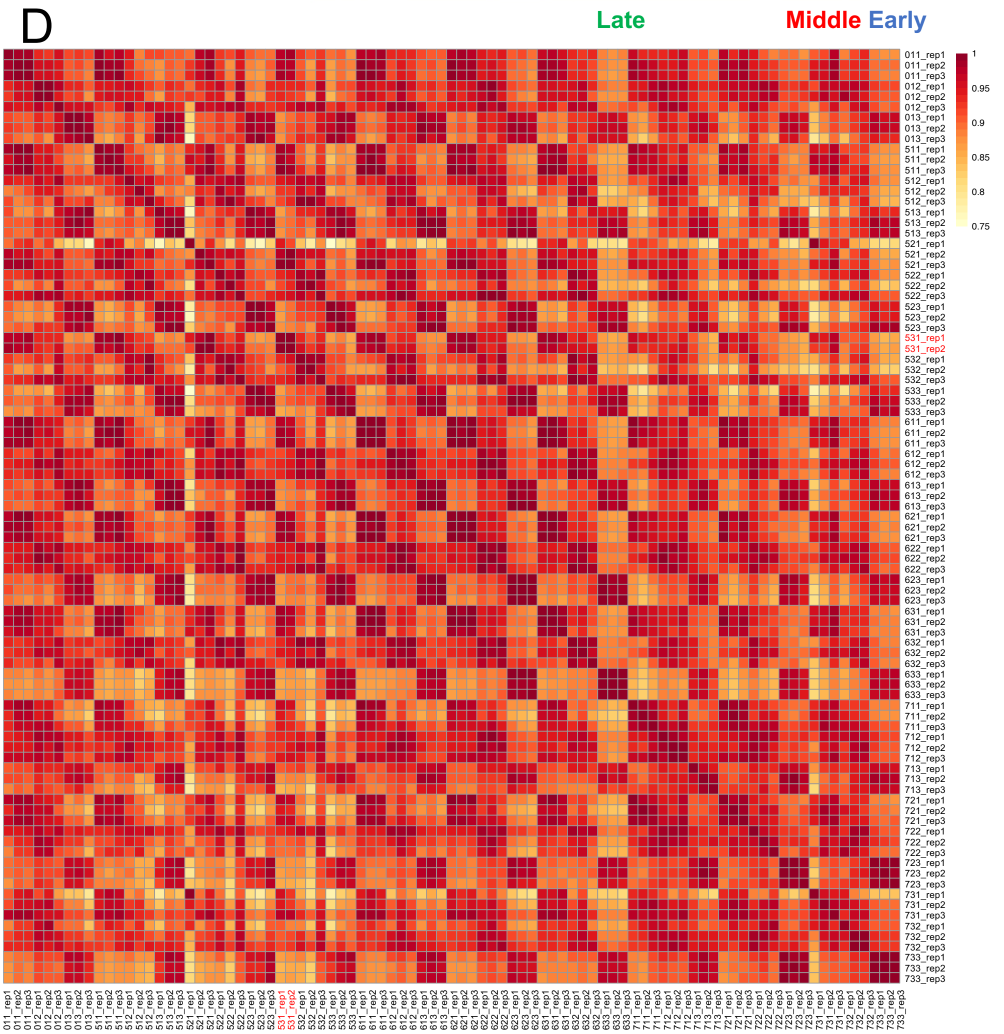

Fig S2. Drought treatment and sampling strategy.
A. Illustration of the transcriptome sampling with 3 consecutive days (after Day 0 as the starting point of the drought treatment) of sample collection under 3 water conditions and phase separation. **B**. Pictures of the whole and dissected inflorescence at different water content. Blue arrows refer to early phase samples, red braces refer to middle phase samples and green braces refer to late phase samples according to our separation. **C**. Sample reference number and sample number annotation. The first digit represents which day the sample was collected (0=Day 0; 5=Day 5; 6=Day 6; 7=Day 7), the second digit indicates the water condition the sample was grown under (1=WW; 2=MD; 3=SD), and the third digit indicates the phase the sample was separated into (1=early; 2=middle; 3=late). **D**. Pearson correlation coefficient of replications for each treatment. * and red font indicates that for 531 (Day 5 severe-drought-treated early phase flower samples) there were only 2 replications.

Figure S3

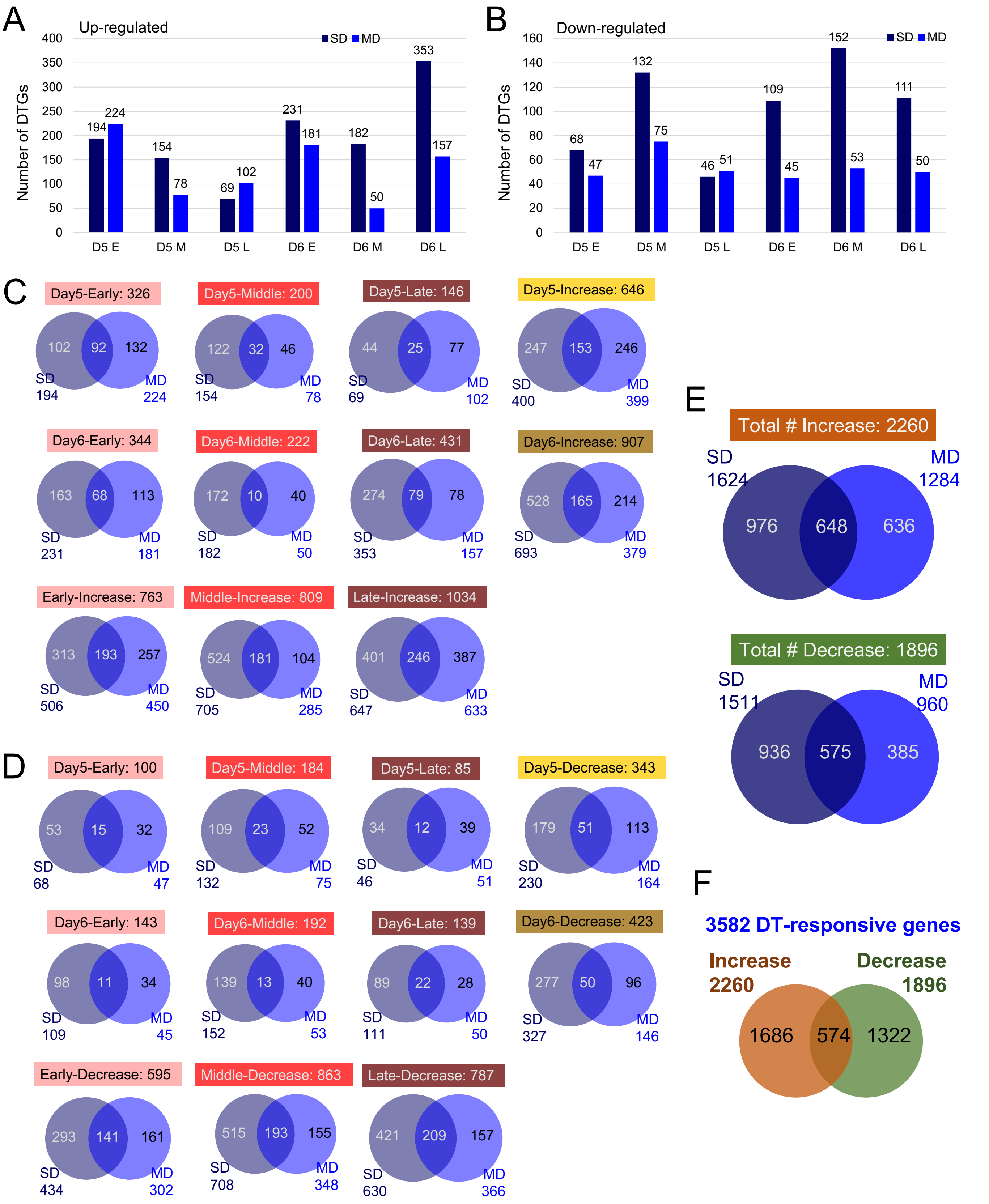

Fig S3. Drought-responsive genes in Day 5 and Day 6 and the summary of all 3582 DTGs.
A-B. Total number of DTGs under SD or MD at each developmental phase on Day 5 and Day 6. **A**: up-regulated genes; **B**: down-regulated genes. **C-D**. Comparison of DTGs under SD and MD on Day 5 and Day 6 at each developmental phase and the summarizing comparison including all 3 developmental phases or all 3 days. **C**: up-regulated genes; **D**: down-regulated genes. First row: Day 5; second row: Day 6; third row: DTGs from all 3 days. First column: early phase; second column: middle phase; third column: late phase; fourth column: DTGs from all 3 phases. **E**. Comparison of the total number of DTGs under SD and MD. Top: up-regulated genes; bottom: down-regulated genes. **F**. All 3582 drought-responsive genes. Dark blue represents SD (expression level under SD compared to WW), blue represents MD (expression level under MD compared to WW).

Figure S4

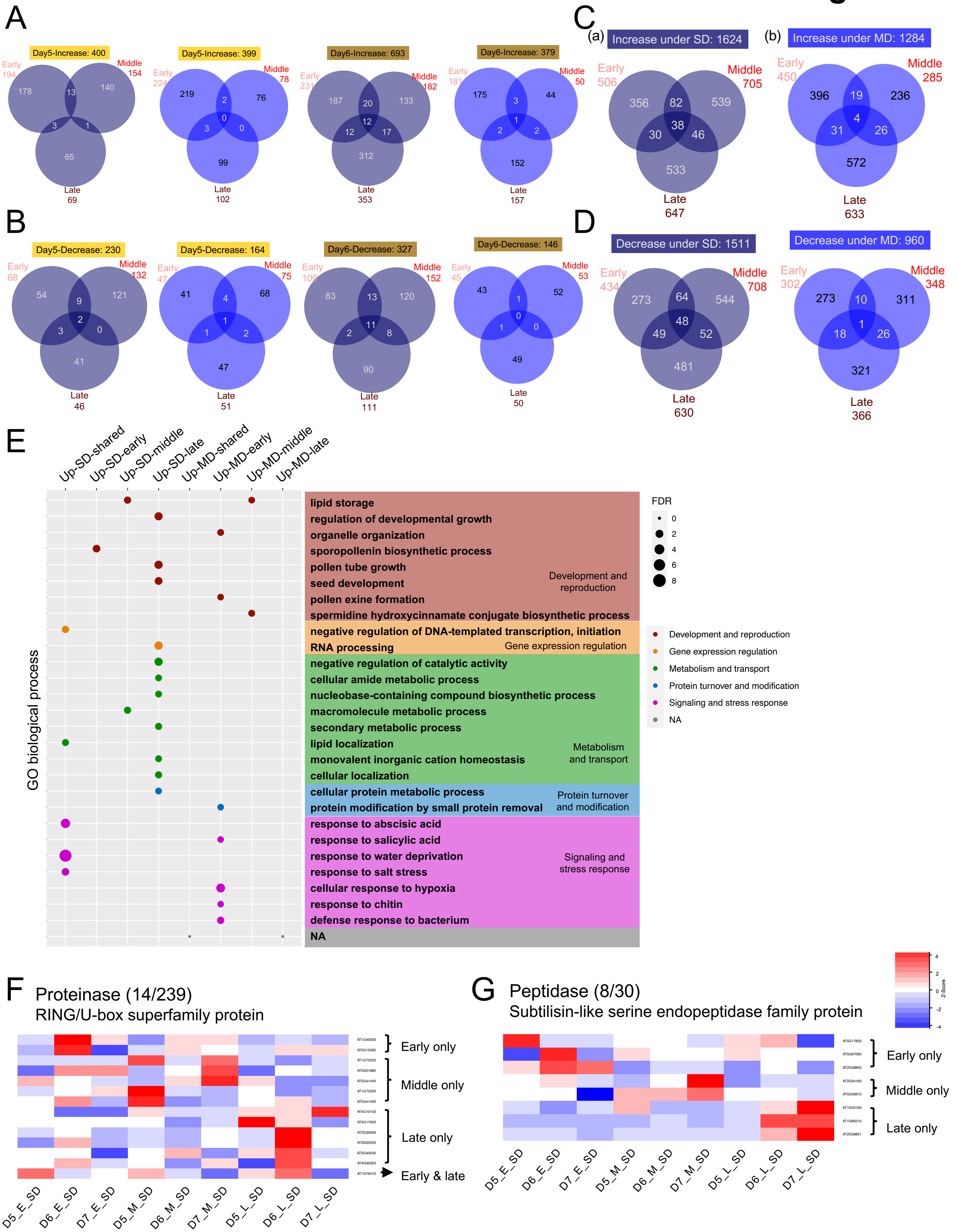
A
Day5-Increase: 400
Early 194
Middle 154
Late 69
Day5-Increase: 399
Early 224
Middle 78
Late 102
Day6-Increase: 693
Early 231
Middle 182
Late 353
Day6-Increase: 379
Early 181
Middle 50
Late 157
B
Day5-Decrease: 230
Early 68
Middle 132
Late 46
Day5-Decrease: 164
Early 47
Middle 75
Late 51
Day6-Decrease: 327
Early 109
Middle 152
Late 111
Day6-Decrease: 146
Early 45
Middle 53
Late 50
C
(a) Increase under SD: 1624
Early 506
Middle 705
Late 647
(b) Increase under MD: 1284
Early 450
Middle 285
Late 633
D
Decrease under SD: 1511
Early 434
Middle 708
Late 630
Decrease under MD: 960
Early 302
Middle 348
Late 366
E
Up-SD-shared
Up-SD-early
Up-SD-middle
Up-SD-late
Up-MD-shared
Up-MD-early
Up-MD-middle
Up-MD-late
GO biological process
lipid storage
regulation of developmental growth
organelle organization
sporopollenin biosynthetic process
pollen tube growth
seed development
pollen exine formation
spermidine hydroxycinnamate conjugate biosynthetic process
Development and reproduction
negative regulation of DNA-templated transcription, initiation
RNA processing
Gene expression regulation
negative regulation of catalytic activity
cellular amide metabolic process
nucleobase-containing compound biosynthetic process
macromolecule metabolic process
secondary metabolic process
lipid localization
monovalent inorganic cation homeostasis
cellular localization
Metabolism and transport
cellular protein metabolic process
protein modification by small protein removal
Protein turnover and modification
response to abscisic acid
response to salicylic acid
response to water deprivation
response to salt stress
cellular response to hypoxia
response to chitin
defense response to bacterium
Signaling and stress response
NA
FDR
0
2
4
6
8
Development and reproduction
Gene expression regulation
Metabolism and transport
Protein turnover and modification
Signaling and stress response
NA
F
Proteinase (14/239)
RING/U-box superfamily protein
Early only
Middle only
Late only
Early & late
D5_E_SD
D6_E_SD
D7_E_SD
D5_M_SD
D6_M_SD
D7_M_SD
D5_L_SD
D6_L_SD
D7_L_SD
G
Peptidase (8/30)
Subtilisin-like serine endopeptidase family protein
Z-Score
Early only
Middle only
Late only
D5_E_SD
D6_E_SD
D7_E_SD
D5_M_SD
D6_M_SD
D7_M_SD
D5_L_SD
D6_L_SD
D7_L_SD

Fig S4. Comparison of DTGs in Day 5 and Day 6 between different drought conditions.
A-B. Comparison of DTGs between the 3 developmental phases on Day 5 and Day 6. **A**: up-regulated genes; **B**: down-regulated genes. First 2 panels are Day 5, last 2 panels are Day 6; left panel: SD; right panel: MD. **C-D**. Comparison of DTGs between the 3 developmental phases including all 3 days. **C**: up-regulated genes; **D**: down-regulated genes. Left (a): SD; right (b): MD. **E**. GO enrichment of specific subsets of DTGs from **C**. Colors of the dots refer to different general biological processes, and sizes of the dots refer to the level of enrichment (-$\log_{10}$FDR). **F-G**. Heatmaps demonstrating the fold change of different sets of functional genes – different up-regulated DTG families that are involved in protein degradation. Different family members were induced by SD at different developmental phases. The number of genes involved in the corresponding analyses (from each gene family) and the total number of genes from each gene family are shown in the parentheses. Different comparisons (at specific developmental phases on different days) are presented horizontally, and each individual gene is presented vertically. Gene IDs are provided on the right side of the heatmaps, and the expression pattern changes of each gene is annotated on the right side of the gene IDs. Dark blue represents SD (expression level under SD compared to WW), blue represents MD (expression level under MD compared to WW).

Figure S5

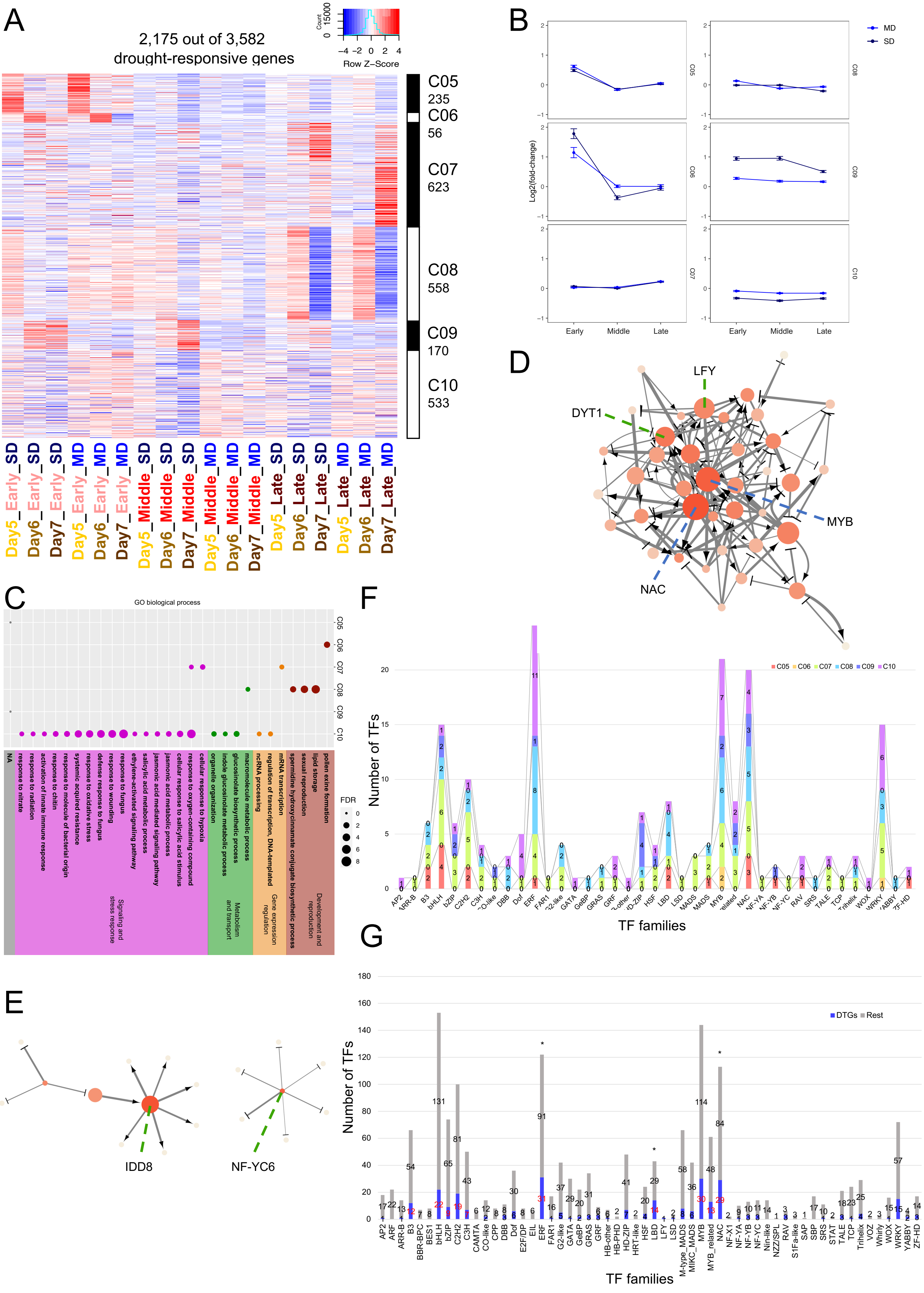
A
2,175 out of 3,582
drought-responsive genes
Count
Row Z-Score
C05
235
C06
56
C07
623
C08
558
C09
170
C10
533
Day5_Early_SD
Day6_Early_SD
Day7_Early_SD
Day5_Early_MD
Day6_Early_MD
Day7_Early_MD
Day5_Middle_SD
Day6_Middle_SD
Day7_Middle_SD
Day5_Middle_MD
Day6_Middle_MD
Day7_Middle_MD
Day5_Late_SD
Day6_Late_SD
Day7_Late_SD
Day5_Late_MD
Day6_Late_MD
Day7_Late_MD
B
MD
SD
Log2(fold-change)
Early
Middle
Late
C
GO biological process
FDR
Signaling and stress response
Metabolism and transport
Gene expression regulation
Development and reproduction
D
LFY
DYT1
MYB
NAC
E
IDD8
NF-YC6
F
Number of TFs
TF families
C05
C06
C07
C08
C09
C10
G
Number of TFs
TF families
DTGs
Rest

Fig S5. The other 6 clusters of DTGs and gene regulatory networks of TFs from the clusters.
A. Expression dynamics across 3 developmental phases and 3 days of the other 6 clusters (C05-C10) of 2175 DTGs. Red indicates up-regulation, blue indicates down-regulation and white indicates no change. **B**. The average fold change of all genes in the corresponding clusters at each developmental phase under different water conditions (the 3 different days were combined). Dark blue represents SD, blue represents MD. **C**. GO enrichment based on genes from the corresponding clusters. Colors of the dots refer to different general biological processes, and sizes of the dots refer to the level of enrichment ($-\log_{10}$FDR). **D-E**. Gene regulatory networks based on the TFs from Cluster 01 and Cluster 02+03. Node color represents the degree of interaction (the number of edges of a node), node size represents the betweenness of the node (the frequency that this node acts as a bridge along the shortest path between two other nodes), and the edge width represents the weight of the interaction (the probability of the interaction (posterior link probability) that was obtained based on the fold change of the genes in response to drought stress). Arrows refer to positive regulation from the regulators to the targets, and the “T”-end lines refer to negative regulation from the regulators to the targets. The green dashed lines refer to previously studied TFs, and the blue dashed lines refer to novel TFs. **F**. The TFs and TF families that they are from in the corresponding clusters. **G**. The TF distribution across all TF families of the drought-responsive TFs. Red font indicates the members from this family were found in more than half of the clusters; asterisk indicates p-value < 0.05. Blue bar indicates the TFs showed differential expression under drought in each TF family, grey bar indicates the rest of the TFs in the same family.

Figure S6

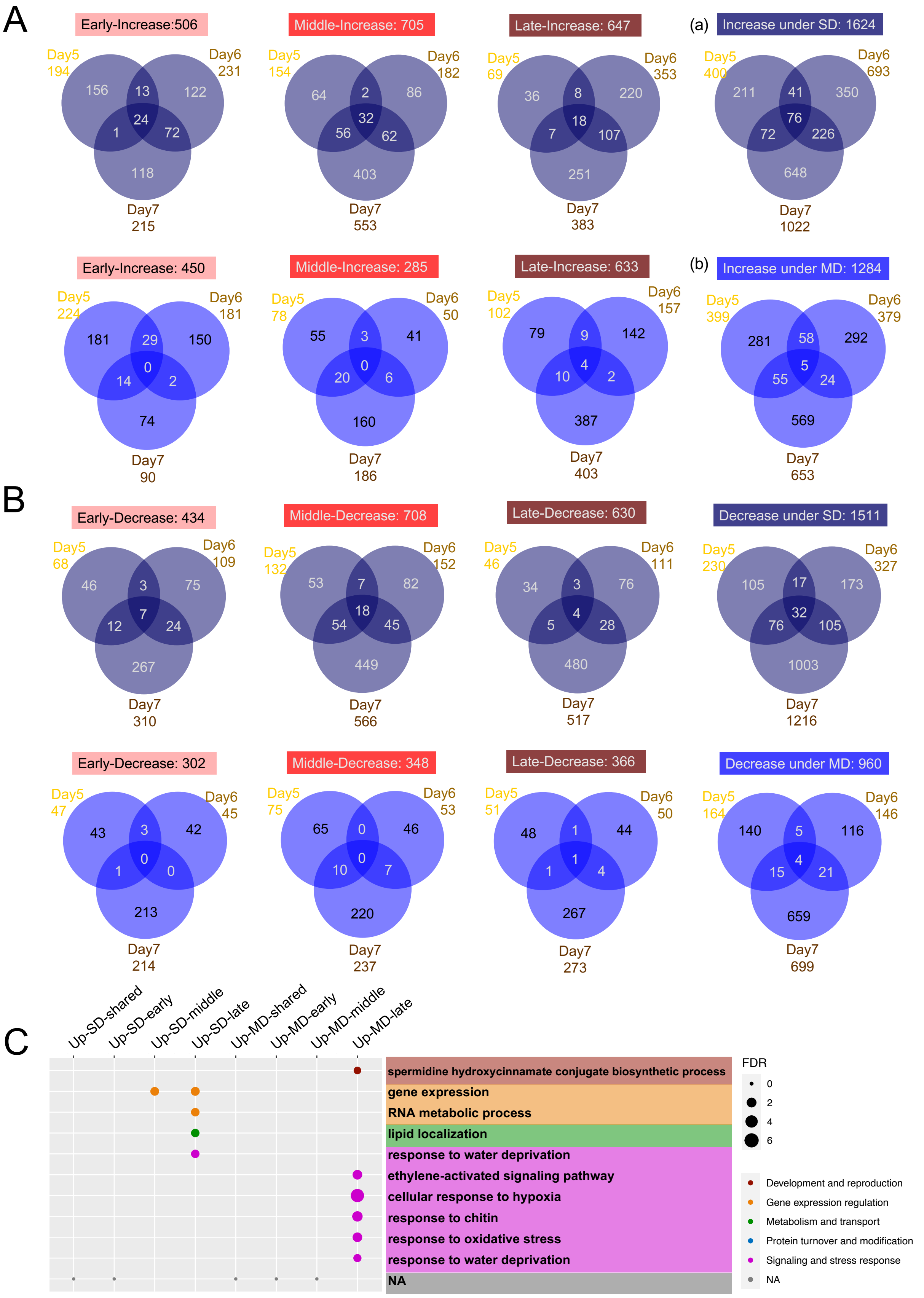

Fig S6. Comparison of DTGs between the 3 days.
A-B. Comparison of DTGs between the 3 different days at the same developmental phase and the summarizing comparison including all 3 developmental phases. **A**: up-regulated genes; **B**: down-regulated genes. First row: SD; second row: MD. First column: early phase; second column: middle phase; third column: late phase; fourth column: DTGs from all 3 phases. **C**. GO enrichment of specific subsets of DTGs from **A**(a) and (b). Colors of the dots refer to different general biological processes, and sizes of the dots refer to the level of enrichment ($-\log_{10}$FDR). Dark blue represents SD (expression level under SD compared to WW), blue represents MD (expression level under MD compared to WW).

Figure S7

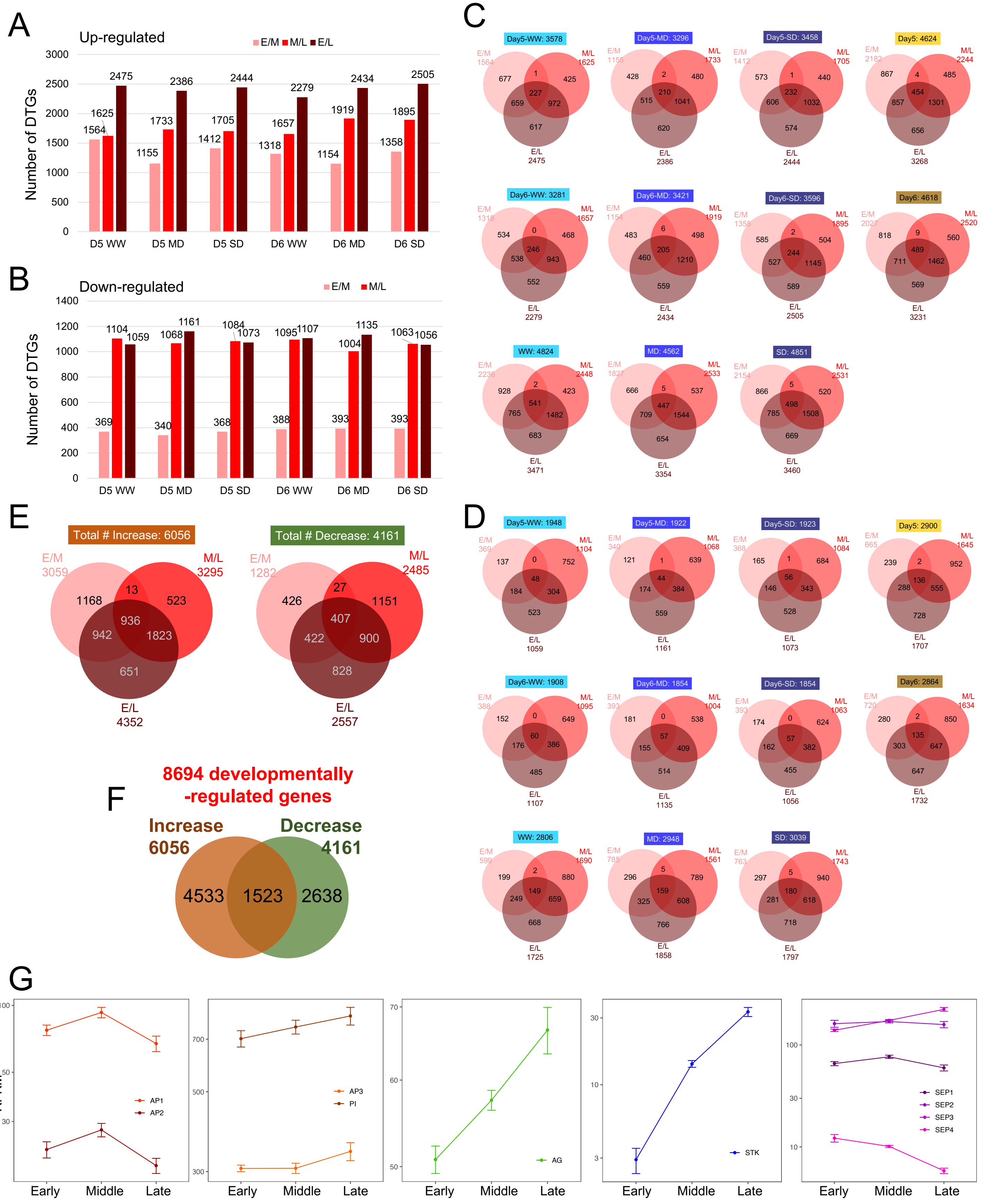

A
Up-regulated
E/M M/L E/L
Number of DTGs
3000 2500 2000 1500 1000 500 0
1564 1625 2475
1155 1733 2386
1412 1705 2444
1318 1657 2279
1154 1919 2434
1358 1895 2505
D5 WW D5 MD D5 SD D6 WW D6 MD D6 SD
B
Down-regulated
E/M M/L
Number of DTGs
1400 1200 1000 800 600 400 200 0
369 1104 1059
340 1068 1161
368 1084 1073
388 1095 1107
393 1004 1135
393 1063 1056
D5 WW D5 MD D5 SD D6 WW D6 MD D6 SD
C
Day5-WW: 3578
E/M 1564 M/L 1625 E/L 2475
677 1 425 227 659 972 617
Day5-MD: 3296
E/M 1155 M/L 1733 E/L 2386
428 2 480 210 515 1041 620
Day5-SD: 3458
E/M 1412 M/L 1705 E/L 2444
573 1 440 232 606 1032 574
Day5: 4624
E/M 2182 M/L 2244 E/L 3268
867 4 485 454 857 1301 656
Day6-WW: 3281
E/M 1318 M/L 1657 E/L 2279
534 0 468 246 538 943 552
Day6-MD: 3421
E/M 1154 M/L 1919 E/L 2434
483 6 498 205 460 1210 559
Day6-SD: 3596
E/M 1358 M/L 1895 E/L 2505
585 2 504 244 527 1145 589
Day6: 4618
E/M 2027 M/L 2520 E/L 3231
818 9 560 489 711 1462 569
WW: 4824
E/M 2236 M/L 2448 E/L 3471
928 2 423 541 765 1482 683
MD: 4562
E/M 1827 M/L 2533 E/L 3354
666 5 537 447 709 1544 654
SD: 4851
E/M 2154 M/L 2531 E/L 3460
866 5 520 498 785 1508 669
D
Day5-WW: 1948
E/M 369 M/L 1104 E/L 1059
137 0 752 48 184 304 523
Day5-MD: 1922
E/M 340 M/L 1068 E/L 1161
121 1 639 44 174 384 559
Day5-SD: 1923
E/M 368 M/L 1084 E/L 1073
165 1 684 56 146 343 528
Day5: 2900
E/M 665 M/L 1645 E/L 1707
239 2 952 136 288 555 728
Day6-WW: 1908
E/M 388 M/L 1095 E/L 1107
152 0 649 60 176 386 485
Day6-MD: 1854
E/M 393 M/L 1004 E/L 1135
181 0 538 57 155 409 514
Day6-SD: 1854
E/M 393 M/L 1063 E/L 1056
174 0 624 57 162 382 455
Day6: 2864
E/M 720 M/L 1634 E/L 1732
280 2 850 135 303 647 647
WW: 2806
E/M 599 M/L 1690 E/L 1725
199 2 880 149 249 659 668
MD: 2948
E/M 785 M/L 1561 E/L 1858
296 5 789 159 325 608 766
SD: 3039
E/M 763 M/L 1743 E/L 1797
297 5 940 180 281 618 718
E
Total # Increase: 6056
E/M 3059 M/L 3295 E/L 4352
1168 13 523 936 942 1823 651
Total # Decrease: 4161
E/M 1282 M/L 2485 E/L 2557
426 27 1151 407 422 900 828
F
8694 developmentally -regulated genes
Increase 6056
Decrease 4161
4533 1523 2638
G
RPKM
AP1 AP2
AP3 PI
AG
STK
SEP1 SEP2 SEP3 SEP4
Early Middle Late

Fig S7. Developmentally-regulated genes in Day 5 and Day 6 and the summary of all 8694 DVGs.
A-B. Total number of DVGs in early vs middle, middle vs late, and early vs late phase comparisons under each water condition on Day 5 and Day 6. **A**: up-regulated genes; **B**: down-regulated genes. **C-D**. Comparison between the DVGs from the 3 phase comparisons under the same water condition on Day 5 and Day 6. **C**: up-regulated genes; **D**: down-regulated genes. First row: Day 5; second row: Day 6; third row: DVGs from all 3 days. First column: WW; second column: MD; third column: SD; fourth column: DVGs from all water conditions. **E**. Comparison of total number of DVGs from the 3 phase comparisons. Left: up-regulated genes; right: down-regulated genes. **F**. All 8694 developmentally-regulated genes. **G**. The expression level (average RPKM values between the 3 days) of ABC model genes under the well-watered condition throughout the 3 reproductive developmental phases. Light red represents E/M (expression level at middle phase compared to early phase), red represents M/L (expression level at late phase compared to middle phase), dark red represents E/L (expression level at late phase compared to early phase).

Figure S8

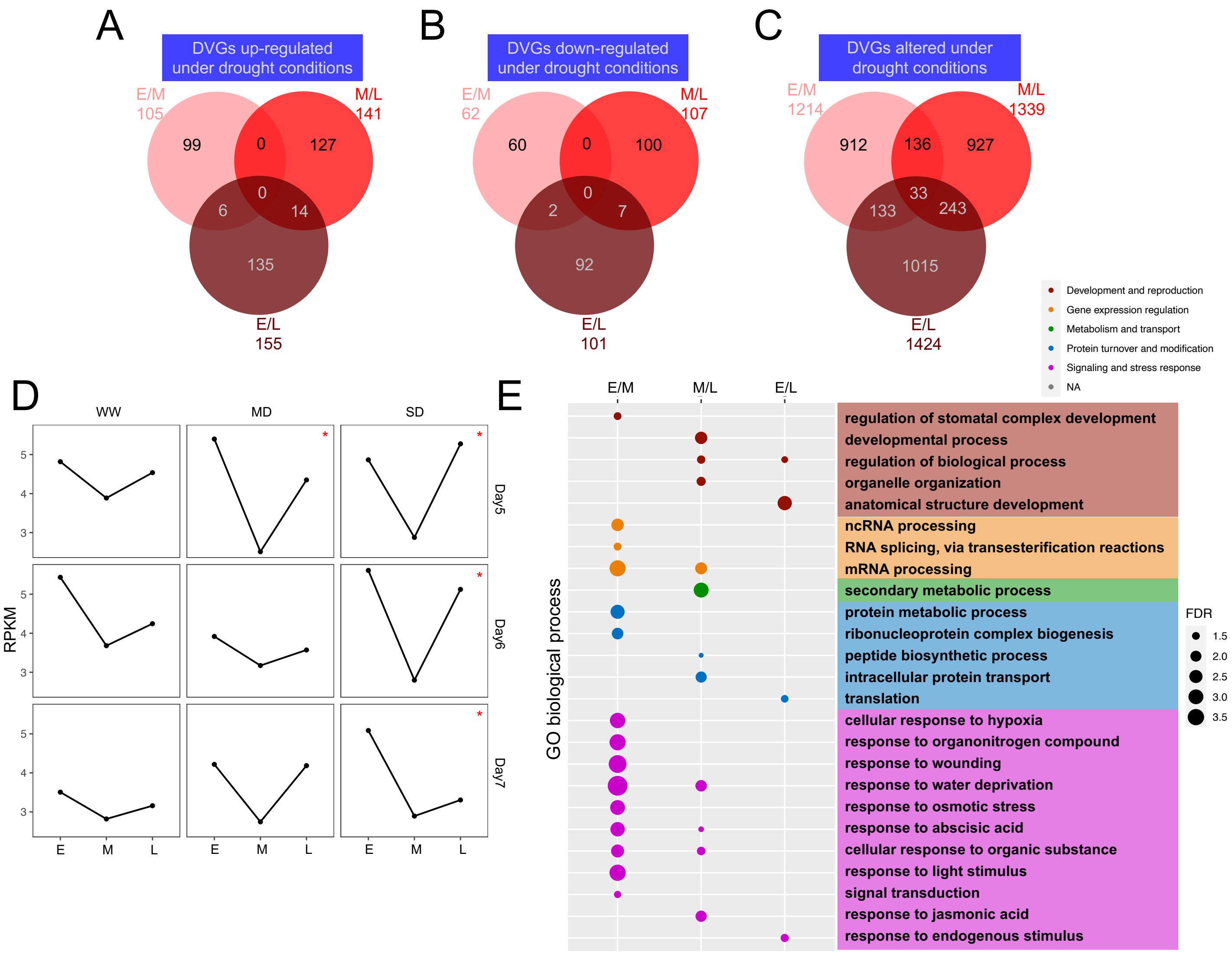

Fig S8. DVGs that showed differential expression specifically under drought conditions.
A-B. Comparison between DVGs that were only altered under drought conditions (differential expression in at least 3 out of all 6 drought samples) but not changed in WW from the 3 phase comparisons. **A**: up-regulated genes; **B**: down-regulated genes. **C**. Comparison between DVGs that were only altered under drought conditions (differential expression in 1-2 out of all 6 drought samples) but not changed in WW from the 3 phase comparisons. **D**. The RPKM values of *WRKY12* at different developmental phases under different water conditions on different days as one example of DVGs only altered under drought conditions. **E**. GO enrichment of the DVGs that were only altered under drought conditions. Colors of the dots refer to different general biological processes, and sizes of the dots refer to the level of enrichment ($-\log_{10}$FDR). Light red represents E/M (expression level at middle phase compared to early phase), red represents M/L (expression level at late phase compared to middle phase), dark red represents E/L (expression level at late phase compared to early phase).

Figure S9

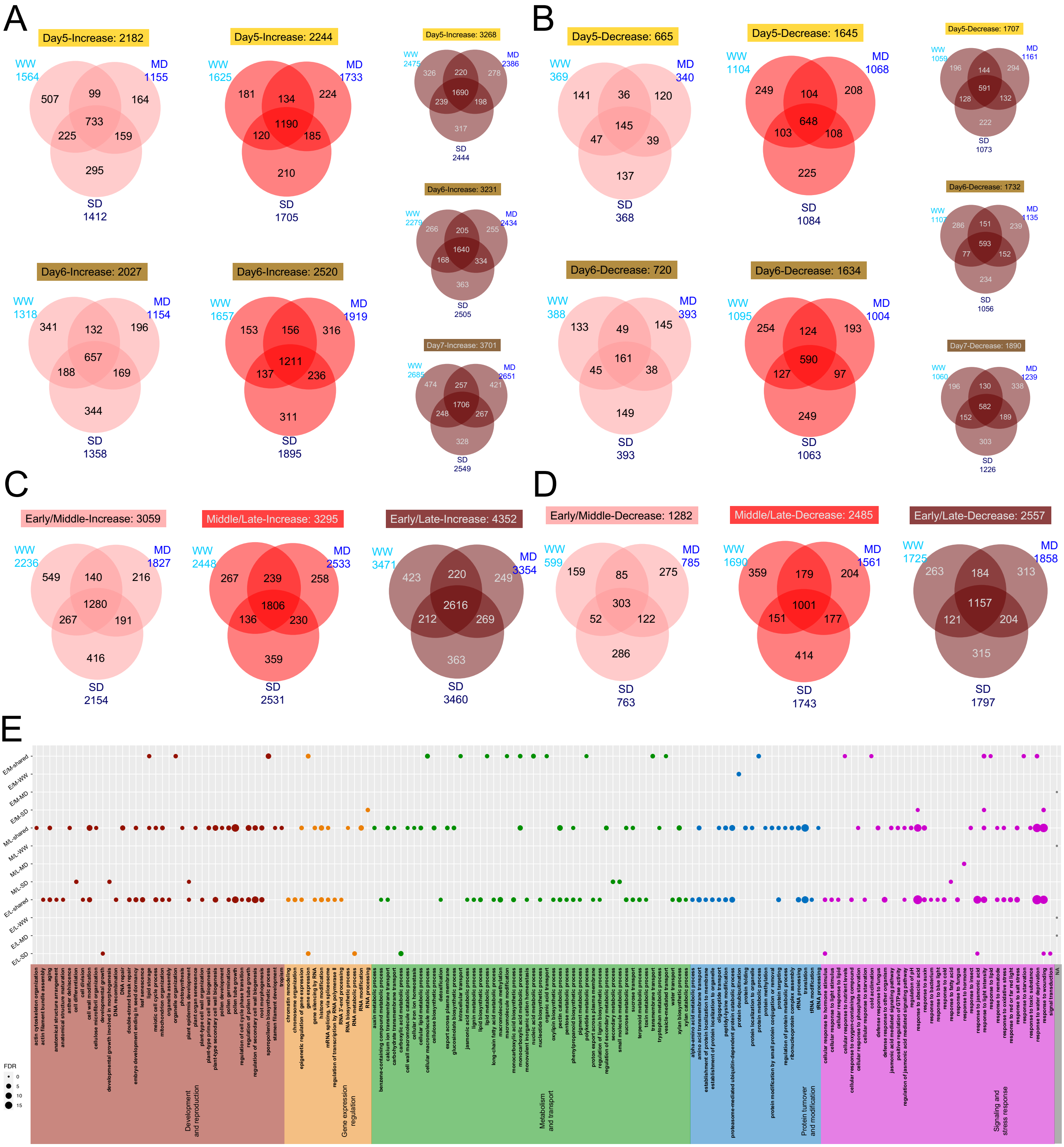

Fig S9. Comparison of DVGs between the 3 water conditions.
A-B. Comparison of DVGs from the same phase comparison under the 3 different water conditions on Day 5 and Day 6, and E/L comparison from Day 7. **A**: up-regulated genes; **B**: down-regulated genes. First row: Day 5; second row: Day 6; third row: Day 7. First column: E/M; second column: M/L; third column: E/L. **C-D**. Comparison of DVGs from the same phase comparison from all 3 days under the 3 different water conditions. **C**: up-regulated genes; **D**: down-regulated genes. Left: E/M; middle: M/L; right: E/L. **E**. GO enrichment of specific subsets of DVGs from **C**. Colors of the dots refer to different general biological processes, and sizes of the dots refer to the level of enrichment ($-\log_{10}$FDR). Light red represents E/M (expression level at middle phase compared to early phase), red represents M/L (expression level at late phase compared to middle phase), dark red represents E/L (expression level at late phase compared to early phase).

Figure S10

A

Protein kinase superfamily protein (MAPK) (78/432)

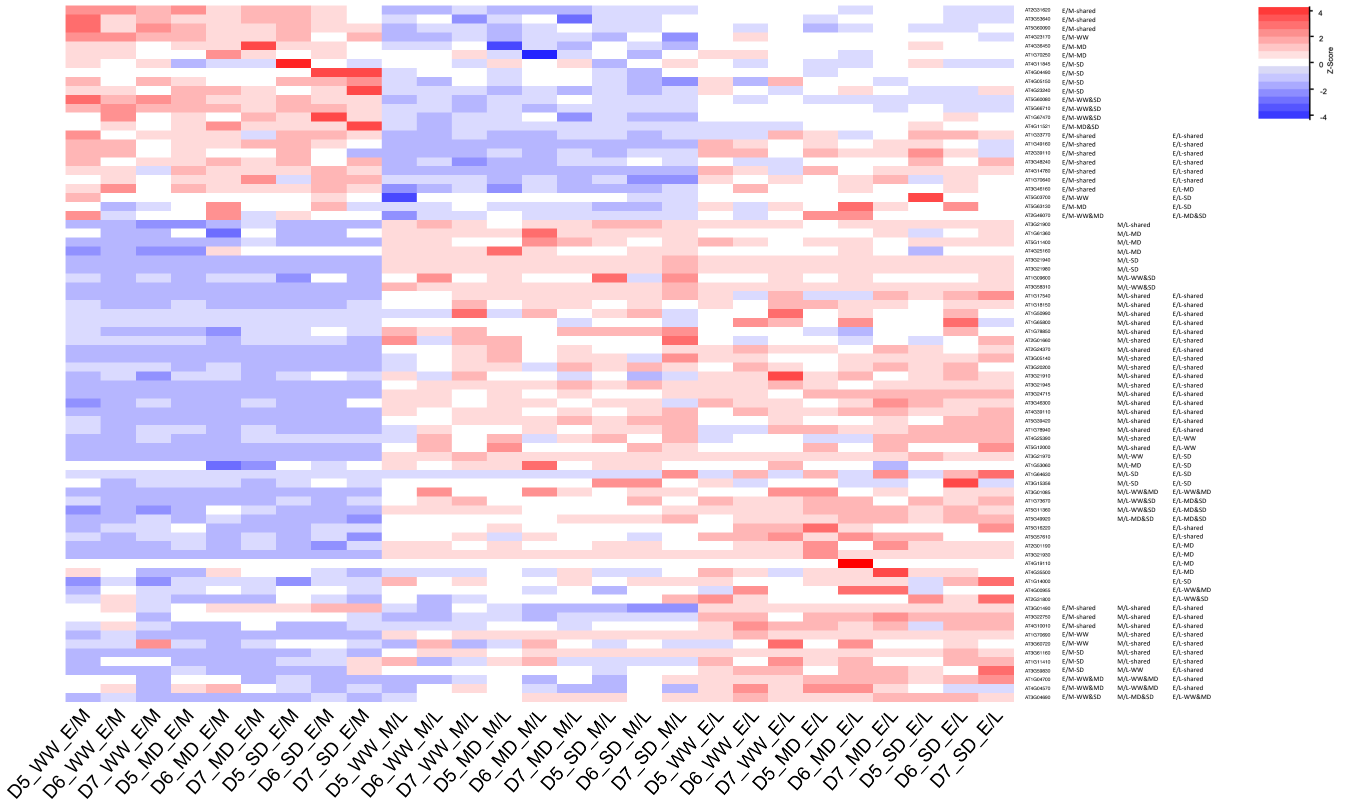

B

Protein kinase superfamily protein (MAPKKK) (55/194)

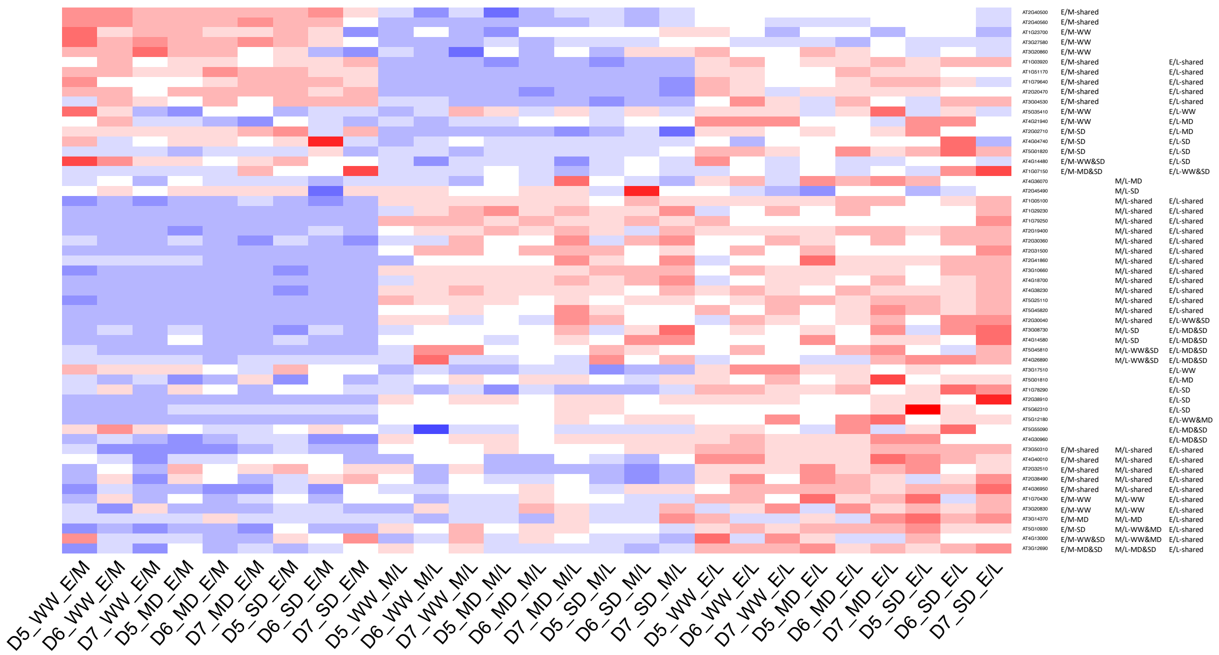

C

Different DUF families induced from different phase comparisons

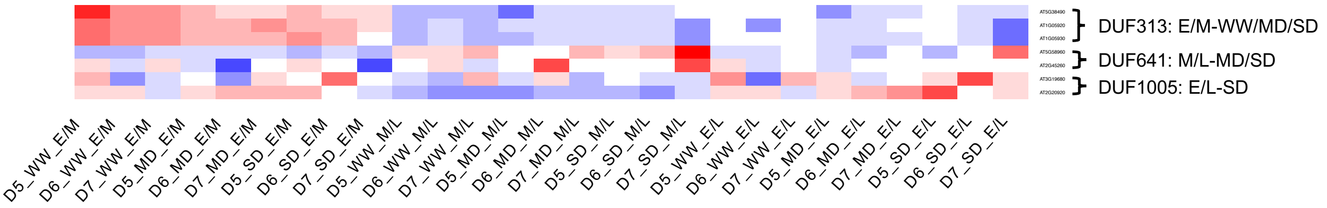

Fig S10. Representative gene families of developmentally-regulated genes.

Heatmaps demonstrating the fold change of different sets of functional genes from the following comparisons. **A-B**. Different up-regulated DVG families that are involved in protein phosphorylation. Different family members were induced during flower development under different water conditions. **C**. Different up-regulated developmentally-regulated domain of unknown function (DUF) gene families that are induced specifically from certain phase comparisons and under certain water conditions. The number of genes involved in the corresponding analyses (from each gene family) and the total number of genes from each gene family are shown in the parentheses. Different comparisons (under specific water conditions on different days) are presented horizontally, and each individual gene is presented vertically. Gene IDs are provided on the right side of the heatmaps, and the expression pattern changes of each gene is annotated on the right side of the gene IDs.

Figure S11

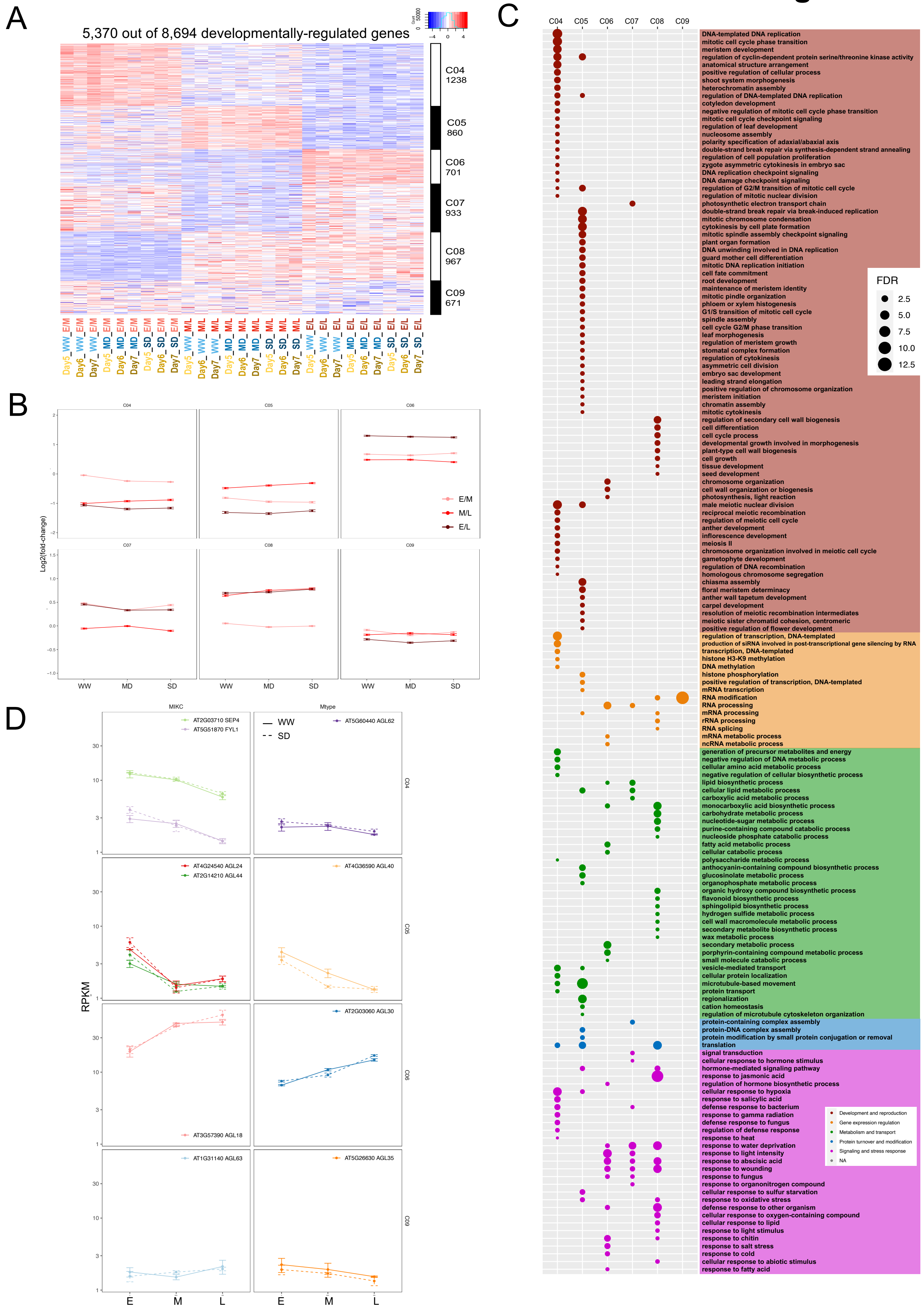
A
5,370 out of 8,694 developmentally-regulated genes
C04 1238
C05 860
C06 701
C07 933
C08 967
C09 671
Day5_WW_E/M
Day6_WW_E/M
Day7_WW_E/M
Day5_MD_E/M
Day6_MD_E/M
Day7_MD_E/M
Day5_SD_E/M
Day6_SD_E/M
Day7_SD_E/M
Day5_WW_M/L
Day6_WW_M/L
Day7_WW_M/L
Day5_MD_M/L
Day6_MD_M/L
Day7_MD_M/L
Day5_SD_M/L
Day6_SD_M/L
Day7_SD_M/L
Day5_WW_E/L
Day6_WW_E/L
Day7_WW_E/L
Day5_MD_E/L
Day6_MD_E/L
Day7_MD_E/L
Day5_SD_E/L
Day6_SD_E/L
Day7_SD_E/L
B
C04
C05
C06
C07
C08
C09
Log2(fold-change)
E/M
M/L
E/L
WW
MD
SD
D
MIKC
Mtype
AT2G03710 SEP4
AT5G51870 FYL1
WW
SD
AT5G60440 AGL62
AT4G24540 AGL24
AT2G14210 AGL44
AT4G36590 AGL40
AT2G03060 AGL30
AT3G57390 AGL18
AT1G31140 AGL63
AT5G26630 AGL35
RPKM
E
M
L
C
C04
C05
C06
C07
C08
C09
FDR
2.5
5.0
7.5
10.0
12.5
DNA-templated DNA replication
mitotic cell cycle phase transition
meristem development
regulation of cyclin-dependent protein serine/threonine kinase activity
anatomical structure arrangement
positive regulation of cellular process
shoot system morphogenesis
heterochromatin assembly
regulation of DNA-templated DNA replication
cotyledon development
negative regulation of mitotic cell cycle phase transition
mitotic cell cycle checkpoint signaling
regulation of leaf development
nucleosome assembly
polarity specification of adaxial/abaxial axis
double-strand break repair via synthesis-dependent strand annealing
regulation of cell population proliferation
zygote asymmetric cytokinesis in embryo sac
DNA replication checkpoint signaling
DNA damage checkpoint signaling
regulation of G2/M transition of mitotic cell cycle
regulation of mitotic nuclear division
photosynthetic electron transport chain
double-strand break repair via break-induced replication
mitotic chromosome condensation
cytokinesis by cell plate formation
mitotic spindle assembly checkpoint signaling
plant organ formation
DNA unwinding involved in DNA replication
guard mother cell differentiation
mitotic DNA replication initiation
cell fate commitment
root development
maintenance of meristem identity
mitotic pindle organization
phloem or xylem histogenesis
G1/S transition of mitotic cell cycle
spindle assembly
cell cycle G2/M phase transition
leaf morphogenesis
regulation of meristem growth
stomatal complex formation
regulation of cytokinesis
asymmetric cell division
embryo sac development
leading strand elongation
positive regulation of chromosome organization
meristem initiation
chromatin assembly
mitotic cytokinesis
regulation of secondary cell wall biogenesis
cell differentiation
cell cycle process
developmental growth involved in morphogenesis
plant-type cell wall biogenesis
cell growth
tissue development
seed development
chromosome organization
cell wall organization or biogenesis
photosynthesis, light reaction
male meiotic nuclear division
reciprocal meiotic recombination
regulation of meiotic cell cycle
anther development
inflorescence development
meiosis II
chromosome organization involved in meiotic cell cycle
gametophyte development
regulation of DNA recombination
homologous chromosome segregation
chiasma assembly
floral meristem determinacy
anther wall tapetum development
carpel development
resolution of meiotic recombination intermediates
meiotic sister chromatid cohesion, centromeric
positive regulation of flower development
regulation of transcription, DNA-templated
production of siRNA involved in post-transcriptional gene silencing by RNA
transcription, DNA-templated
histone H3-K9 methylation
DNA methylation
histone phosphorylation
positive regulation of transcription, DNA-templated
mRNA transcription
RNA modification
RNA processing
mRNA processing
rRNA processing
RNA splicing
mRNA metabolic process
ncRNA metabolic process
generation of precursor metabolites and energy
negative regulation of DNA metabolic process
cellular amino acid metabolic process
negative regulation of cellular biosynthetic process
lipid biosynthetic process
cellular lipid metabolic process
carboxylic acid metabolic process
monocarboxylic acid biosynthetic process
carbohydrate metabolic process
nucleotide-sugar metabolic process
purine-containing compound catabolic process
nucleoside phosphate catabolic process
fatty acid metabolic process
cellular catabolic process
polysaccharide metabolic process
anthocyanin-containing compound biosynthetic process
glucosinolate metabolic process
organophosphate metabolic process
organic hydroxy compound biosynthetic process
flavonoid biosynthetic process
sphingolipid biosynthetic process
hydrogen sulfide metabolic process
cell wall macromolecule metabolic process
secondary metabolite biosynthetic process
wax metabolic process
secondary metabolic process
porphyrin-containing compound metabolic process
small molecule catabolic process
vesicle-mediated transport
cellular protein localization
microtubule-based movement
protein transport
regionalization
cation homeostasis
regulation of microtubule cytoskeleton organization
protein-containing complex assembly
protein-DNA complex assembly
protein modification by small protein conjugation or removal
translation
signal transduction
cellular response to hormone stimulus
hormone-mediated signaling pathway
response to jasmonic acid
regulation of hormone biosynthetic process
cellular response to hypoxia
response to salicylic acid
defense response to bacterium
response to gamma radiation
defense response to fungus
regulation of defense response
response to heat
response to water deprivation
response to light intensity
response to abscisic acid
response to wounding
response to fungus
response to organonitrogen compound
cellular response to sulfur starvation
response to oxidative stress
defense response to other organism
cellular response to oxygen-containing compound
cellular response to lipid
response to light stimulus
response to chitin
response to salt stress
response to cold
cellular response to abiotic stimulus
response to fatty acid
Development and reproduction
Gene expression regulation
Metabolism and transport
Protein turnover and modification
Signaling and stress response
NA

Fig S11. The other 6 clusters of DVGs and transcriptional regulation of reproductive development.
A. Expression dynamics under the 3 different water conditions and across the 3 days of the other 6 clusters (C04-C09) of 5370 DVGs. Red indicates up-regulation, blue indicated down-regulation and white indicates no change. **B**. The average fold change of all genes in the corresponding clusters under different water conditions during reproductive development (the 3 different days were combined). Light red represents E/M, red represents M/L, and dark red represents E/L. **C**. GO enrichment based on genes from the corresponding clusters. Colors of the dots refer to different general biological processes, and sizes of the dots refer to the level of enrichment levels (-$\log_{10}$FDR). **D**. The expression level (average RPKM values between the 3 days under WW or SD across the 3 developmental phases) of MADS-box family genes from the corresponding clusters. Left : MIKC MADS-box genes; right : M-type MADS-box genes. First row: genes from C04; second row: genes from C05; third row: genes from C06; fourth row: genes from C09. Solid line represents WW, dashed line represents SD.

Figure S12

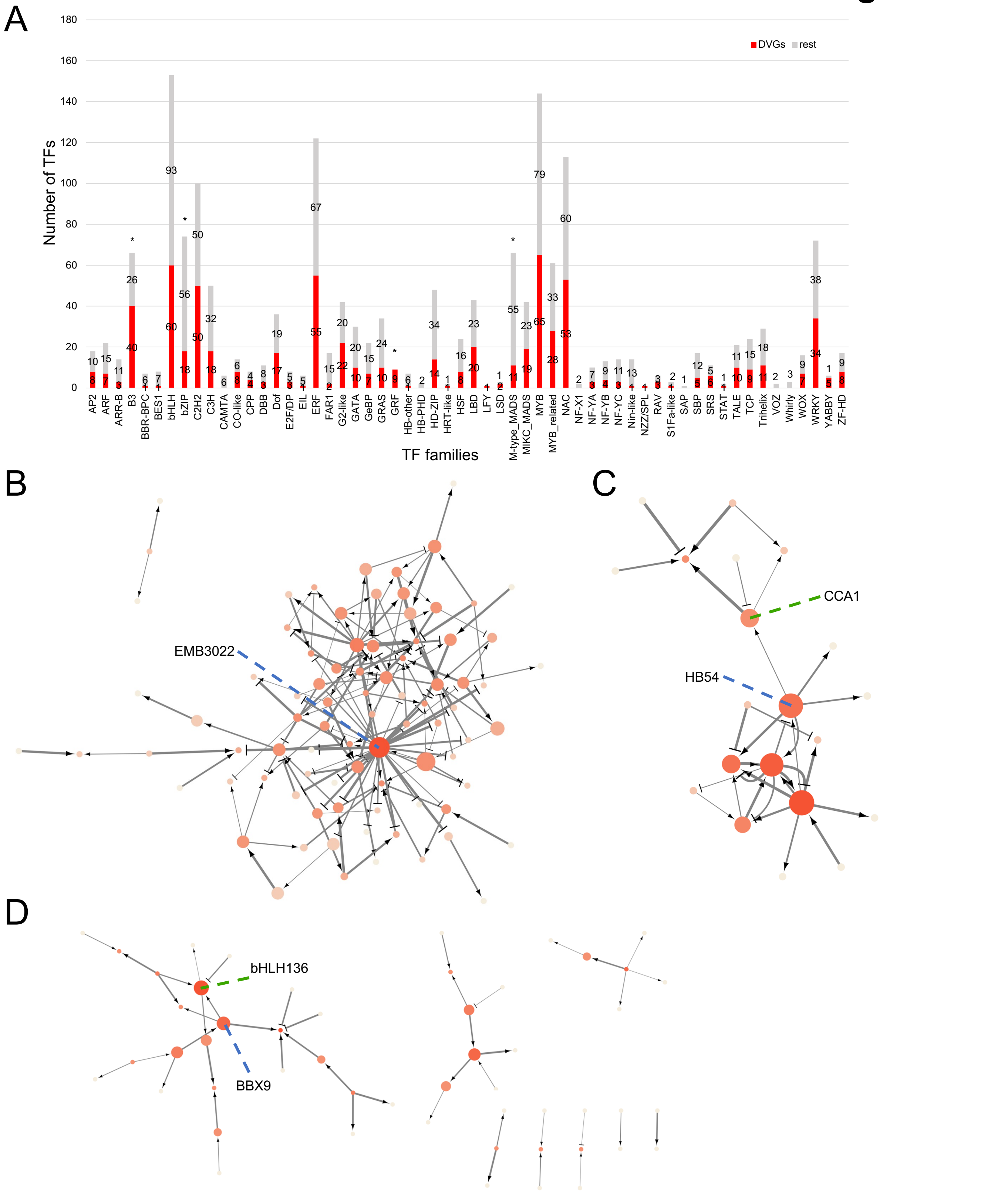

Fig S12. Known genes and GRNs of TFs from the 3 representative DVG clusters.
A. The TF distribution across all TF families of the developmentally-regulated TFs. Asterisk indicates *p*-value < 0.05. Red bar indicates the TFs showed differential expression during development in each TF family, grey bar indicates the rest of the TFs in the same family. **B-D**. Gene regulatory networks based on the TFs from Cluster 01, 02, and 03. Node color represents the degree of interaction (the number of edges of a node), node size represents the betweenness of the node (the frequency that this node acts as a bridge along the shortest path between two other nodes), and the edge width represents the weight of the interaction (the probability of the interaction (posterior link probability) that was obtained based on the fold change of the genes in response to drought stress). Arrows refer to positive regulation from the regulators to the targets, and the “T”-end lines refer to negative regulation from the regulators to the targets. The central nodes were labeled. The green dashed lines refer to previously studied TFs with flower development related functions, and the blue dashed lines refer to TFs with no known function during flower development.

Figure S13

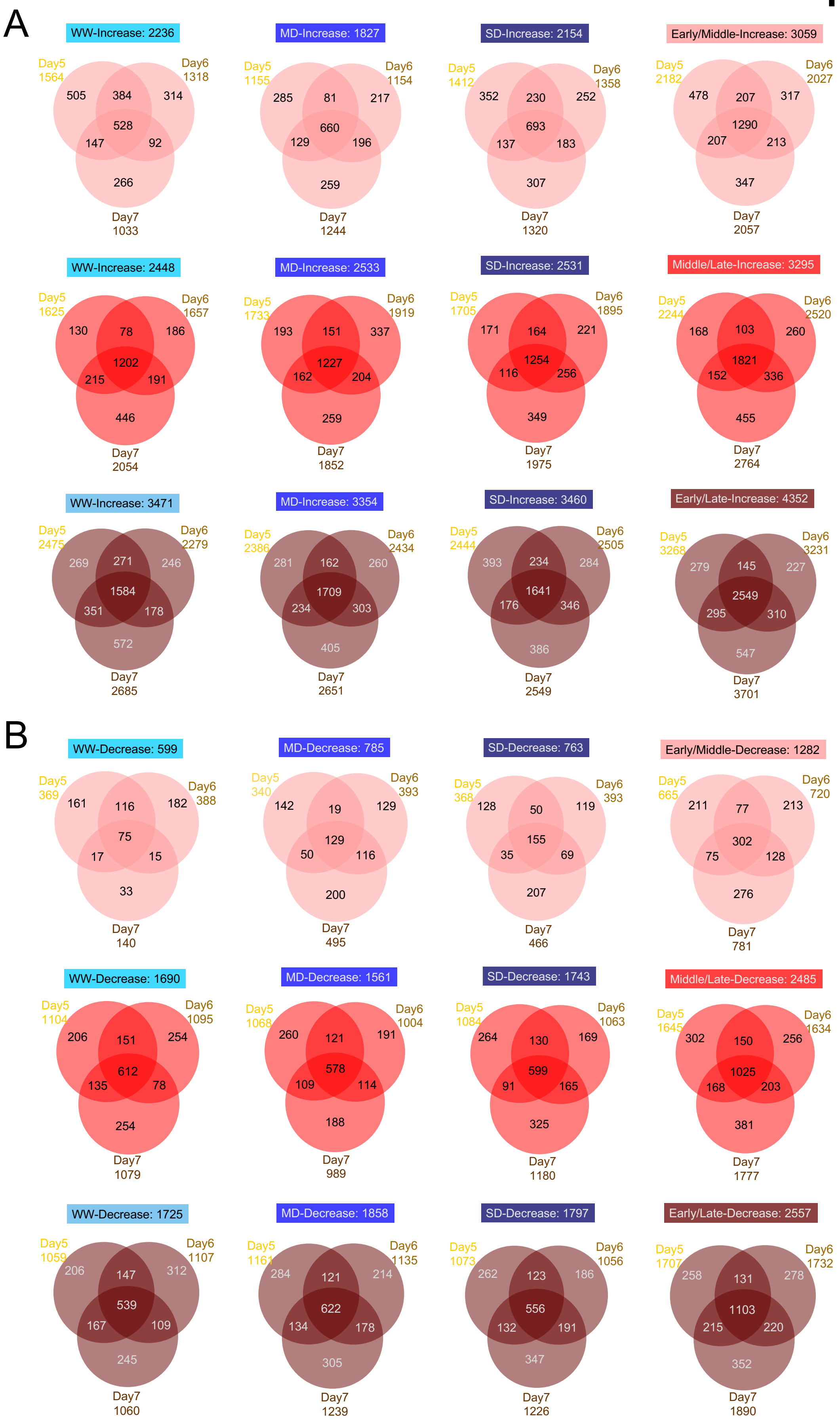

Fig S13. Comparison of DVGs between the 3 days.
A-B. Comparison of DVGs between the 3 different days under the same water condition and the summarizing comparison. **A**: up-regulated genes; **B**: down-regulated genes. First row: E/M; second row: M/L; third row: E/L. First column: WW; second column: MD; third column: SD; fourth column: DVGs from all water conditions. Light red represents E/M (expression level at middle phase compared to early phase), red represents M/L (expression level at late phase compared to middle phase), dark red represents E/L (expression level at late phase compared to early phase).

Figure S14

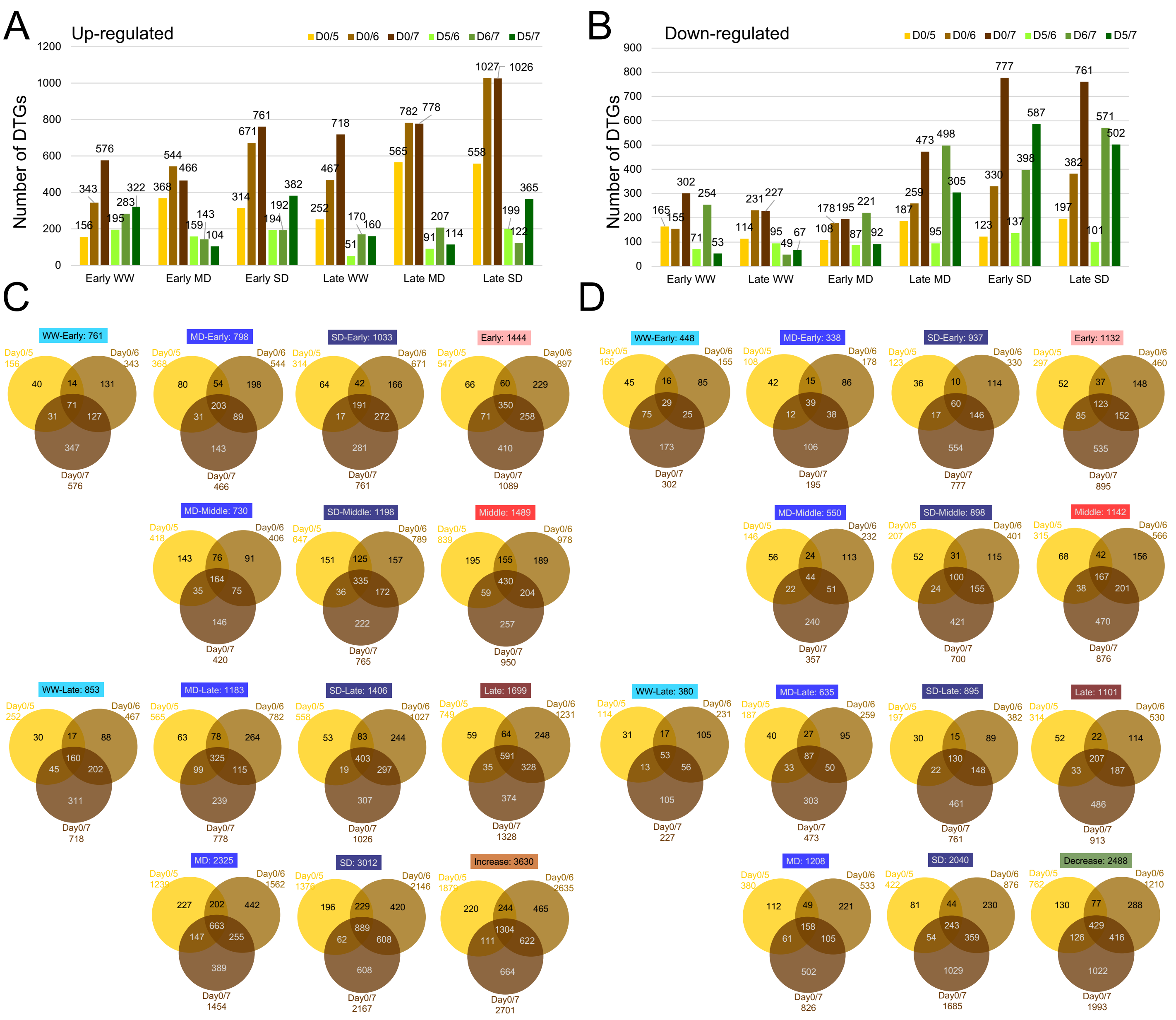

Fig S14. Aging-related genes at early and late phases and the summary of all 6491 ARGs.
A-B. Total number of ARGs in Day 0/5, Day 0/6, Day 0/7, Day 5/6, Day 6/7 and Day 5/7 under each water condition during early and late phases. **A**: up-regulated genes; **B**: down-regulated genes. **C-D**. Comparison between the ARGs from 3 of the 6 day comparisons (Day 0/5, Day 0/6, Day 0/7) under different water conditions at different developmental phases. **C**: up-regulated genes; **D**: down-regulated genes. First row: early phase; second row: middle phase; third row: late phase; fourth row: all 3 phases. First column: WW; second column: MD; third column: SD; fourth column: all 3 water conditions. Yellow represents Day 0/5 (expression level in Day 5 compared to Day 0), brown represents Day 0/6 (expression level in Day 6 compared to Day 0), dark brown represents Day 0/7 (expression level in Day 7 compared to Day 0), light green represents Day 5/6 (expression level in Day 6 compared to Day 5), green represents Day 6/7 (expression level in Day 7 compared to Day 6), dark green represents Day 5/7 (expression level in Day 7 compared to Day 5).

Figure S15

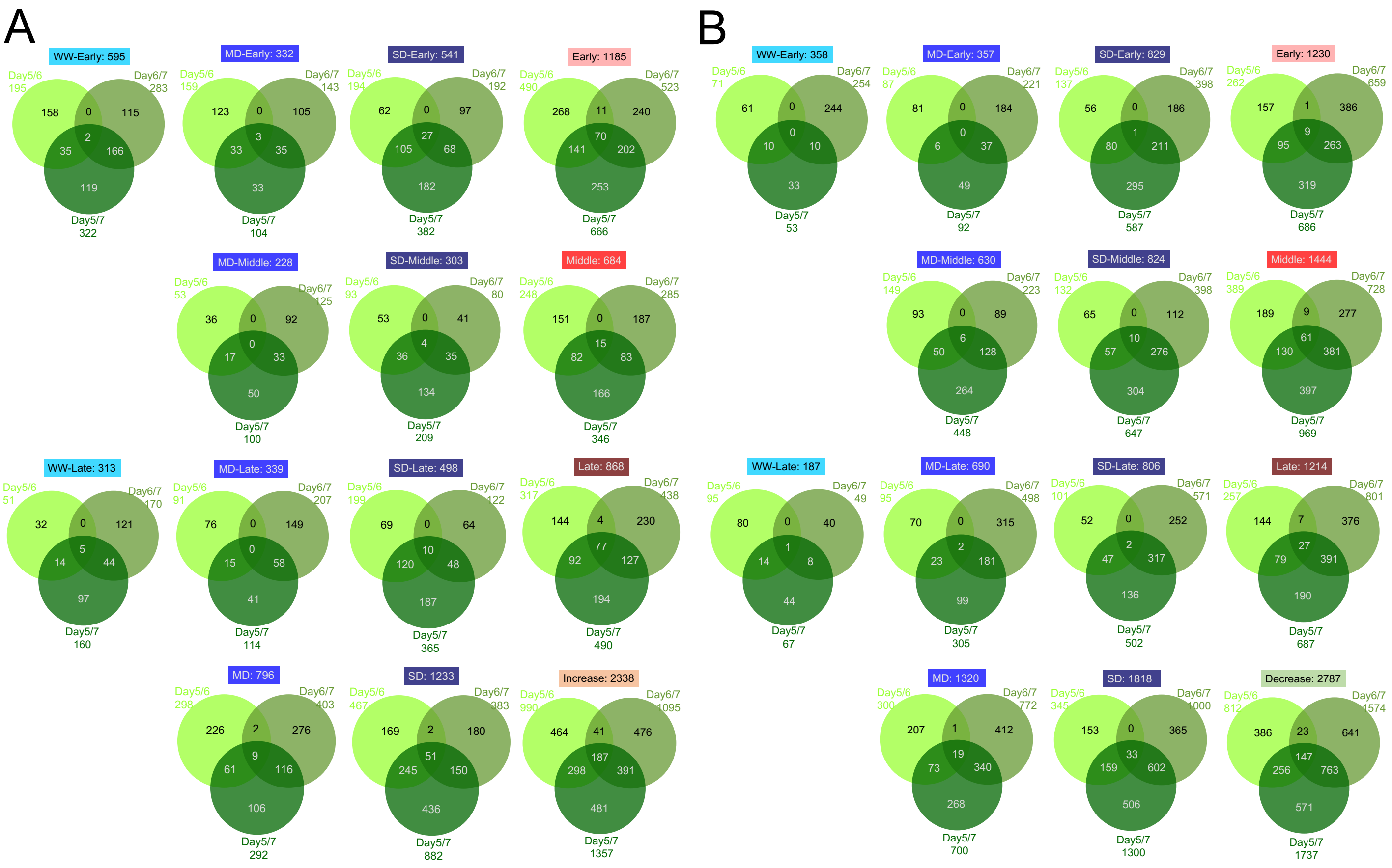

Fig S15. Comparison of ARGs between Day 5, Day 6 and Day 7.
A-B. Comparison between the ARGs from the other 3 of the 6 day comparisons (Day 5/6, Day 6/7, Day 5/7) under different water conditions at different developmental phases. **A**: up-regulated genes; **B**: down-regulated genes. First row: early phase; second row: middle phase; third row: late phase; fourth row: all 3 phases. First column: WW; second column: MD; third column: SD; fourth column: all 3 water conditions. Light green represents Day 5/6 (expression level in Day 6 compared to Day 5), green represents Day 6/7 (expression level in Day 7 compared to Day 6), dark green represents Day 5/7 (expression level in Day 7 compared to Day 5).

Figure S16

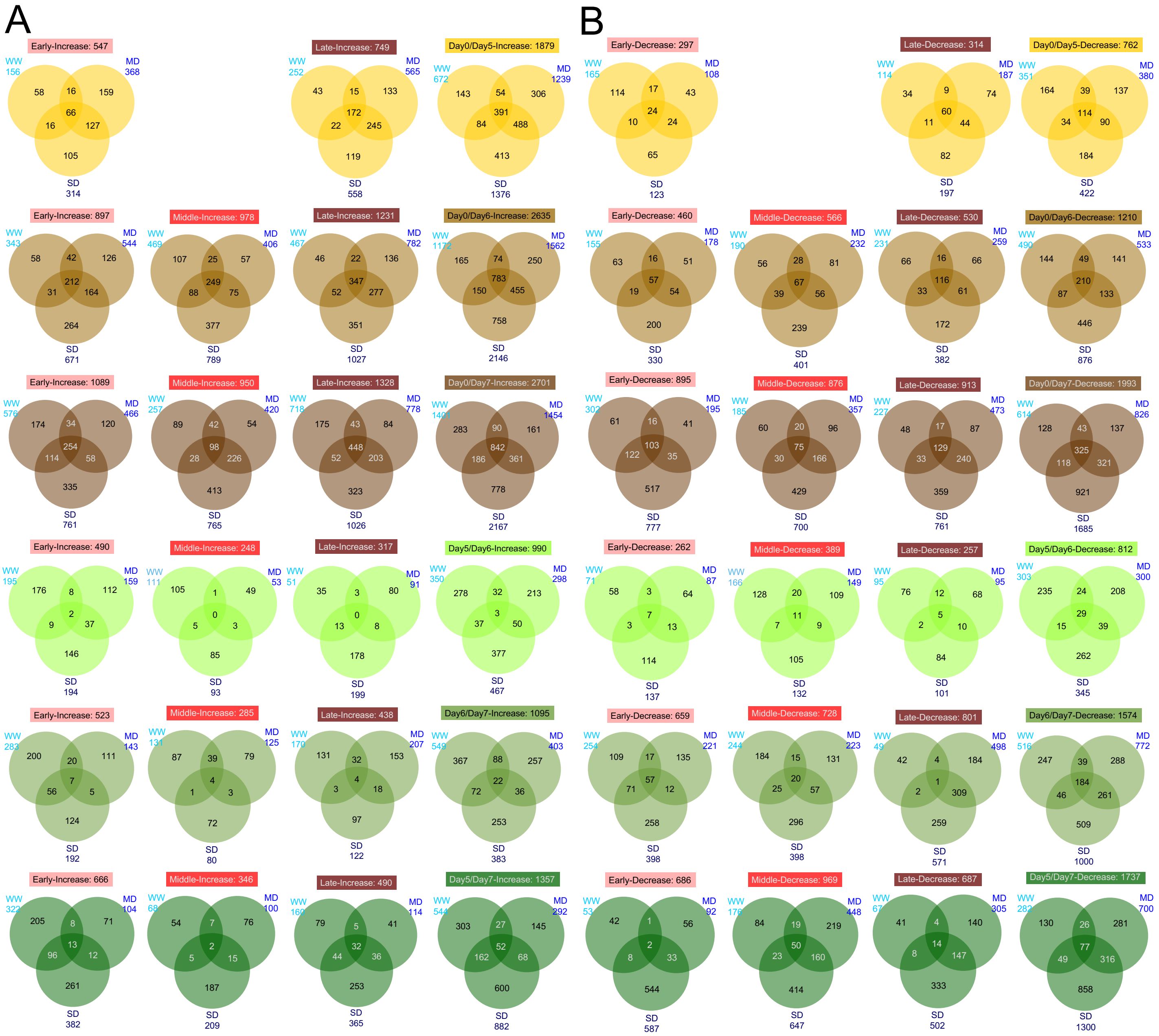

Fig S16. Comparison of ARGs between the 3 water conditions.
A-B. Comparison between the ARGs from the 6 day comparisons under the 3 different water conditions at the same developmental phase. **A**: up-regulated genes; **B**: down-regulated genes. First row: Day 0/5; second row: Day 0/6; third row: Day 0/7; fourth row: Day 5/6; fifth row: Day 6/7; sixth row: Day 5/7. First column: early phase; second column: middle phase; third column: late phase; fourth column: all 3 phases. Yellow represents Day 0/5 (expression level in Day 5 compared to Day 0), brown represents Day 0/6 (expression level in Day 6 compared to Day 0), dark brown represents Day 0/7 (expression level in Day 7 compared to Day 0), light green represents Day 5/6 (expression level in Day 6 compared to Day 5), green represents Day 6/7 (expression level in Day 7 compared to Day 6), dark green represents Day 5/7 (expression level in Day 7 compared to Day 5).

Figure S17

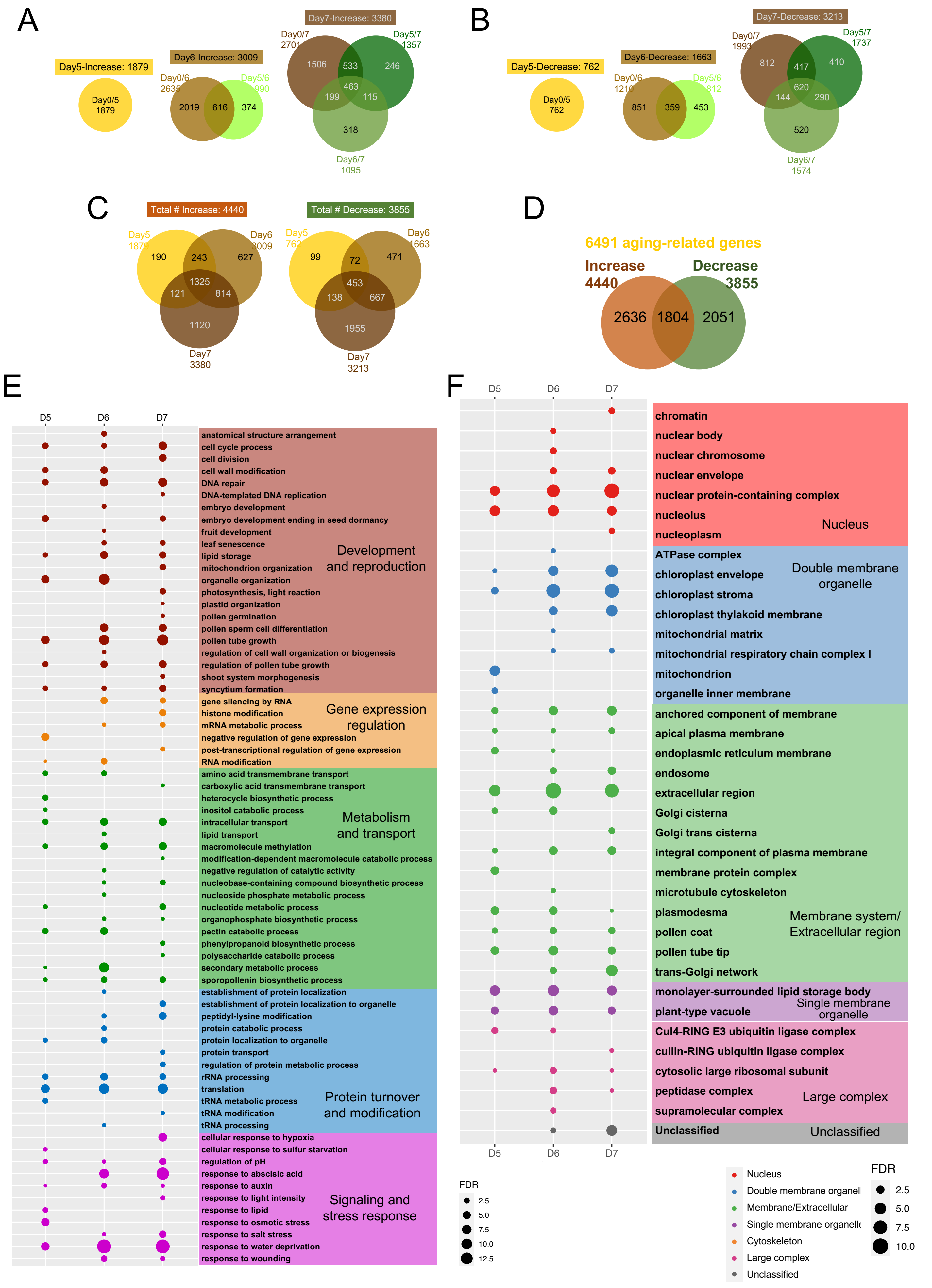
A
Day5-Increase: 1879
Day0/5 1879
Day6-Increase: 3009
Day0/6 2635
Day5/6 990
2019 616 374
Day7-Increase: 3380
Day0/7 2701
Day5/7 1357
Day6/7 1095
1506 533 246 463 199 115 318
B
Day5-Decrease: 762
Day0/5 762
Day6-Decrease: 1663
Day0/6 1210
Day5/6 812
851 359 453
Day7-Decrease: 3213
Day0/7 1993
Day5/7 1737
Day6/7 1574
812 417 410 620 144 290 520
C
Total # Increase: 4440
Day5 1879
Day6 3009
Day7 3380
190 243 627 1325 121 814 1120
Total # Decrease: 3855
Day5 762
Day6 1663
Day7 3213
99 72 471 453 138 667 1955
D
6491 aging-related genes
Increase 4440
Decrease 3855
2636 1804 2051
E
D5 D6 D7
anatomical structure arrangement
cell cycle process
cell division
cell wall modification
DNA repair
DNA-templated DNA replication
embryo development
embryo development ending in seed dormancy
fruit development
leaf senescence
lipid storage
mitochondrion organization
organelle organization
photosynthesis, light reaction
plastid organization
pollen germination
pollen sperm cell differentiation
pollen tube growth
regulation of cell wall organization or biogenesis
regulation of pollen tube growth
shoot system morphogenesis
syncytium formation
Development and reproduction
gene silencing by RNA
histone modification
mRNA metabolic process
negative regulation of gene expression
post-transcriptional regulation of gene expression
RNA modification
Gene expression regulation
amino acid transmembrane transport
carboxylic acid transmembrane transport
heterocycle biosynthetic process
inositol catabolic process
intracellular transport
lipid transport
macromolecule methylation
modification-dependent macromolecule catabolic process
negative regulation of catalytic activity
nucleobase-containing compound biosynthetic process
nucleoside phosphate metabolic process
nucleotide metabolic process
organophosphate biosynthetic process
pectin catabolic process
phenylpropanoid biosynthetic process
polysaccharide catabolic process
secondary metabolic process
sporopollenin biosynthetic process
Metabolism and transport
establishment of protein localization
establishment of protein localization to organelle
peptidyl-lysine modification
protein catabolic process
protein localization to organelle
protein transport
regulation of protein metabolic process
rRNA processing
translation
tRNA metabolic process
tRNA modification
tRNA processing
Protein turnover and modification
cellular response to hypoxia
cellular response to sulfur starvation
regulation of pH
response to abscisic acid
response to auxin
response to light intensity
response to lipid
response to osmotic stress
response to salt stress
response to water deprivation
response to wounding
Signaling and stress response
FDR
2.5
5.0
7.5
10.0
12.5
F
D5 D6 D7
chromatin
nuclear body
nuclear chromosome
nuclear envelope
nuclear protein-containing complex
nucleolus
nucleoplasm
Nucleus
ATPase complex
chloroplast envelope
chloroplast stroma
chloroplast thylakoid membrane
mitochondrial matrix
mitochondrial respiratory chain complex I
mitochondrion
organelle inner membrane
Double membrane organelle
anchored component of membrane
apical plasma membrane
endoplasmic reticulum membrane
endosome
extracellular region
Golgi cisterna
Golgi trans cisterna
integral component of plasma membrane
membrane protein complex
microtubule cytoskeleton
plasmodesma
pollen coat
pollen tube tip
trans-Golgi network
Membrane system/ Extracellular region
monolayer-surrounded lipid storage body
plant-type vacuole
Single membrane organelle
Cul4-RING E3 ubiquitin ligase complex
cullin-RING ubiquitin ligase complex
cytosolic large ribosomal subunit
peptidase complex
supramolecular complex
Large complex
Unclassified
Unclassified
Nucleus
Double membrane organel
Membrane/Extracellular
Single membrane organelle
Cytoskeleton
Large complex
Unclassified
FDR
2.5
5.0
7.5
10.0

Fig S17. Aging-related genes at early and late phases and the summary of all 6491 ARGs.
A-B. Total number of ARGs on Day 5, 6, and 7. **A**: up-regulated genes; **B**: down-regulated genes. **C**. Comparison of total number of ARGs from Day 5, 6 and 7. Left panel: up-regulated genes; right panel: down-regulated genes. **D**. All 6491 aging-related genes. **E**. GO enrichment of biological processes of ARGs from **C** (up-regulated). Colors of the dots refer to different general biological processes, and sizes of the dots refer to the level of enrichment (-$\log_{10}$FDR). **F**. GO enrichment of cellular components of ARGs from **C** (up-regulated). Colors of the dots refer to different general cellular components, and sizes of the dots refer to the level of enrichment (-$\log_{10}$FDR). Yellow represents Day 0/5 (expression level in Day 5 compared to Day 0), brown represents Day 0/6 (expression level in Day 6 compared to Day 0), dark brown represents Day 0/7 (expression level in Day 7 compared to Day 0), light green represents Day 5/6 (expression level in Day 6 compared to Day 5), green represents Day 6/7 (expression level in Day 7 compared to Day 6), dark green represents Day 5/7 (expression level in Day 7 compared to Day 5).

Figure S18

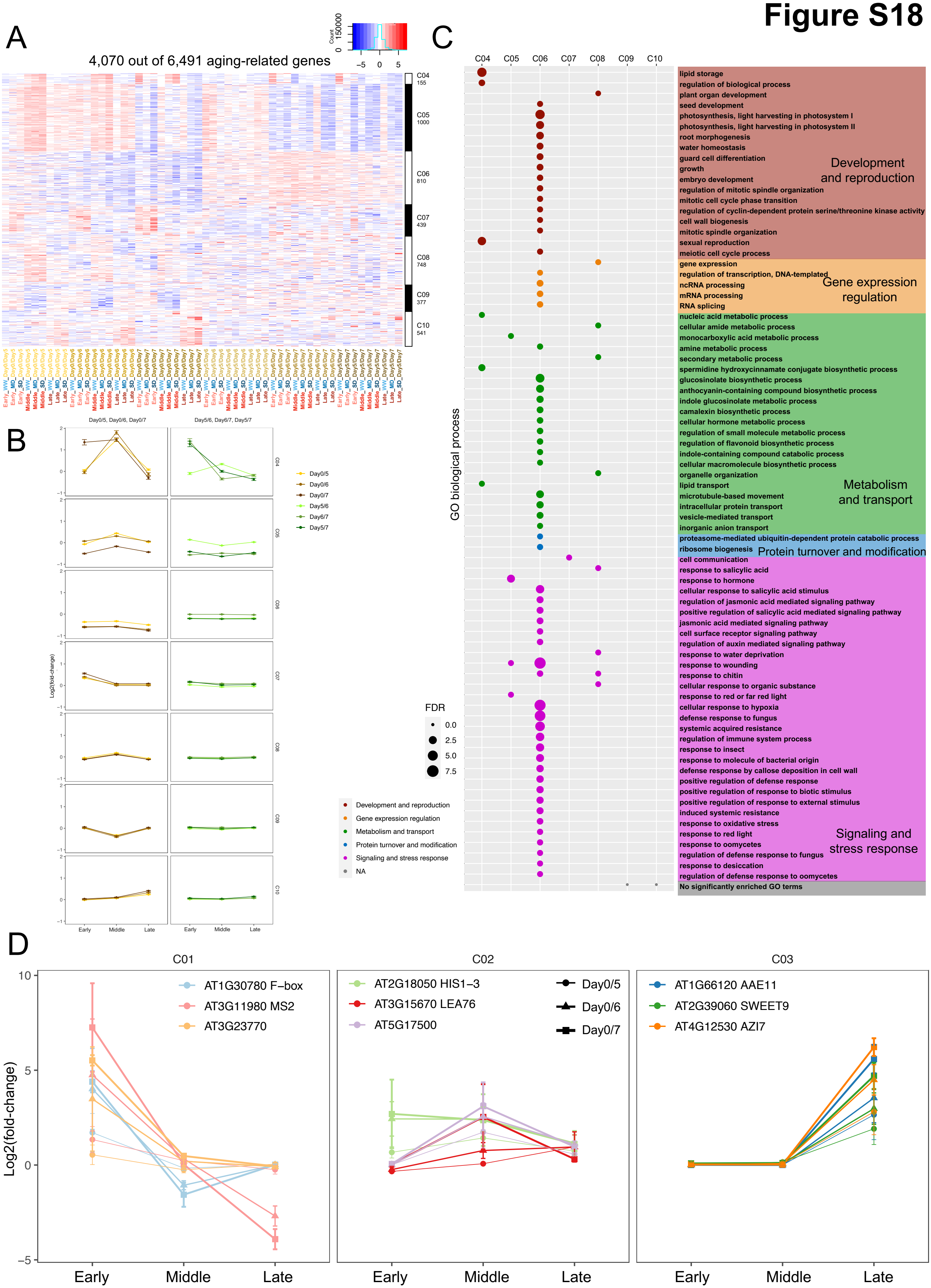
A
4,070 out of 6,491 aging-related genes
C04 155
C05 1000
C06 810
C07 439
C08 748
C09 377
C10 541
B
Day0/5, Day0/6, Day0/7
Day5/6, Day6/7, Day5/7
Day0/5
Day0/6
Day0/7
Day5/6
Day6/7
Day5/7
Log2(fold-change)
Early
Middle
Late
C
GO biological process
FDR
Development and reproduction
Gene expression regulation
Metabolism and transport
Protein turnover and modification
Signaling and stress response
NA
lipid storage
regulation of biological process
plant organ development
seed development
photosynthesis, light harvesting in photosystem I
photosynthesis, light harvesting in photosystem II
root morphogenesis
water homeostasis
guard cell differentiation
growth
embryo development
regulation of mitotic spindle organization
mitotic cell cycle phase transition
regulation of cyclin-dependent protein serine/threonine kinase activity
cell wall biogenesis
mitotic spindle organization
sexual reproduction
meiotic cell cycle process
Development and reproduction
gene expression
regulation of transcription, DNA-templated
ncRNA processing
mRNA processing
RNA splicing
Gene expression regulation
nucleic acid metabolic process
cellular amide metabolic process
monocarboxylic acid metabolic process
amine metabolic process
secondary metabolic process
spermidine hydroxycinnamate conjugate biosynthetic process
glucosinolate biosynthetic process
anthocyanin-containing compound biosynthetic process
indole glucosinolate metabolic process
camalexin biosynthetic process
cellular hormone metabolic process
regulation of small molecule metabolic process
regulation of flavonoid biosynthetic process
indole-containing compound catabolic process
cellular macromolecule biosynthetic process
organelle organization
lipid transport
microtubule-based movement
intracellular protein transport
vesicle-mediated transport
inorganic anion transport
Metabolism and transport
proteasome-mediated ubiquitin-dependent protein catabolic process
ribosome biogenesis
Protein turnover and modification
cell communication
response to salicylic acid
response to hormone
cellular response to salicylic acid stimulus
regulation of jasmonic acid mediated signaling pathway
positive regulation of salicylic acid mediated signaling pathway
jasmonic acid mediated signaling pathway
cell surface receptor signaling pathway
regulation of auxin mediated signaling pathway
response to water deprivation
response to wounding
response to chitin
cellular response to organic substance
response to red or far red light
cellular response to hypoxia
defense response to fungus
systemic acquired resistance
regulation of immune system process
response to insect
response to molecule of bacterial origin
defense response by callose deposition in cell wall
positive regulation of defense response
positive regulation of response to biotic stimulus
positive regulation of response to external stimulus
induced systemic resistance
response to oxidative stress
response to red light
response to oomycetes
regulation of defense response to fungus
response to desiccation
regulation of defense response to oomycetes
Signaling and stress response
No significantly enriched GO terms
D
C01
AT1G30780 F−box
AT3G11980 MS2
AT3G23770
C02
AT2G18050 HIS1−3
AT3G15670 LEA76
AT5G17500
Day0/5
Day0/6
Day0/7
C03
AT1G66120 AAE11
AT2G39060 SWEET9
AT4G12530 AZI7
Log2(fold-change)
Early
Middle
Late

Fig S18. The other 6 clusters of ARGs and transcriptional regulation of plant age.
A. Expression dynamics under the 3 different water conditions and across the 3 developmental phases of the other 6 clusters (C04-C10) including 4070 ARGs. Red indicates up-regulation, blue indicated down-regulation and white indicates no change. **B**. The average fold change of all genes in the corresponding clusters during reproductive development on different days (the 3 different water were combined). Light yellow represents Day 0/5, yellow represents Day 0/6, and dark yellow represents Day0/7, light green represents Day 5/6, green represents Day 6/7, dark green represents Day 5/7. **C**. GO enrichment based on genes from the corresponding clusters. Colors of the dots refer to different general biological processes, and sizes of the dots refer to the level of enrichment (-$\log_{10}$FDR). **D**. The fold change of representative genes from the corresponding clusters on different days at the 3 developmental phases (the 3 different water were combined). Circle represents Day 0/5; triangle represents Day 0/6; square represents Day 0/7. Each color represents a different gene.

Figure S19

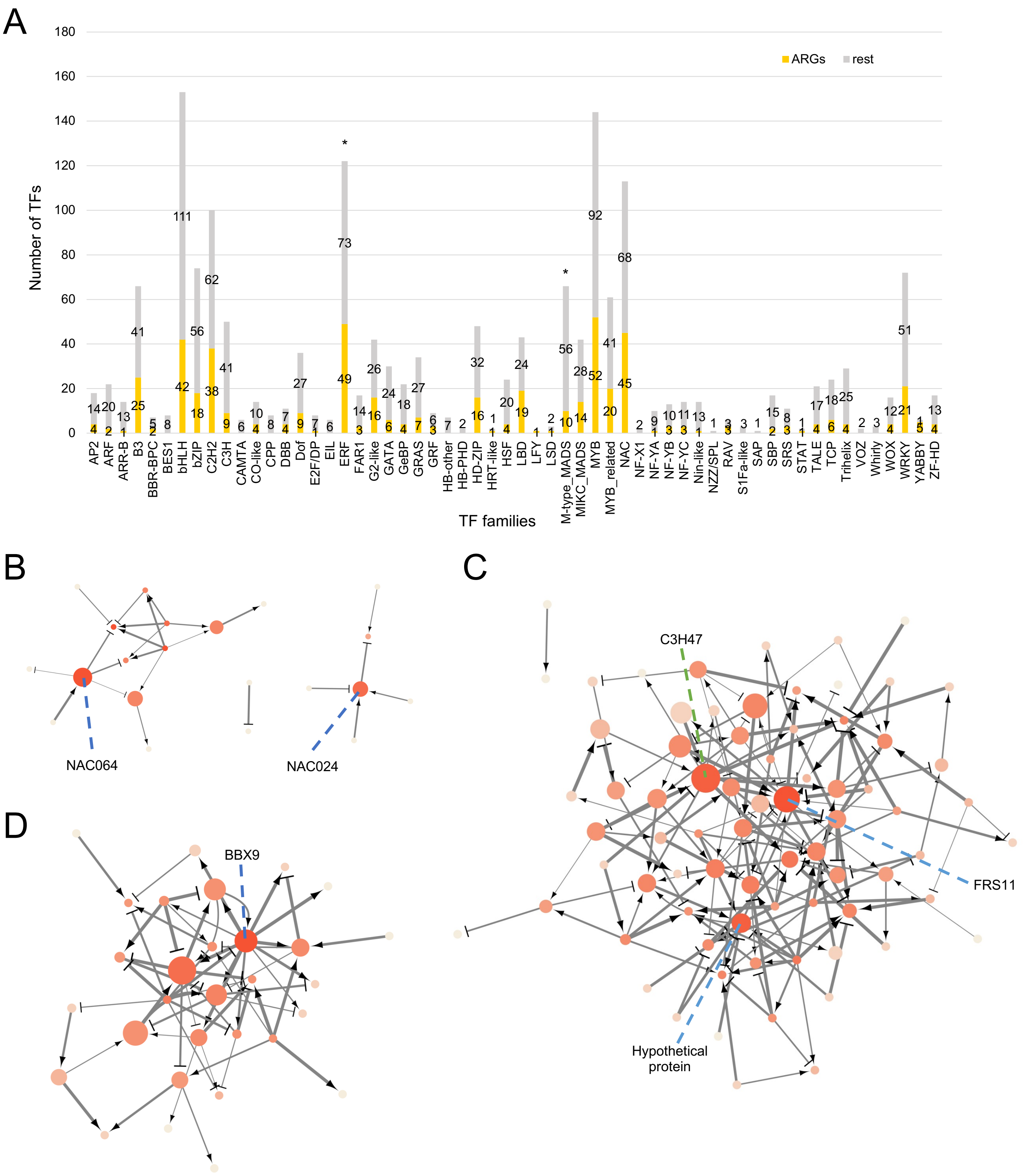

Fig S19. Known genes and GRNs of TFs from the 3 representative ARG clusters.

A. The TF distribution across all TF families of the age-related TFs. * indicates *p*-value < 0.05. Yellow bar indicates the TFs showed differential expression during development in each TF family, grey bar indicates the rest of the TFs in the same family. **B-D**. Gene regulatory networks based on the TFs from Cluster 01, 02, and 03. Node color represents the degree of interaction (the number of edges of a node), node size represents the betweenness of the node (the frequency that this node acts as a bridge along the shortest path between two other nodes), and the edge width represents the weight of the interaction (the probability of the interaction (posterior link probability) that was obtained based on the fold change of the genes in response to drought stress). Arrows refer to positive regulation from the regulators to the targets, and the “T”-end lines refer to negative regulation from the regulators to the targets. The central nodes were labeled. The green dashed lines refer to previously studied TFs with flower development related functions, and the blue dashed lines refer to TFs with no known function during flower development.

Figure S20

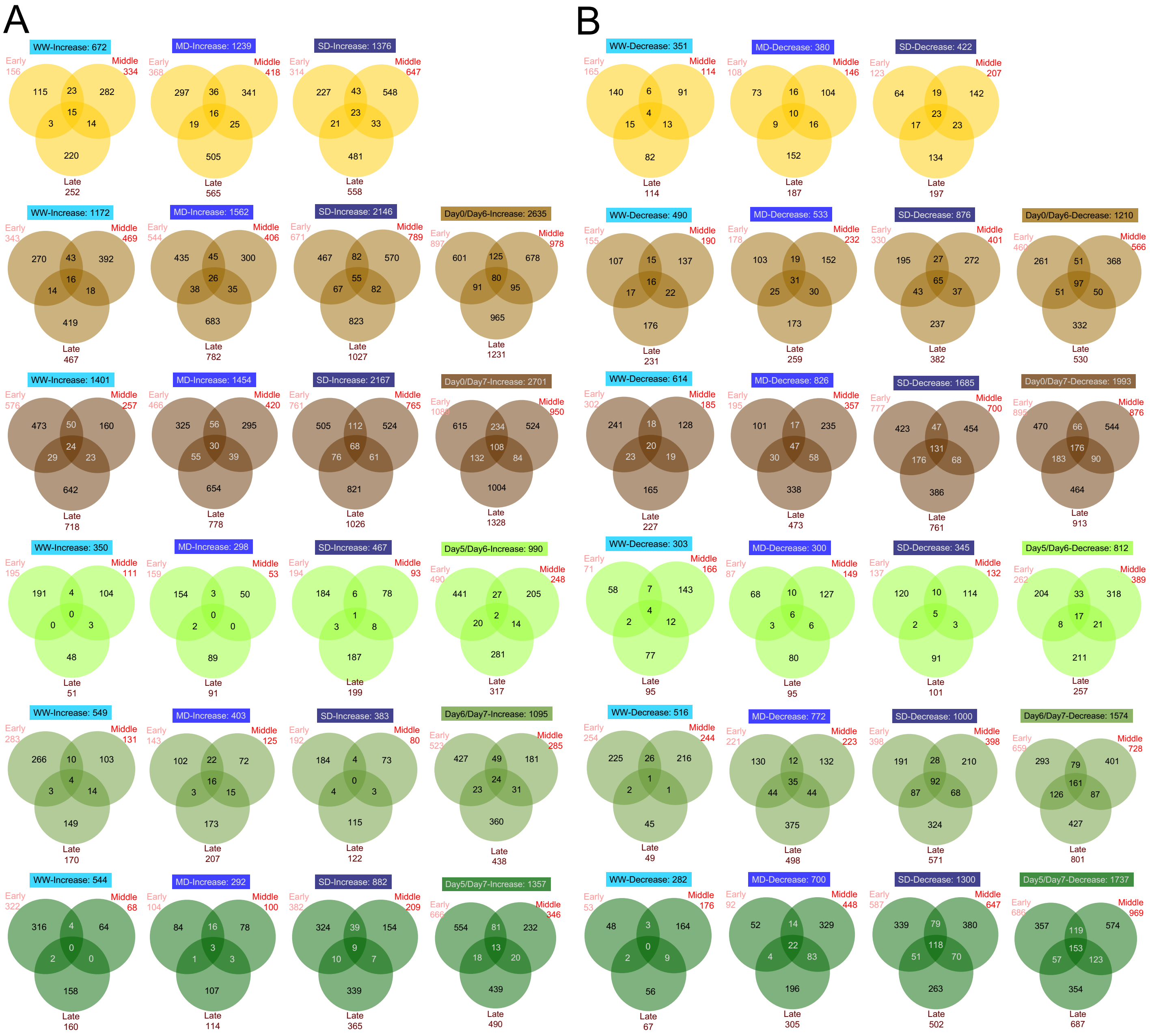

Fig S20. Comparison of ARGs between the 3 developmental phases.
A-B. Comparison of ARGs between the 3 different developmental phases under the same water condition and the summarizing comparison. **A**: up-regulated genes; **B**: down-regulated genes. First row: Day 0/5; second row: Day 0/6; third row: Day 0/7; fourth row: Day 5/6; fifth row: Day 6/7; sixth row: Day 5/7. First column: WW; second column: MD; third column: SD; fourth column: all 3 water conditions. Yellow represents Day 0/5 (expression level in Day 5 compared to Day 0), brown represents Day 0/6 (expression level in Day 6 compared to Day 0), dark brown represents Day 0/7 (expression level in Day 7 compared to Day 0), light green represents Day 5/6 (expression level in Day 6 compared to Day 5), green represents Day 6/7 (expression level in Day 7 compared to Day 6), dark green represents Day 5/7 (expression level in Day 7 compared to Day 5).

Figure S21

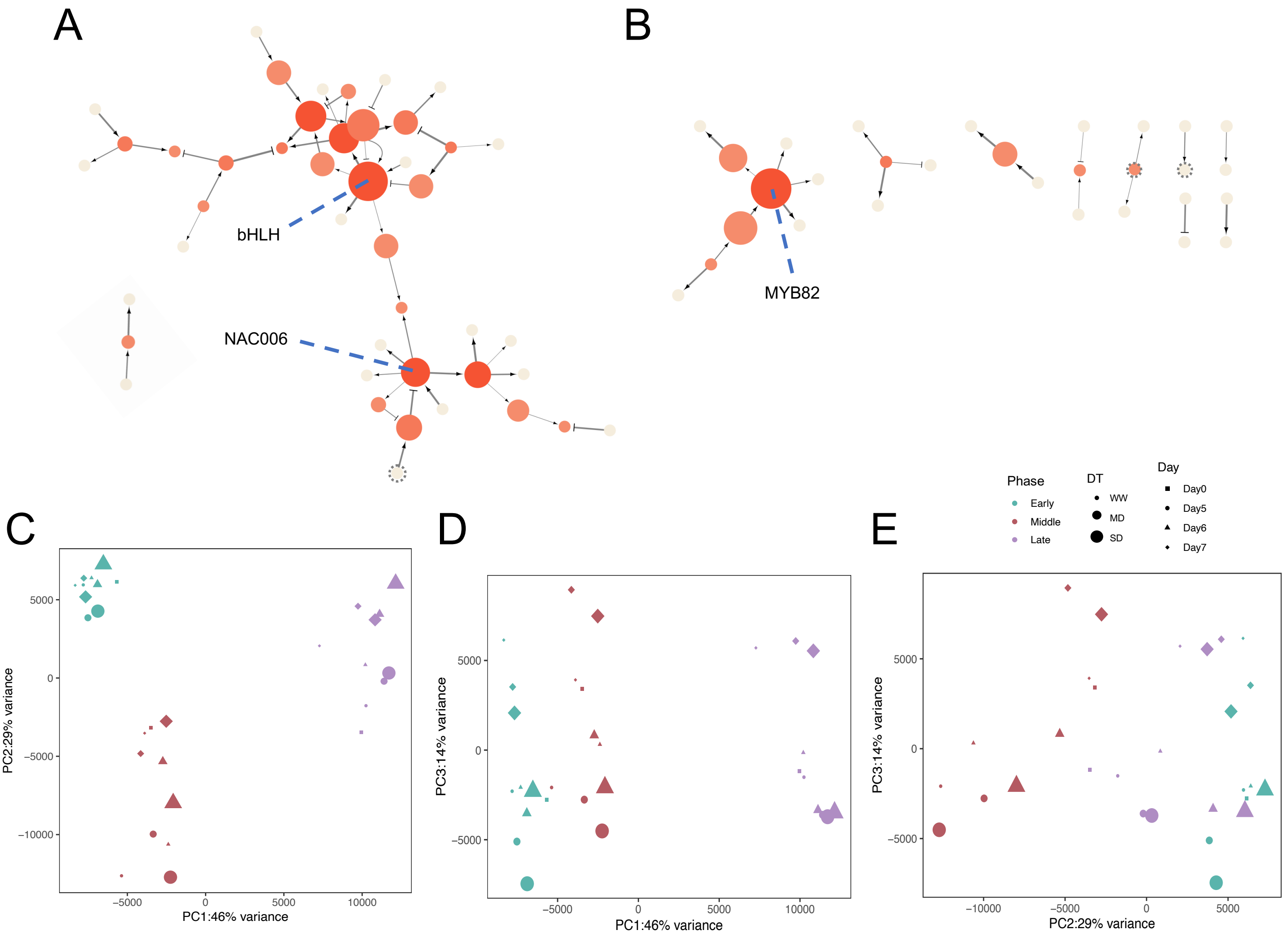

Fig S21. Transcriptome profiling of *Arabidopsis* reproductive development under drought stress over a 3-day period.

A-B: Gene regulatory networks based on the TFs from differentially expressed genes from all 3 tissue/treatment comparisons. **A**: up-regulated genes; **B**: down-regulated genes. Node color represents the degree of interaction (the number of edges of a node), node size represents the betweenness of the node (the frequency that this node acts as a bridge along the shortest path between two other nodes), and the edge width represents the weight of the interaction (the probability of the interaction (posterior link probability) that was obtained based on the fold change of the genes in response to drought stress). Arrows refer to positive regulation from the regulators to the targets, and the “T”-end lines refer to negative regulation from the regulators to the targets. The central nodes were labeled. The blue dashed lines refer to TFs with no known function during flower development. Grey circle indicates that this gene does not have a homolog in representative angiosperms including *Carica papaya*, *Populus trichocarpa*, *Solanum lycopersicum*, *Oryza sativa*, and *Amborella trichopoda*.
C-E. PCA analyses of the 30 samples (water availability, developmental phases and days). **C**: Plot of PC1 and PC2; **D**: Plot of PC1 and PC3; **E**: Plot of PC2 and PC3. Colors represent different developmental phases, shapes represent different days, and sizes represent different water conditions.

Figure S22

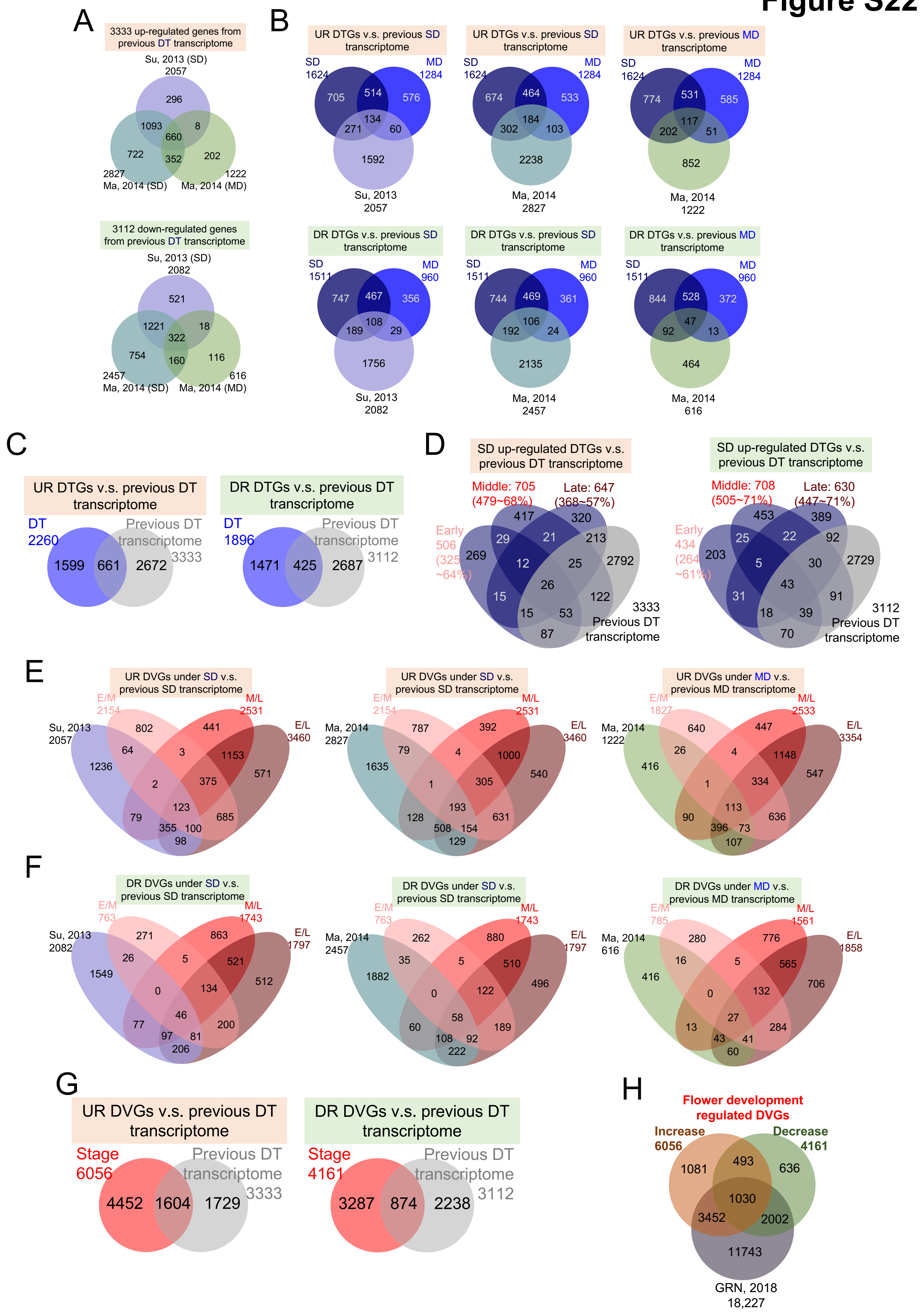
A
3333 up-regulated genes from previous DT transcriptome
Su, 2013 (SD) 2057
296
1093
8
660
722
352
202
2827 Ma, 2014 (SD)
1222 Ma, 2014 (MD)
3112 down-regulated genes from previous DT transcriptome
Su, 2013 (SD) 2082
521
1221
18
322
754
160
116
2457 Ma, 2014 (SD)
616 Ma, 2014 (MD)
B
UR DTGs v.s. previous SD transcriptome
SD 1624
MD 1284
705
514
576
134
271
60
1592
Su, 2013 2057
UR DTGs v.s. previous SD transcriptome
SD 1624
MD 1284
674
464
533
184
302
103
2238
Ma, 2014 2827
UR DTGs v.s. previous MD transcriptome
SD 1624
MD 1284
774
531
585
117
202
51
852
Ma, 2014 1222
DR DTGs v.s. previous SD transcriptome
SD 1511
MD 960
747
467
356
108
189
29
1756
Su, 2013 2082
DR DTGs v.s. previous SD transcriptome
SD 1511
MD 960
744
469
361
106
192
24
2135
Ma, 2014 2457
DR DTGs v.s. previous MD transcriptome
SD 1511
MD 960
844
528
372
47
92
13
464
Ma, 2014 616
C
UR DTGs v.s. previous DT transcriptome
DT 2260
Previous DT transcriptome 3333
1599
661
2672
DR DTGs v.s. previous DT transcriptome
DT 1896
Previous DT transcriptome 3112
1471
425
2687
D
SD up-regulated DTGs v.s. previous DT transcriptome
Middle: 705 (479~68%)
Late: 647 (368~57%)
Early 506 (325~64%)
417
320
29
21
213
269
12
25
2792
26
15
122
15
53
87
3333 Previous DT transcriptome
SD up-regulated DTGs v.s. previous DT transcriptome
Middle: 708 (505~71%)
Late: 630 (447~71%)
Early 434 (264~61%)
453
389
25
22
92
203
5
30
2729
43
31
91
18
39
70
3112 Previous DT transcriptome
E
UR DVGs under SD v.s. previous SD transcriptome
E/M 2154
M/L 2531
E/L 3460
Su, 2013 2057
802
441
64
3
1153
1236
2
375
571
123
79
685
355
100
98
UR DVGs under SD v.s. previous SD transcriptome
E/M 2154
M/L 2531
E/L 3460
Ma, 2014 2827
787
392
79
4
1000
1635
1
305
540
193
128
631
508
154
129
UR DVGs under MD v.s. previous MD transcriptome
E/M 1827
M/L 2533
E/L 3354
Ma, 2014 1222
640
447
26
4
1148
416
1
334
547
113
90
636
396
73
107
F
DR DVGs under SD v.s. previous SD transcriptome
E/M 763
M/L 1743
E/L 1797
Su, 2013 2082
271
863
26
5
521
1549
0
134
512
46
77
200
97
81
206
DR DVGs under SD v.s. previous SD transcriptome
E/M 763
M/L 1743
E/L 1797
Ma, 2014 2457
262
880
35
5
510
1882
0
122
496
58
60
189
108
92
222
DR DVGs under MD v.s. previous MD transcriptome
E/M 785
M/L 1561
E/L 1858
Ma, 2014 616
280
776
16
5
565
416
0
132
706
27
13
284
43
41
60
G
UR DVGs v.s. previous DT transcriptome
Stage 6056
Previous DT transcriptome 3333
4452
1604
1729
DR DVGs v.s. previous DT transcriptome
Stage 4161
Previous DT transcriptome 3112
3287
874
2238
H
Flower development regulated DVGs
Increase 6056
Decrease 4161
1081
493
636
1030
3452
2002
11743
GRN, 2018 18,227

Fig S22. Comparison between DTGs, DVGs with published transcriptome datasets.
A. Summary of previous drought transcriptome datasets in flowers from Ma, 2014 and Su, 2013. Top panel: up-regulated genes; bottom panel: down-regulated genes. **B-C**: Comparison between the DTGs from the drought comparisons and previous drought floral transcriptome. **B**: SD and MD datasets from our study and previous drought floral transcriptomes were both separated: left panel: comparison between Su, 2013 (SD) with DTGs; middle panel: comparison between Ma, 2014 (SD) with DTGs; right panel: comparison between Ma, 2014 (MD) with DTGs. **C**: An overall comparison between DTGs and previous studies: top panel: up-regulated genes; bottom panel: down-regulated genes. **D**. Comparison between DTGs from each developmental phase with previous drought floral transcriptome. **E-F**. Comparison between the DVGs from the phase comparisons and previous drought floral transcriptome. **E**: up-regulated genes; **F**: down-regulated genes. Left panel: comparison between Su, 2013 (SD) with DVGs under SD on all 3 days; middle panel: comparison between Ma, 2014 (SD) with DVGs under SD on all 3 days; right panel: comparison between Ma, 2014 (MD) with DVGs under MD on all 3 days. **G**. Overall comparison between DVGs and previous studies. Left panel: up-regulated genes; right panel: down-regulated genes. **H**. Comparison between the DVGs and previous flower development transcriptome and ChIP-seq data (GRN, 2018). Dark blue represents SD (expression level under SD compared to WW), blue represents MD (expression level under MD compared to WW); light red represents E/M (expression level at middle phase compared to early phase), red represents M/L (expression level at late phase compared to middle phase), dark red represents E/L (expression level at late phase compared to early phase).

Figure S23

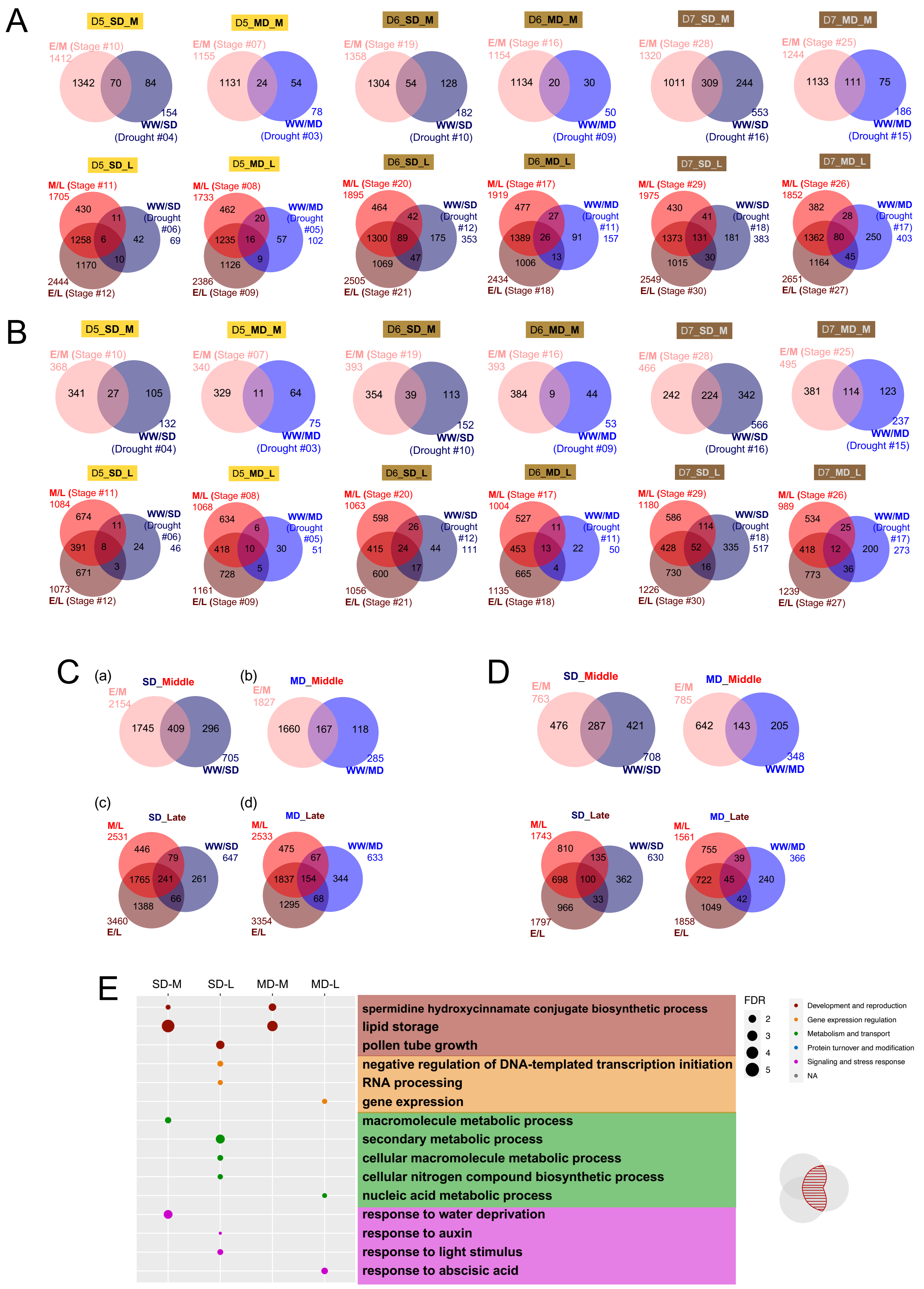

A
D5_SD_M
E/M (Stage #10)
1412
1342 70 84
154
WW/SD
(Drought #04)
D5_MD_M
E/M (Stage #07)
1155
1131 24 54
78
WW/MD
(Drought #03)
D6_SD_M
E/M (Stage #19)
1358
1304 54 128
182
WW/SD
(Drought #10)
D6_MD_M
E/M (Stage #16)
1154
1134 20 30
50
WW/MD
(Drought #09)
D7_SD_M
E/M (Stage #28)
1320
1011 309 244
553
WW/SD
(Drought #16)
D7_MD_M
E/M (Stage #25)
1244
1133 111 75
186
WW/MD
(Drought #15)
D5_SD_L
M/L (Stage #11)
1705
430 11 1258 6 42 1170 10
WW/SD (Drought #06) 69
2444
E/L (Stage #12)
D5_MD_L
M/L (Stage #08)
1733
462 20 1235 16 57 1126 9
WW/MD (Drought #05) 102
2386
E/L (Stage #09)
D6_SD_L
M/L (Stage #20)
1895
464 42 1300 89 175 1069 47
WW/SD (Drought #12) 353
2505
E/L (Stage #21)
D6_MD_L
M/L (Stage #17)
1919
477 27 1389 26 91 1006 13
WW/MD (Drought #11) 157
2434
E/L (Stage #18)
D7_SD_L
M/L (Stage #29)
1975
430 41 1373 131 181 1015 30
WW/SD (Drought #18) 383
2549
E/L (Stage #30)
D7_MD_L
M/L (Stage #26)
1852
382 28 1362 80 250 1164 45
WW/MD (Drought #17) 403
2651
E/L (Stage #27)
B
D5_SD_M
E/M (Stage #10)
368
341 27 105
132
WW/SD
(Drought #04)
D5_MD_M
E/M (Stage #07)
340
329 11 64
75
WW/MD
(Drought #03)
D6_SD_M
E/M (Stage #19)
393
354 39 113
152
WW/SD
(Drought #10)
D6_MD_M
E/M (Stage #16)
393
384 9 44
53
WW/MD
(Drought #09)
D7_SD_M
E/M (Stage #28)
466
242 224 342
566
WW/SD
(Drought #16)
D7_MD_M
E/M (Stage #25)
495
381 114 123
237
WW/MD
(Drought #15)
D5_SD_L
M/L (Stage #11)
1084
674 11 391 8 24 671 3
WW/SD (Drought #06) 46
1073
E/L (Stage #12)
D5_MD_L
M/L (Stage #08)
1068
634 6 418 10 30 728 5
WW/MD (Drought #05) 51
1161
E/L (Stage #09)
D6_SD_L
M/L (Stage #20)
1063
598 26 415 24 44 600 17
WW/SD (Drought #12) 111
1056
E/L (Stage #21)
D6_MD_L
M/L (Stage #17)
1004
527 11 453 13 22 665 4
WW/MD (Drought #11) 50
1135
E/L (Stage #18)
D7_SD_L
M/L (Stage #29)
1180
586 114 428 52 335 730 16
WW/SD (Drought #18) 517
1226
E/L (Stage #30)
D7_MD_L
M/L (Stage #26)
989
534 25 418 12 200 773 36
WW/MD (Drought #17) 273
1239
E/L (Stage #27)
C
(a) SD_Middle
E/M
2154
1745 409 296
705
WW/SD
(b) MD_Middle
E/M
1827
1660 167 118
285
WW/MD
(c) SD_Late
M/L
2531
446 79 1765 241 261 1388 66
WW/SD 647
3460
E/L
(d) MD_Late
M/L
2533
475 67 1837 154 344 1295 68
WW/MD 633
3354
E/L
D
SD_Middle
E/M
763
476 287 421
708
WW/SD
MD_Middle
E/M
785
642 143 205
348
WW/MD
SD_Late
M/L
1743
810 135 698 100 362 966 33
WW/SD 630
1797
E/L
MD_Late
M/L
1561
755 39 722 45 240 1049 42
WW/MD 366
1858
E/L
E
SD-M SD-L MD-M MD-L
spermidine hydroxycinnamate conjugate biosynthetic process
lipid storage
pollen tube growth
negative regulation of DNA-templated transcription initiation
RNA processing
gene expression
macromolecule metabolic process
secondary metabolic process
cellular macromolecule metabolic process
cellular nitrogen compound biosynthetic process
nucleic acid metabolic process
response to water deprivation
response to auxin
response to light stimulus
response to abscisic acid
FDR
2
3
4
5
Development and reproduction
Gene expression regulation
Metabolism and transport
Protein turnover and modification
Signaling and stress response
NA

Fig S23. Comparison between the DVGs and DTGs under the same water condition at the same developmental phase.

A-B. Comparisons of the drought-responsive and developmentally-regulated genes between the corresponding water conditions or phases. The red circle(s) represent(s) the developmentally-regulated genes from the indicated comparison(s) (e.g., the light red circle in the top left diagram in **A** represents the 1412 up-regulated genes from early/middle phase comparison on Day 5 under severe drought), and the blue circle represents the drought-responsive genes from the indicated comparison (e.g., the blue circle in the top left diagram in **A** represents the 154 up-regulated genes from SD/WW water condition comparison on Day5 at middle phase). **A**: up-regulated genes; **B**: down-regulated genes. Top row: middle phase; bottom row: late phase. Left 2 columns: Day 5; middle 2 columns: Day 6; right 2 columns: Day 7. Left panel (of every 2 columns): SD; right panel (of every 2 columns): MD. **C-D**. Comparisons of the drought-responsive and developmentally-regulated genes between the corresponding water conditions or phases of all 3 days. **C**: up-regulated genes; **D**: down-regulated genes. Top row: middle phase; bottom row: late phase. Left panel: SD; right panel: MD. **E**. GO enrichment of the overlapping DTGs and DVGs from **C**, see the schematic for genes analyzed at late phase. Colors of the dots refer to different general biological processes, and sizes of the dots refer to the level of enrichment (-$\log_{10}$FDR). Dark blue represents SD (expression level under SD compared to WW), blue represents MD (expression level under MD compared to WW); light red represents E/M (expression level at middle phase compared to early phase), red represents M/L (expression level at late phase compared to middle phase), dark red represents E/L (expression level at late phase compared to early phase).

Figure S24

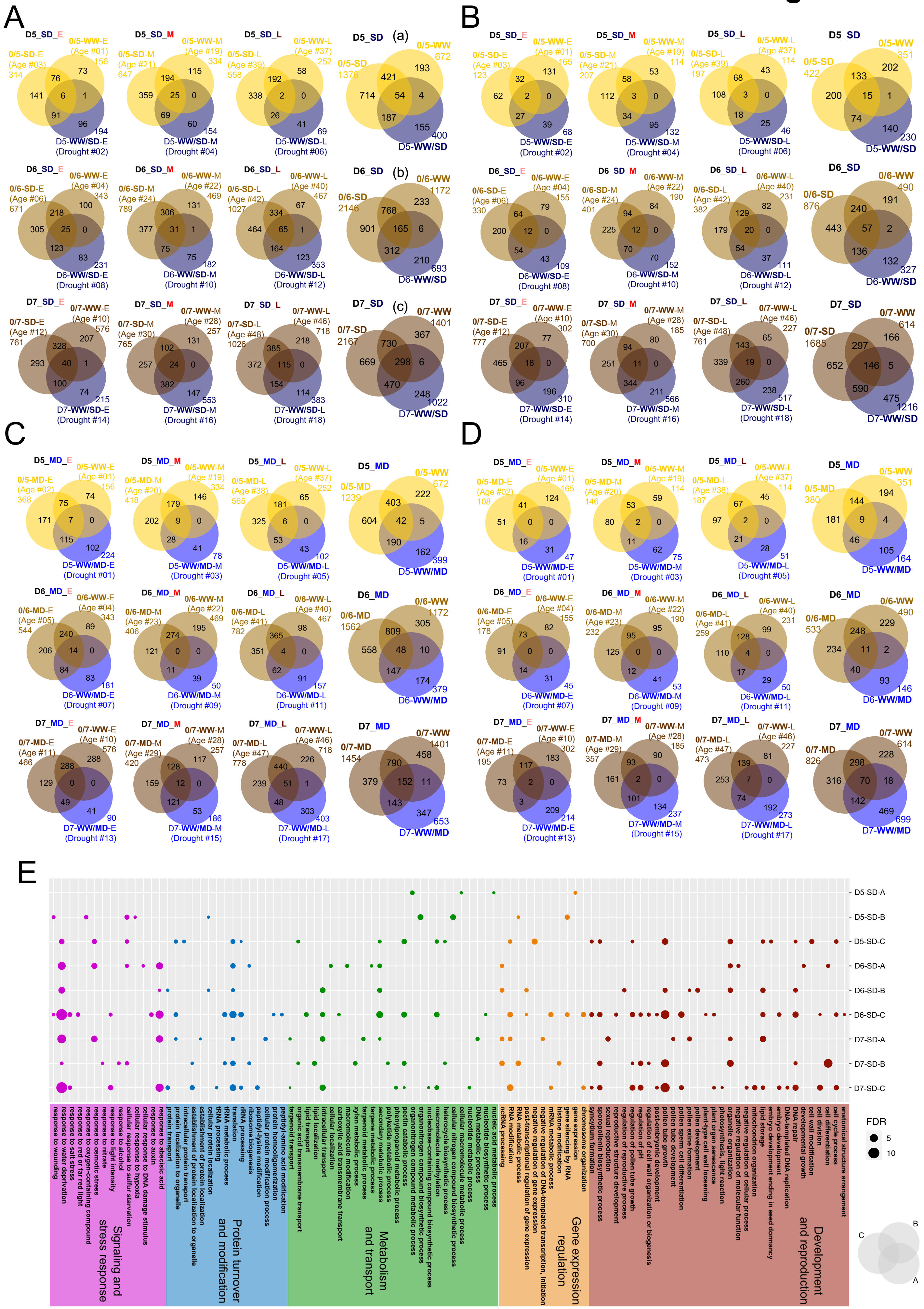

Fig S24. Comparison between the ARGs and DTGs under the same water condition at the same developmental phase.
A-D. Comparison of the drought-responsive and aging-related genes between the corresponding water conditions or days. The yellow circles represent the aging-related genes from the indicated comparisons (e.g., the yellow circles in the top left diagram in **A** represent the 314 and 156 up-regulated genes from Day 0/5 comparisons under SD and WW condition at early phase, respectively), and the blue circle represents the drought-responsive genes from the indicated comparison (e.g., the dark blue circle in the top left diagram in **A** represents the 194 up-regulated genes from SD/WW comparison on Day5 at early phase). **A-B**: SD; **C-D**: MD. **A, C**: up-regulated genes; **B, D**: down-regulated genes. Top row: Day 5; middle row: Day 6; bottom row: Day 7. First column: early phase; second column: middle phase; third column: late phase; fourth column: all 3 phases. **E**. GO enrichment of specific subsets of ARGs from the fourth column of diagrams in **A**. The latter letter refers to the corresponding parts of the Venn diagram as marked in the schematic: A=DTGs, B=ARGs under WW, C=ARGs under SD. Colors of the dots refer to different general biological processes, and sizes of the dots refer to the level of enrichment ($-\log_{10}$FDR). Dark blue represents SD (expression level under SD compared to WW), blue represents MD (expression level under MD compared to WW); yellow represents Day 0/5 (expression level in Day 5 compared to Day 0), brown represents Day 0/6 (expression level in Day 6 compared to Day 0), asparagus represents Day 0/7 (expression level in Day 7 compared to Day 0).
